# The Chlorido-Bismuth Dication: A Potent Lewis Acid Captured in a Hepta-Coordinate Species with a Stereochemically Active Lone Pair

**DOI:** 10.1021/acs.inorgchem.4c01076

**Published:** 2024-06-20

**Authors:** Ahmed Fetoh, Felipe Fantuzzi, Crispin Lichtenberg

**Affiliations:** † Department of Chemistry, Philipps-University Marburg, Hans-Meerwein-Str. 4, Marburg 35032, Germany; ‡ Department of Chemistry, Faculty of Science, Mansoura University, El Gomhouria, Mansoura Qism 2, Dakahlia Governorate 11432 Mansoura, Egypt; § School of Chemistry and Forensic Science, University of Kent, Park Wood Road, Canterbury CT2 7NH, U.K.

## Abstract

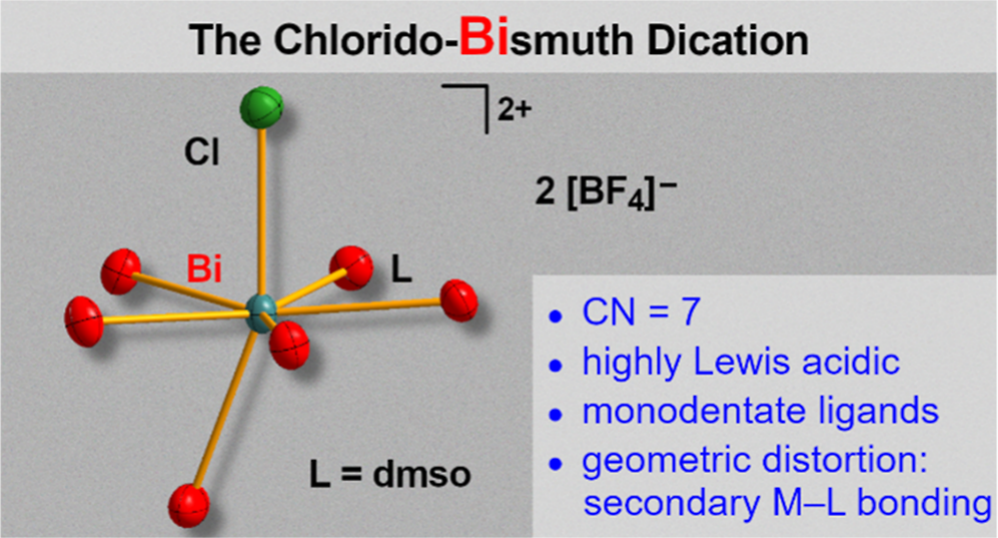

The stabilization of simple, highly reactive cationic species in molecular complexes represents an important strategy to isolate and characterize compounds with uncommon or even unprecedented structural motifs and properties. Here we report the synthesis, isolation, and full characterization of chlorido-bismuth dications, stabilized only by monodentate dimethylsulfoxide (dmso) ligands: [BiCl(dmso)_6_][BF_4_]_2_ (**1**) and [BiCl(μ_2_-dmso)(dmso)_4_]_2_[BF_4_]_4_ (**2**). These compounds show unusual distorted pentagonal bipyramidal coordination geometries along with high Lewis acidities and have been analyzed by multinuclear NMR spectroscopy, elemental analysis, IR spectroscopy, single-crystal X-ray diffraction, and density functional theory calculations. Attempts to generate the bromido- and iodido-analogs gave dmso-stabilized tricationic bismuth species.

## Abstract 2

Here we report the synthesis, isolation, and full characterization of the chlorido-bismuth dication, stabilized only by monodentate dimethylsulfoxide (dmso) ligands: [BiCl(dmso)_6_][BF_4_]_2_ (**1**). This compound shows an unusual distorted pentagonal bipyramidal coordination geometry, which also in the gas phase represents the thermodynamic minimum for calculated [BiCl(dmso)_6_]^2+^. Compound **1** exhibits a remarkably high Lewis acidity and a hard character according to the Pearson concept.

## Introduction

A fascinating field of study in the chemistry of Group 15 elements, focusing on bonding and reactivity, revolves around cationic compounds with unusual coordination numbers.^1^ It allows exploring the boundaries of coordination chemistry of this class of compounds and has uncovered unexpected properties and reactivity patterns. Examples include the isolation of species in unusual oxidation states,^2^ the exploration of strongly Lewis acidic complexes,^1f,3^ new structural motifs,^4^ small molecule activation,^5^ polymer functionalization,^6^ and catalytic applications.^1c,1d,3a,5c,7^

Compounds with high coordination numbers of up to nine have been reported for dicationic bismuth(III) compounds due to the large ionic and covalent radii of the central atom. So far, this class of compounds is only little explored, when compared to the neutral and monocationic parent compounds. Fundamental aspects such as the preferred coordination number and coordination geometry of these compounds, the stereochemical (in)activity of the lone pair at the central atom, and the impact of weak Bi–anion interactions on parameters such as coordination geometry and Lewis acidity have not been studied in great detail, and thus remain difficult or impossible to predict.

**Chart 1:**
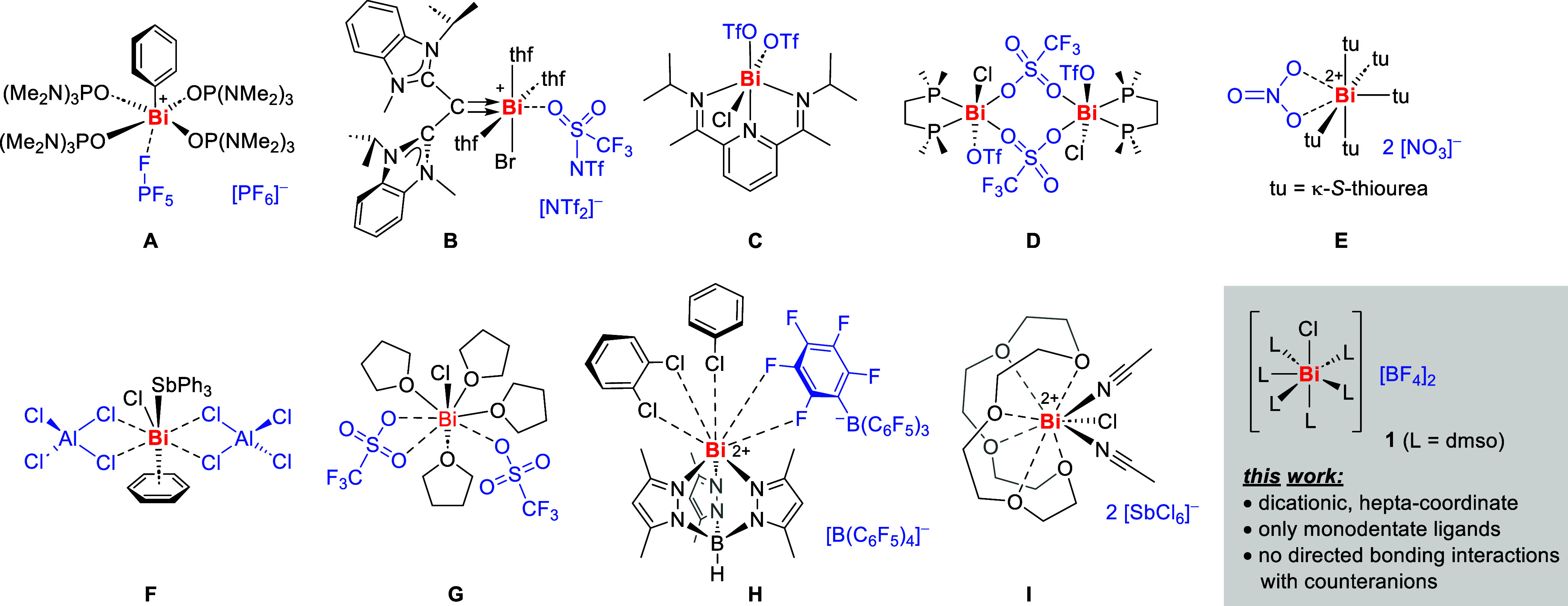
Dicationic Bismuth Complexes, Including Examples That Show (Weak) Bonding Interactions with Counteranions (Shown in Blue)a

The analysis of literature-known di- and tricationic bismuth(III) compounds according to their coordination chemical properties in the solid state reveals a range of species with a coordination number of five. For instance, the complexes [BiPh{OP(NMe_2_)_3_}_4_][PF_6_]_2_ (**A**) and [BiBr(CDC)(thf)_3_][NTf_2_]_2_ (**B**) have been reported, which show five primary bonding interactions of the central atom (Chart 1; CDC = carbodicarbene).^4a,8^ This may be interpreted as a square-based pyramidal coordination geometry with the carbon-based donor in the apical position and has been suggested to hint at a stereochemically active lone pair in *trans*-position to this ligand. It must be noted, however, that there is a contact between the bismuth center and a weakly coordinating counteranion in each of these cases. If this interaction is considered as part of the bismuth coordination sphere, it results in a distorted octahedral geometry.^4a,8^

a The Lewis structures of compounds **A**–**I** shown in this chart reflect their coordination chemistry in the solid state, but in some examples the coordination number in solution might be different, as exemplified for [BiPh{OP(NMe_2_)_3_}_4_][PF_6_]_2_ (**A**) and [BiCl(OTf)_2_(dimpy)] (**C**), for instance.^8,9^

Six-coordinate dicationic bismuth species include the octahedrally coordinated compounds [BiCl(OTf)_2_(dimpy)] (**C**) and [BiCl(OTf)_2_(dmpe)]_2_ (**D**), which show significant bonding interactions with the counteranions (dimpy = diiminopyridine; dmpe = dimehtylphosphinoehtylene).^9−11^ The thiourea complex [Bi(NO_3_){SC(NH_2_)_2_}_5_][NO_3_]_2_ (**E**) has also been argued to display a pseudo-octahedral coordination geometry and a stereochemically inactive lone pair.^12,13^

Examples of dicationic bismuth complexes with coordination numbers of seven and eight are rare and can only be found, when including species that bear chelating ligands and show interactions with their weakly coordinating counteranions, or even additional intermolecular contacts in the solid state, such as compounds [BiCl(AlCl_4_)_2_(SbPh_3_)(C_6_H_6_)] (**F**), [BiCl(κ^1^-OTf)(κ^2^-OTf)(thf)_4_] (**G**), and [Bi(TpMe_2_)(BArF_20_)(C_6_H_4_Cl_2_)(C_6_H_5_Cl)][BArF_20_] (**H**) ( TpMe_2_ = hydridotris(3,5-dimethylpyrazolyl)borate).^1c,7a,14^

Compounds with an even higher coordination number of nine can be obtained by exploitation of crown ethers as chelating ligands. The dicationic 9-coordinate complex [BiCl(18-crown-6)(CH_3_CN)_2_][SbCl_6_]_2_ (**I**) displays a bicapped distorted prismatic coordination geometry, for which a stereochemical activity of the lone pair has been argued to be possible.^15^

A more profound understanding of the coordination chemistry of dicationic bismuth compounds would help to rationalize the impact of the bismuth-centered lone pair on coordination geometries and to deliberately design complexes for potential applications, including nonlinear optics,^16^ catalysis, and the precise design of compounds with distinct Lewis acidic properties. Recently investigated concepts to fine-tune the Lewis acidity of bismuth compounds^17^ comprise the exploitation of geometric constraint,^18^ the installation of electron-withdrawing substituents,^18a,19^ and the utilization of cationic species.^20^ While a softly Lewis acidic character of considerable strength has been assigned to a range of monocationic bismuth compounds,^21^ the quantification of the hard/soft character and strength of dicationic bismuth compounds is virtually unexplored.

Here we present the synthesis, isolation, and full characterization of the hepta-coordinate chlorido-bismuth dications, [BiCl(dmso)_6_][BF_4_]_2_ (Chart 1) and [BiCl(μ_2_-dmso)(dmso)_4_]_2_[BF_4_]_4_, along with the evaluation of their Lewis acidity and the unexpected products obtained from attempts to access the bromido- and iodido-analogs.

## Results and Discussion

**Scheme 1:**
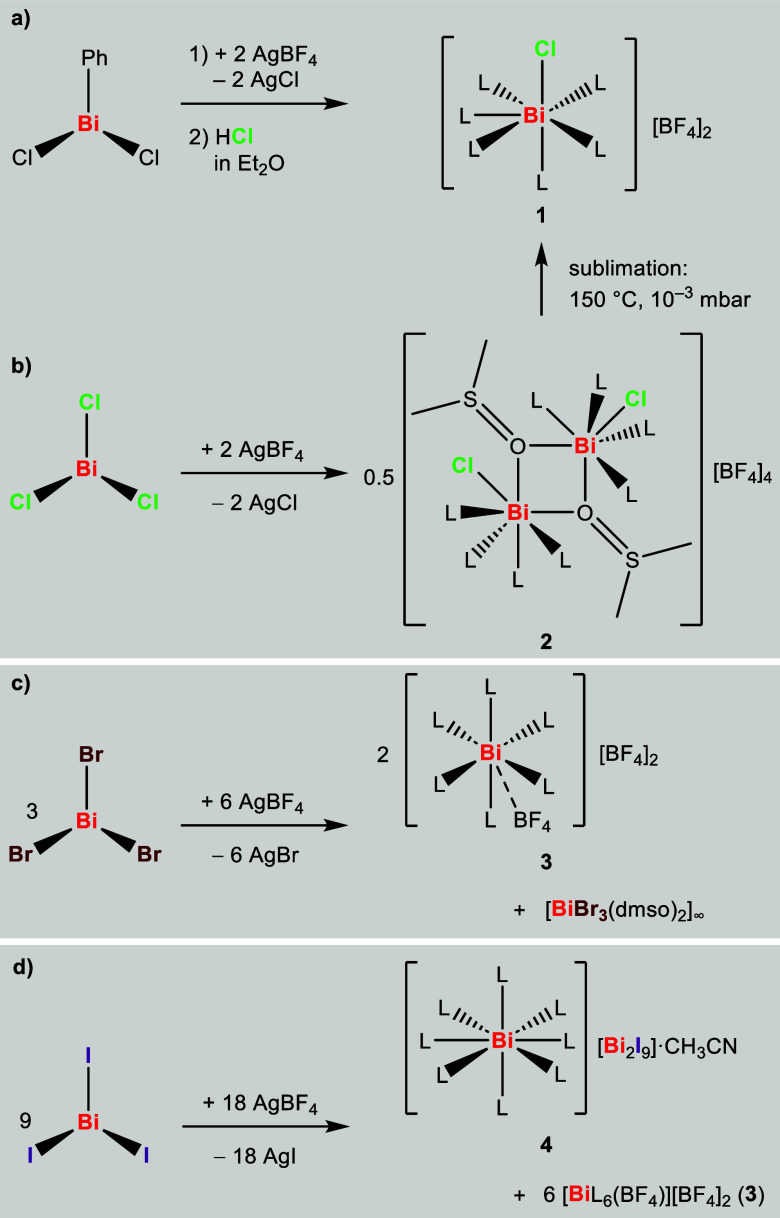
(a) Synthesis of **1** from BiCl_2_Ph, HCl, and AgBF_4_; (b–d) Reactions of BiX_3_ with 2 equiv; AgBF_4_ (X = Cl, Br, I) to Give Compounds **2**–**4**; L = Coordinated dmso; DMSO Is Used as a Solvent in All Reactions

Starting from the easily accessible phenylbismuth dichloride, the addition of two equivalents of AgBF_4_ in DMSO, and subsequent protonolysis with a solution of hydrogen chloride in diethyl ether,^22^ afforded a straightforward method for the preparation of the rare chlorido-bismuthenium dication [BiCl(dmso)_6_][BF_4_]_2_ (**1**) (Scheme 1a). Remarkably, a sublimation approach could also successfully be employed in order to obtain crystals of **1** that were suitable for single-crystal X-ray diffraction analysis (XRD). Specifically, the crude product of **1** was sublimed at 150 °C and 10^–3^ mbar onto a cold finger (−80 °C), yielding pure **1** as a colorless crystalline material. Direct reaction of BiCl_3_ with two equivalents of AgBF_4_ initially gives a closely related, dinuclear compound, namely [Bi_2_Cl_2_(dmso)_10_][BF_4_]_4_ (**2**) (Scheme 1b). Compound **2** was crystallized by layering an acetonitrile solution of this compound with diethyl ether and storage at −30 °C. High-temperature vacuum treatment of isolated **2** gave compound **1** via sublimation of the mononuclear ionic species.

In contrast, the heavier homologues BiBr_3_ and BiI_3_ gave entirely different products, when reacted with AgBF_4_ under conditions identical to those applied for the synthesis of **2**. When BiBr_3_ was treated with AgBF_4_, a mixture of [Bi(dmso)_6_(BF_4_)][BF_4_]_2_ (**3**) and [BiBr_3_(dmso)_2_]_∞_ was obtained as determined by single-crystal X-ray diffraction analysis since both crystals have different shapes and both compounds could be identified by crystal-picking (Scheme 1c). By employing a 1:3 molar ratio, it is possible to selectively generate and isolate **3** in a rational synthetic approach. The reaction of BiI_3_ with AgBF_4_ gave [Bi(dmso)_8_][Bi_2_I_9_]·CH_3_CN (**4**) along with **3**, which could be separated by fractional crystallization (Scheme 1d).

The ^11^B and ^19^F NMR spectra of all compounds featuring [BF_4_]^−^ anions indicate the absence of strong directional bonding interactions between one (or more) of the [BF_4_]^−^ anions and the cation in solution. The ^1^H NMR spectra of all compounds show a singlet for the dmso ligands in the range from 2.55 to 2.79 ppm. This corresponds to a low-field shift of 0.08–0.32 ppm compared to free DMSO in MeCN-*d*_3_ (δ = 2.47 ppm) and indicates the presence of oxygen-coordinated dmso. It has been shown that chemical shifts of 2.59 to 3.03 ppm can be associated with oxygen-coordinated dmso ligands, while sulfur-coordinated dmso ligands tend to resonate in the range of 3.30 to 3.80 ppm.^23^ The ^13^C NMR chemical shifts of all compounds discussed here range from 39.1 to 41.2 ppm compared to free DMSO in MeCN-*d*_3_ (δ = 40.2 ppm).

**Figure 1:**
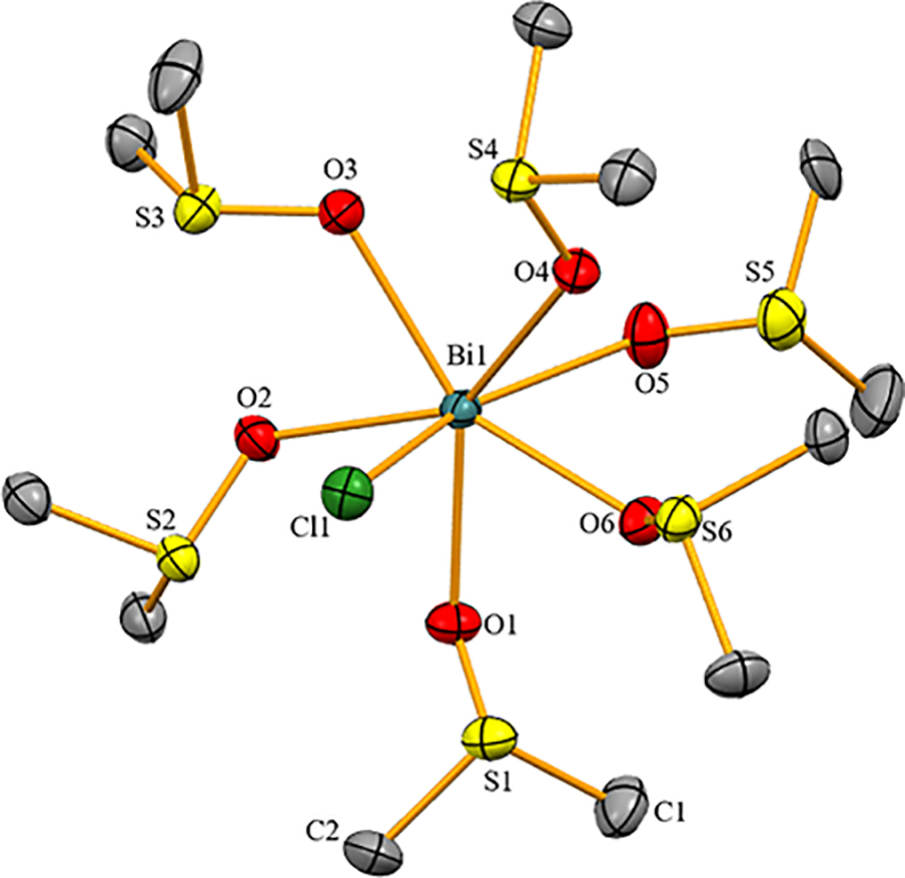
Molecular structure of **1** in the solid state. Displacement ellipsoids are shown at the 50% probability level. Hydrogen atoms, [BF_4_]^−^ counteranions, and split positions are omitted for clarity.

Compound **1** crystallized in the monoclinic space group *P*2_1_/*c* with *Z* = 4 (Figure 1). The bismuth atom in **1** is 7-coordinate without directional bonding interactions to the [BF_4_]^−^ counteranion. The Cl–Bi–O angle clearly deviates from linearity (Cl1–Bi1–O5, 159.10°) and the O–Bi–O angles between neighboring dmso molecules range between 71.22 and 72.69°, when the ligand in *trans*-position to Cl is not taken into account. Thus, **1** adopts a distorted pentagonal bipyramidal coordination geometry. One chlorine atom and one dmso molecule occupy the axial positions, while the remaining dmso molecules are found in the equatorial plane. The Bi–Cl bond in **1** (2.536(12) Å) is longer than that in the first reported example of a monochlorido-bismuth dication (2.479(6) Å), [BiCl([18]crown-6)(CH_3_CN)_2_](SbCl_6_)_2_, or in the dinuclear species [BiCl(OTf)_2_dmpe]_2_ (2.499(2) Å), because i) in **1**, the dmso ligands are not subject to geometric constraints and ii) in **1**, none of the seven Bi–ligand interactions are particularly weak.^14,15^ The Bi–O^dmso^ distances in **1** [2.405(3)–2.606(3) Å] are significantly longer than the sum of the covalent radii [Bi–O = 2.25 Å] but much shorter than the sum of the van der Waals radii [Bi–O = 3.59 Å]. The Bi–O^dmso^ bond of the ligand in the axial position (Bi1–O5, 2.606(3) Å) is significantly longer than those involving dmso ligands in equatorial positions (2.405(3)–2.447(3) Å). This is in line with large bond length variations of neutral ligands in high-coordinate bismuth compounds (e.g., 2.397(3)–2.527(4) Å in [Bi(dmso)_8_]^3+^)^24^ and demonstrates a considerable thermodynamic *trans*-effect experienced by the dmso ligand in the axial position of **1** (based on distance criteria). This can further be supported by comparison with the Bi–O^dmso^ distances in the neutral, (pseudo)hexa-coordinate complexes *fac*-BiCl_3_(dmso)_3_ (2.426(4)–2.461(4) Å),^25^*fac*-BiBr_3_(dmso)_3_ (2.448(5)–2.465(9) Å), and *fac*-Bi(NO_3_)_3_(dmso)_3_ (2.298(3)–2.311(3) Å),^26^ where the Bi–O bond lengths are sensitive to the nature of the ligand in the *trans*-position.^27^

**Figure 2:**
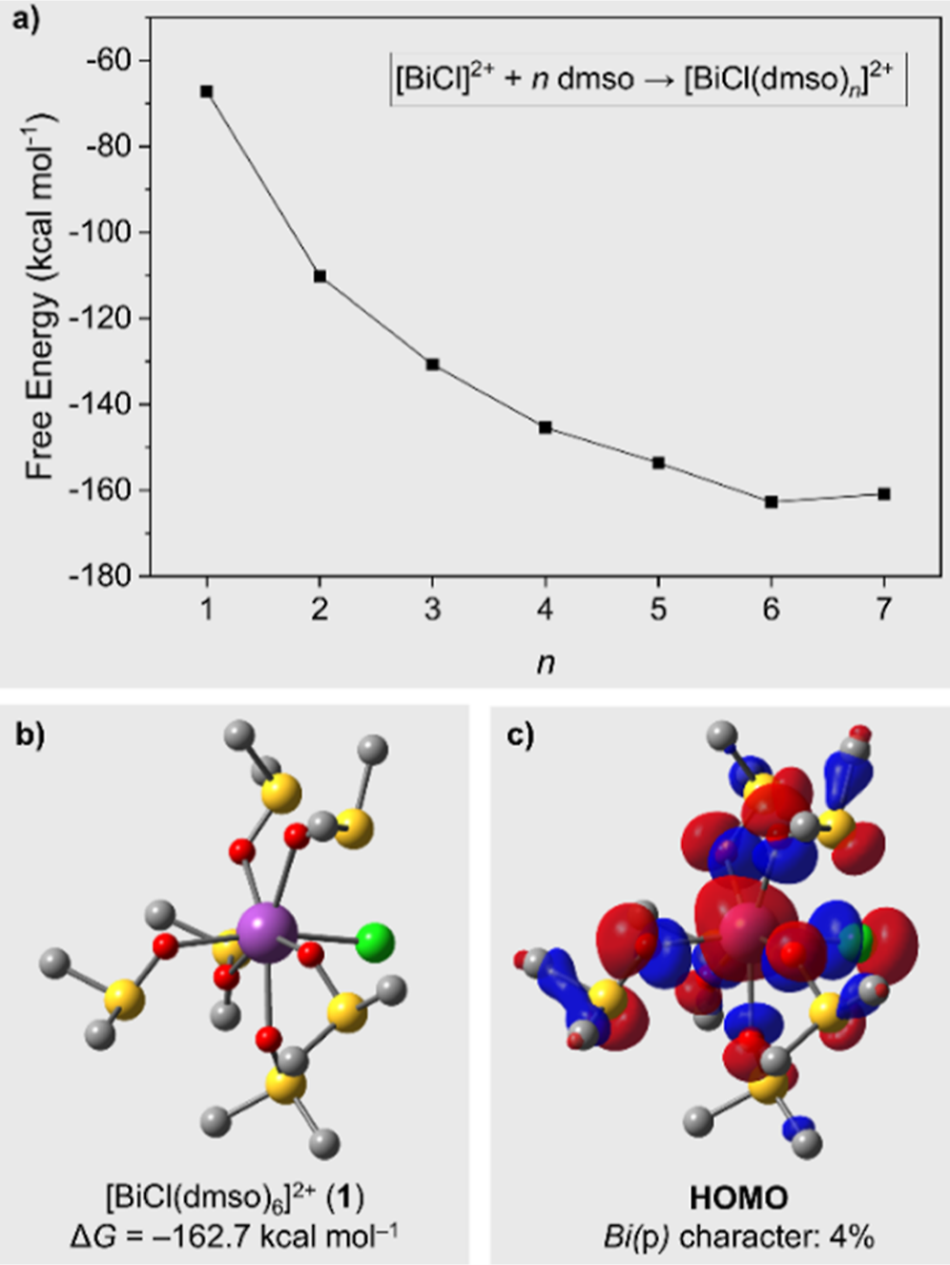
(a) Computed free energy of formation of distinct [BiCl(dmso)_*n*_]^2+^ at the DFT level of theory, indicating compound **1** (*n* = 6) as the most stable structure among those calculated. (b) Optimized structure of **1**. (c) HOMO of compound **1** (isovalue: 0.03 au) and its Bi(p) character.

The unusual coordination number of **1** and the distorted nature of its coordination polyhedron in the dmso-coordinated monochlorido-bismuth dication raise questions about whether these characteristics are due to solid-state packing effects or inherent properties of the molecule. To elucidate this, we studied model compounds of the type [BiCl(dmso)_*n*_]^2+^ (*n* = 1–7) using density functional theory (DFT) calculations (see the Computational Details section for more details). All optimized structures and their corresponding free energies of formation, calculated considering the reaction [BiCl]^2+^ + *n* dmso → [BiCl(dmso)_*n*_]^2+^, are shown in Figure S37 in the Supporting Information (for more details, vide infra). Notably, the experimentally obtained compound demonstrated the most negative free energy of formation among all *n* = 1–7 systems studied. This trend is clearly illustrated in Figure 2a, which compares the computed free energies of formation for the most stable conformations across varying *n* values. Furthermore, this structure (Figure 2b) already contains the distortion observed in the solid state structure of **1**.

**Figure 3:**
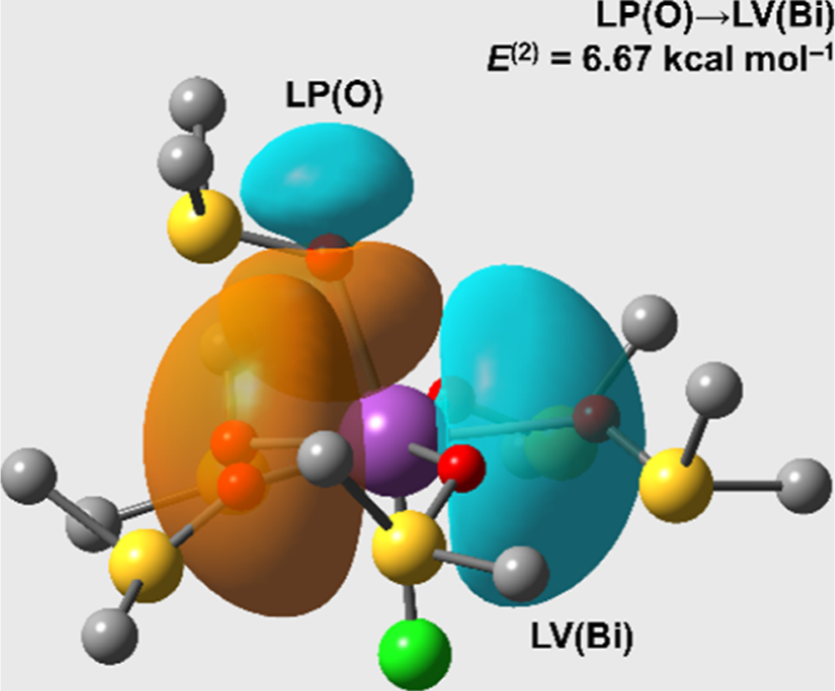
Most important NBO donor–acceptor contribution favoring the distorted structure of **1** in comparison to that where the Cl–Bi–O bond angle is kept at 180°. LP: valence lone pair; LV: lone vacant orbital. Corresponding NBO occupancies: 1.86 e^–^; 0.31 e^–^.

In Figure 2c, we present the HOMO of **1**. Unlike [BiCl_2_(pyridine)_5_][BArF], where the Bi(p) contribution to the HOMO is virtually nonexistent,^1f,28^ our orbital composition analysis using Mulliken partition reveals a small but non-negligible Bi(p) contribution in the HOMO of **1**, accounting for approximately 4%. Overall, the contribution of Bi atomic orbitals to the HOMO amounts to 12%. The bismuth-centered lobe of the HOMO shows a distorted axial orientation, which supports the stereochemical activity of the lone pair at the bismuth atom. Notably, the p(Bi)-orbital contribution to the HOMO diminishes to 0%, when the Cl–Bi–O axis is fixed at an angle of 180°. Furthermore, we examined the reasons behind the structural differences between the distorted pentagonal bipyramidal configuration in [BiCl(dmso)_6_][BF_4_]_2_ and the regular pentagonal bipyramid, seen, for example, in [BiCl_2_(pyridine)_5_][BArF]. To better understand the factors contributing to the distorted pentagonal bipyramidal coordination geometry in [BiCl(dmso)_6_]^2+^, we conducted calculations on both the optimized structure of [BiCl(dmso)_6_]^2+^ and a model system where we constrained the angle between the Cl atom and the dmso ligand in *trans*-position to be 180° (see the Computational Details section for more details). Additionally, we computed their corresponding natural bond orbitals (NBOs) and compared their energy trends. The electronic energy difference between the two structures was merely 1.8 kcal mol^–1^, indicating a preference for the distorted configuration. Moreover, the NBO analysis demonstrated that both Lewis and non-Lewis contributions supported the distorted structure, with each contribution amounting to 0.9 kcal mol^–1^. Notably, the most substantial non-Lewis contribution favoring the distorted structure was a donor–acceptor interaction involving the Bi and O atoms. This interaction featured the valence lone pair (LP) of the oxygen atom in the dmso ligand *trans* to the Cl atom and a lone vacant (LV) orbital of Bi with 100% p character. These orbitals are depicted in Figure 3, which shows that bending of the dmso ligand leads to a more efficient orbital overlap, enhancing this bonding interaction. In summary, our findings underscore that the preference for the distorted structure is already apparent in the electronic structure of the isolated molecule, albeit with a subtle degree of influence.

To the best of our knowledge, compound **1** represents the first example of a monochlorido-bismuth dication stabilized by simple monodentate ligands without directional bonding interactions to the counteranions. This compound expands the series of energetically favorable hepta-coordinate compounds with a lone pair at the central atom, along with Cs[XeF_7_] (capped octahedral, *C*_3*v*_) and [NO_2_][Xe_2_F_13_] (capped trigonal prismatic, *C*_2*v*_),^29^ [Bi(py)_5_Cl_2_][BArF] (pentagonal bipyramidal, *D*_5*h*_).^1f^

Neutral bismuth halides such as BiCl_3_ and BiBr_3_ have successfully been applied as Lewis acid catalysts in organic transformations.^30^ More recent developments have established cationic bismuth compounds as potent Lewis acids and exploited them as key compounds in catalytic reactions.^17,20b,31^ In contrast, dicationic derivatives are only little explored.^1c,1d,20b^ This is likely due to challenges in accessing these species in pure form,^20b^ and consequently, their Lewis acidity is only poorly studied to date. In order to investigate the Lewis acidity of the isolated bismuth dication **1** in solution, the Gutmann–Beckett method (using OPEt_3_ as a reporter molecule) and the modified versions (using SPMe_3_ and SePMe_3_ as reporter molecules) were applied.^21a,32^ A 1:1 molar ratio of compound **1** and the donor EPR_3_ was used in order to mimic realistic scenarios of substrate activation, in which the Lewis acid is commonly not present in excess.

While solvents with poorly Lewis basic properties are usually preferred in the Gutmann–Beckett method, since they do not compete for coordination sites of the Lewis acid, acetonitrile had to be used here in order to ensure solubilization of all reaction partners. Acceptor numbers (AN) for this compound were determined through the ^31^P NMR shifts and calculated according to formulas 1–3 for the respective donor.
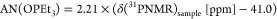
1
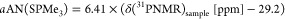
2
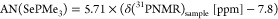
3

With OPEt_3_ as a hard donor, an exceptionally high AN of 92 was obtained (Table 1), which exceeds the ANs of BiCl_3_ (AN = 49),^19^ of monocationic organobismuth species with bulky (AN = 87)^21b^ and less bulk ligands (AN = 59–64),^19^ of a geometrically constrained neutral or cationic species (both: AN ≤ 69),^18b,18c^ of bismuth cations with a mixed aryl/amide ligand environment (AN = 72),^1g^ and of fully nitrogen-supported monocationic bismuth species (AN = 21–51).^1g,27a^ Remarkably, it also clearly outperforms the ANs of a trispyrazolylborate-supported bismuth dication (AN = 75 for 0.25 equiv of OPEt_3_),^1c^ as well as a carbone-stabilized bromido-bismuth dication (AN = 65),^3b^ and carbone-stabilized bismuth trications (AN = 50–84).^3b^ Thus, the hard donor OPEt_3_ is effectively coordinated to the Lewis acidic bismuth center, even in the presence of dmso and an excess of acetonitrile. The interaction of the probe molecule OPEt_3_ with the Lewis acidic bismuth center was confirmed by ESI(+) mass spectrometry (Supporting Information). When the softer donors SPMe_3_ and SePMe_3_ were used as reporter molecules in the modified Gutmann–Beckett method, compound **1** showed moderate interactions according to ^31^P NMR spectroscopy, giving ANs of 24 and 28, respectively (Table 1). These values are significantly lower than those reported for monocationic organobismuth compounds (AN = 76–96),^21^ demonstrating that the dicationic species **1** shows a harder character according to the HSAB principle, when compared to monocationic organobismuth complexes.

**Table 1:** ANs of 1 According to the (Modified) Gutmann–Beckett Method in MeCN-*d*_3_ with a 1:1 Stoichiometry of 1:EPR_3_ (E/R = O/Et, S/Me, Se/Me)

acceptor	donor	δ ^31^P [ppm]	AN
1	OPEt_3_	82.5	92
1	SPMe_3_	33.0	24
1	SePMe_3_	12.7	28

**Figure 4:**
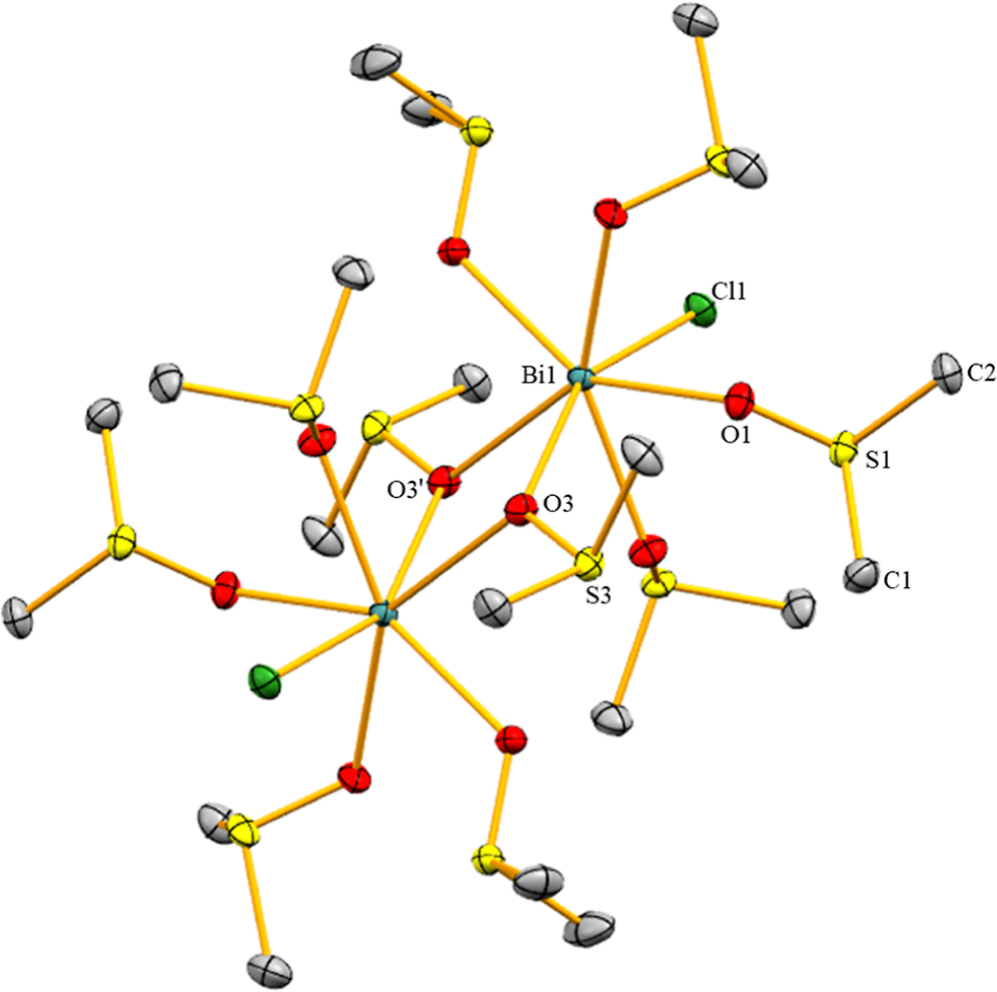
Molecular structure of **2** in the solid state. Displacement ellipsoids are shown at the 50% probability level. Hydrogen atoms, [BF_4_]^−^ counteranions and split positions are omitted for clarity.

Compound **2** crystallizes in the triclinic space group *P*1̅ with *Z* = 2 (Figure 4). Its asymmetric unit shows two crystallographically independent molecules that are chemically identical and show the same trends in bonding parameters, which is why only one of them is discussed in the main text. The bismuth atoms in **2** are hepta-coordinate due to interactions of each bismuth atom with one chlorido ligand, four terminally bound dmso ligands and two μ_2_-bridging dmso ligands. There are no directional bonding interactions with the [BF_4_]^−^ counteranions, creating a fourfold positive charge for the dimeric arrangement [Bi_2_Cl_2_(μ_2_-DMSO)_2_(dmso)_8_]^4+^. The interatomic distance of 4.210(2) Å between two bismuth atoms rules out significant Bi–Bi interactions. The pnictogen centers adopt a distorted pentagonal bipyramidal geometry. The chlorido ligand and one bridging dmso ligand occupy the axial positions, with a Cl1–Bi1–O3 angle of 156.60(4)° giving evidence of the distorted nature of the coordination polyhedron. The O–Bi–O angles involving neighboring dmso ligands in the equatorial plane range from 68.10(8) to 74.88(8)°, the largest deviation from the ideally expected value of 72° expectedly being associated with the bridging dmso ligand (O2–Bi1–O3, 68.10(8)°). The bridging coordination mode of the dmso ligands also affects the bond lengths in **2**. The axial Bi–O^dmso^ bond (Bi1–O3, 2.758(2) Å) is longer than that in **1** (2.606(3) Å), leading to a shorter Bi–Cl bond in **2** (Bi1–Cl1, 2.498(10) Å) compared to **1** (2.536(12) Å). Also, the bridging dmso ligand in the equatorial plane is associated with a larger bond length (Bi1–O3′, 2.663(2) Å) than the terminally bound counterparts (Bi1–O1/2/4/5, 2.328(2)-2.420(2) Å). The Bi–(μ_2_-O) bond lengths (2.663(2) and 2.758(2) Å) are within the broad range of previously reported dmso-bridged bismuth-oxo-clusters, [{Bi_9_O_7_(OH)(O_3_SCH=CH_2_)_11_(dmso)_11_](O_3_SCH=CH_2_)·3 dmso, [Bi_38_O_45_(NO_3_)_8_(O_3_SCH= CH_2_)_14_(dmso)_18_](O_3_SCH=CH_2_)_2_ · 2 dmso, and [Bi_38_O_45_(NO_3_)_6_(OH)_4_(O_3_SCH=CH_2_)_12_(dmso)_23_(H_2_O)_2_] (O_3_SCH=CH_2_)_2_·2H_2_O (ranging from 2.317 to 2.852 Å).^33^ In the IR spectrum of **2**, a very intense and broad band at υ̅ = 896 cm^–1^ corresponds to the stretching vibration of the S=O bond and is characteristic for μ_2_-O-bridging dmso ligands,^34^ as reported for other bismuth dmso compounds.^35^

**Figure 5:**
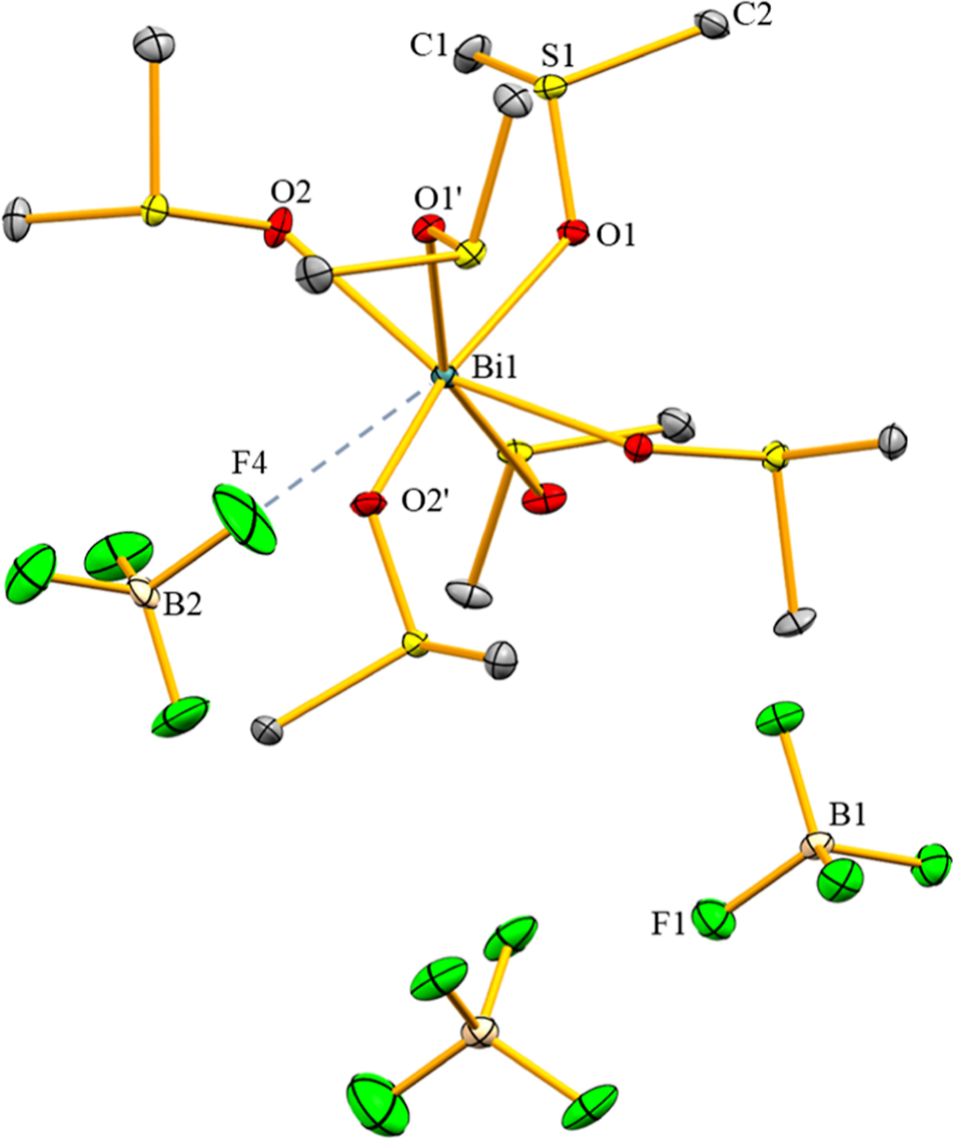
Molecular structure of [Bi(dmso)_6_(BF_4_)][BF_4_]_2_ (**3**) in the solid state. Displacement ellipsoids are shown at the 50% probability level. Hydrogen atoms and split positions are omitted for clarity.

Compound [Bi(dmso)_6_(BF_4_)][BF_4_]_2_ (**3**) crystallized in the trigonal space group *R*3 with *Z* = 3 (Figure 5). It should be noted that bismuth compounds featuring three weakly coordinating counteranions commonly bind eight (rather than six) dmso ligands, as exemplified with the counteranions [ClO_4_]^−^, [Bi_2_I_9_]^3–^, [Fe(NCS)_6_]^3–^, and [Mo_8_O_26_]^2–^.^24,36^ In contrast, compound [Bi(dmso)_6_(BF_4_)][BF_4_]_2_ shows a single-capped octahedron as a coordination polyhedron around the central atom. The regular octahedral positions are occupied by dmso ligands, while one [BF_4_]^−^ moiety is found in the capping position, and two [BF_4_]^−^ counteranions show no directional bonding interaction with the central atom. The Bi–O distances and O–Bi–O angles involving the dmso ligands forming the capped face of the octahedron are larger than the remaining ones (Bi1–O1, 2.302(3) Å; O1–Bi1–O1′, 87.97(10)° vs Bi1–O2, 2.413(3) Å; O2–Bi1–O2′, 113.22(6)°). The [BF_4_]^−^ group acts as monodentate ligand with a Bi···F–BF_3_ distance of 2.695(6) Å. This is larger than Bi···F–SbF_5_ distances in three- or four-coordinate monocationic bismuth compounds (e.g., 2.451–2.459 Å in [BiMe_2_(SbF_6_)]_∞_ and BiMes_2_(SbF_6_)),^21b,37^ suggesting a considerable electronic saturation and/or steric protection of the bismuth center by the six dmso ligands.

**Figure 6:**
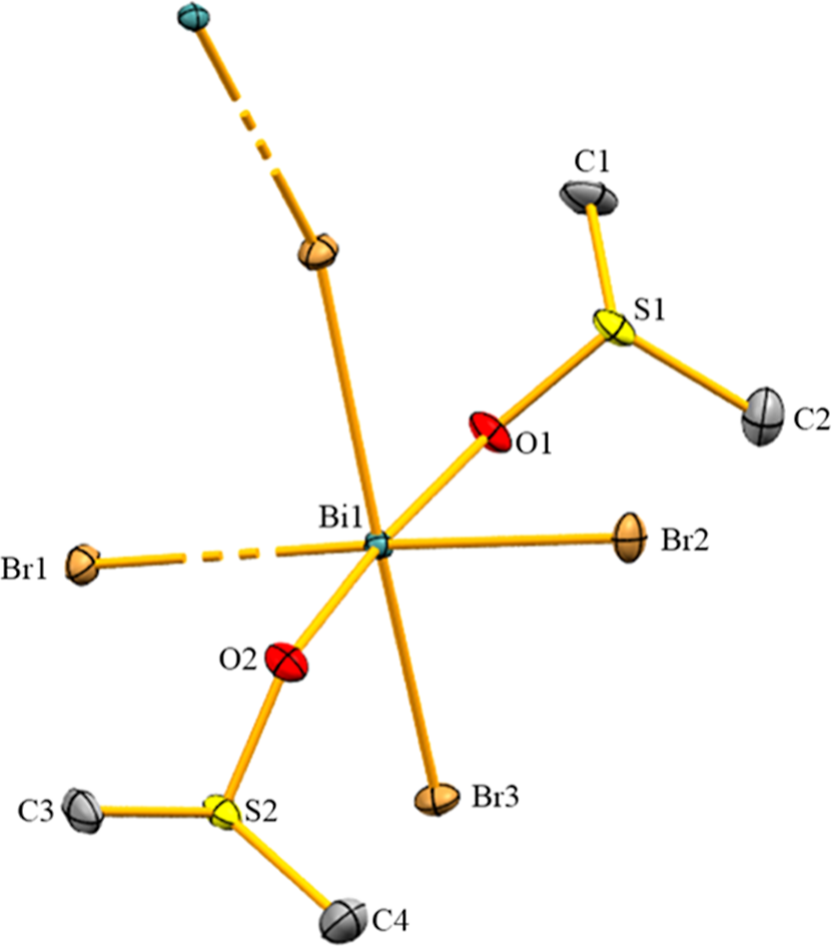
Molecular structure of [BiCl_3_(dmso)_2_]_∞_ in the solid state. Displacement ellipsoids are shown at the 50% probability level. Hydrogen atoms are omitted for clarity.

The structural characterization of [BiBr_3_(dmso)_2_] (monoclinic space group *C*2/*c*, *Z* = 8) revealed the formation of a zigzag-type one-dimensional coordination polymer [BiBr_3_(dmso)_2_]_∞_ in the solid state, which is generated by one bromido ligand per formula unit acting as a μ_2_-bridging ligand (Figure 6). This results in an octahedral coordination geometry around bismuth with the dmso ligands in *trans*-positions. This parallels previous findings on the chlorido derivative, [BiCl_3_(dmso)_2_]_∞_,^38^ but contrasts the behavior of the iodido analog, [BiI_3_(dmso)_2_]_2_, which forms a dimer in the solid state.^36c^

Single-crystal X-ray analysis of compound **4** confirmed its nature as the ion pair [Bi(dmso)_8_][Bi_2_I_9_] with one lattice-bound molecule of acetonitrile (triclinic space group *P*1̅ with *Z* = 2; Figure S5). The same compound without any lattice bound solvent molecules has previously been investigated,^36b,36c,39^ including optical band gap determination.^24^ Analysis of the absorption spectra of solid compound **4** between 200 and 800 nm revealed an optical bandgap of 1.71 eV, as determined from a Tauc plot (Supporting Information). While it is important to note that the values of optical band gaps may differ significantly depending on the method of choice and the nature of sample (e.g., single crystals, powders or thin films),^40^ this value is comparable to, or even lower than those observed for similar compounds such as solvent-free [Bi(dmso)_8_][Bi_2_I_9_] (2.17 eV)^24^ or [CH_3_(NH_3_)_3_][Bi_2_I_9_] (1.94 eV).^41^

## Conclusions

In summary, we report the synthesis and characterization of the first example of a hepta-coordinate monochlorido-bismuth dication [BiCl(dmso)_6_][BF_4_]_2_ (**1**). Notably, the direct reaction of BiCl_3_ with 2 equiv. AgBF_4_ yielded the dinuclear compound [Bi_2_Cl_2_(dmso)_10_][BF_4_]_4_ (**2**), which can be transformed into **1** by transporting the mononuclear dicationic species through the gas phase. DFT calculations reveal the uncommon coordination number of seven with a distorted axis of the coordination polyhedron to be the thermodynamic minimum for model compounds [BiCl(dmso)_*n*_]^2+^ (*n* = 1–7), with only small energy differences between *n* = 6 (distorted), *n* = 6 (nondistorted), and *n* = 7. Weak intramolecular *n*(O) → p(Bi) interactions have been identified as a reason for the distortion observed in the pentagonal bipyramidal coordination geometry. Compound **1** shows a remarkably pronounced Lewis acidity and a considerably hard character according to the HSAB principle, as determined by the (modified) Gutmann–Beckett method. Attempts to isolate the heavier homologues [Bi(dmso)_6_X][BF_4_]_2_ (X = Br, I) gave mixtures of [BiBr_3_(dmso)_2_]_*n*_ and [Bi(dmso)_6_(BF_4_)][BF_4_]_2_ (**3**) (the latter showing an unusual capped-octahedral coordination geometry) or **3** and [Bi(dmso)_8_][Bi_2_I_9_]·CH_3_CN (**4**), likely as a result of disproportionation reactions. We anticipate that the monochlorido-bismuth dication will serve as a valuable precursor in the chemistry of ionic bismuth compounds and that the fundamental insights into its unusual coordination chemistry will inspire the design of related molecular entities for synthesis, catalysis, and materials science.

## Experimental Section

### General Remarks

All air- and moisture-sensitive manipulations were carried out using standard vacuum line Schlenk techniques or in a glovebox containing an atmosphere of purified argon. Solvents were degassed and purified according to standard laboratory procedures. No uncommon hazards are noted. NMR spectra were recorded on Bruker instruments operating at 400 or 500 MHz with respect to ^1^H. ^1^H and ^13^C NMR chemical shifts are reported relative to SiMe_4_ using the residual ^1^H and ^13^C chemical shifts of the solvent as a secondary standard. ^11^B and ^19^F NMR chemical shifts are reported relative to BF_3_·OEt_2_ and CFCl_3_ as external standards. NMR spectra were recorded at ambient temperature (typically 23 °C), if not otherwise noted. IR spectroscopic measurements were conducted on a Bruker Alpha ATRIR spectrometer. Reflectance measurements were acquired with a Cary 5000 Series UV–vis–NIR Spectrophotometer (Agilent Technologies) equipped with a diffuse reflectance accessory Praying Mantis (Harrick Scientific Products) and used in double-beam mode with full slit height. Elemental analyses were performed on a Leco or a Carlo Erba instrument, and the results are given in %. Single crystals suitable for X-ray diffraction analysis were coated with polyisobutylene or perfluorinated polyether oil in a glovebox, transferred to a nylon loop and then to the goniometer of a diffractometer equipped with a molybdenum X-ray tube (Kα λ = 0.71073 Å). The data obtained were integrated with SAINT and a semiempirical absorption correction from equivalents with SADABS was applied. The structure was solved and refined using the Bruker SHELX 2014 software package. All non-hydrogen atoms were refined with anisotropic displacement parameters. All hydrogen atoms were refined isotropically on calculated positions by using a riding model with their U_iso_ values constrained to 1.5 U_eq_ of their pivot atoms for terminal sp^3^ carbon atoms and 1.2 for all other atoms. Crystallographic data have been deposited with the Cambridge Crystallographic Data Centre as supplementary publication numbers 2326075–2326079. These data can be obtained free of charge from The Cambridge Crystallographic Data Centre.

#### Compound [BiCl(dmso)_6_][BF_4_]_2_ (**1**)

PhBiCl_2_ (200 mg, 0.56 mmol) was added to a solution of AgBF_4_ (218 mg, 1.12 mmol) in DMSO (1 mL) at 0 °C. The reaction mixture was allowed to reach room temperature, then stirred at this temperature for 3 h. The reaction mixture was filtered to remove the formed silver chloride. A solution of hydrogen chloride in diethyl ether (0.15 mL, 0.56 mmol, 3.82 M) was added onto the filtrate, then stirred at room temperature for 24 h. The solvent was removed at 50 °C under reduced pressure and the residue was washed with pentane (2 × 2 mL) and Et_2_O (2 × 2 mL) and dried in vacuo. Subsequently, the crude product was sublimed at 150 °C and 10^–3^ mbar onto a coldfinger (−80 °C), yielding pure **1** as a colorless crystalline material. Yield: 350 mg, 0.39 mmol, 70%. ^11^B NMR (128 MHz, DMSO-*d*_6_): δ = −1.29 (s, BF_4_) ppm. ^19^F NMR (300 MHz, DMSO-*d*_6_): δ = −148.25 (s, BF_4_) ppm. ^1^H NMR (300 MHz, CH_3_CN-*d*_3_): δ = 2.69 (s, dmso) ppm. ^13^C NMR (300 MHz, CH_3_CN-*d*_3_): δ = 39.35 (s, dmso) ppm. Elemental analysis (%; a sublimed sample was analyzed): Anal. calcd for C_12_H_36_B_2_BiClF_8_O_6_S_6_ (886.81 g mol^–1^): C, 16.25; H, 4.09; S, 21.69; found: C, 16.40; H, 4.06; S, 21.15.

#### Compound [Bi_2_Cl_2_(dmso)_10_][BF_4_]_4_ (**2**)

AgBF_4_ (247 mg, 1.27 mmol) was added to a solution of BiCl_3_ (200 mg, 0.63 mmol) in DMSO (2 mL) at 0 °C. The reaction mixture was allowed to reach room temperature, then stirred at this temperature for 2 h. The reaction mixture was filtered to get rid of the formed silver chloride. The solvent was removed at 50 °C under reduced pressure and the residue was washed with pentane (2 × 2 mL) and Et_2_O (2 × 2 mL) and dried in vacuo to give **2** as a colorless powder. Yield: 450 mg, 0.28 mmol, 88%. Crystals suitable for XRD analysis were obtained by diffusion of Et_2_O (1 mL) into a solution of **2** (20.0 mg) in acetonitrile (2 mL). ^1^H NMR (300 MHz, CH_3_CN-*d*_3_): δ 2.79 (s, dmso) ppm. ^13^C NMR (300 MHz, CH_3_CN-*d*_3_): δ 39.11 (s, dmso) ppm. ^11^B NMR (128 MHz, DMSO-*d*_6_): δ −1.29 (s, BF_4_) ppm. ^19^F NMR (300 MHz, DMSO-*d*_6_): δ −148.19 (m, BF_4_) ppm. Elemental analysis (%; a sample recrystallized from acetonitrile/Et_2_O was analyzed): Anal. calcd for C_20_H_60_B_4_Bi_2_Cl_2_F_16_O_10_S_10_ (1617.37 g mol^–1^): C, 14.85; H, 3.74; S, 19.82. Found: C, 15.10; H, 3.72; S, 19.77.

#### Preparation of Compound **1** from Compound **2**

The crude product of compound **2** (100 mg, 0.062 mmol) was sublimed at 150 °C and 10^–3^ mbar onto a coldfinger (−80 °C), yielding pure **1** as a colorless crystalline material. Yield: 54 mg, 0.061 mmol, 49%.

#### Reaction of BiBr_3_ with AgBF_4_

AgBF_4_ (87 mg, 0.45 mmol) was added to a solution of BiBr_3_ (100 mg, 0.22 mmol) in DMSO (2 mL) at 0 °C. The reaction mixture was allowed to reach room temperature, then stirred at this temperature for 2 h. The solvent was removed from the deep red solution at 50 °C under reduced pressure and the residue was washed with pentane (2 × 2 mL) and Et_2_O (2 × 2 mL) and dried in vacuo. The resulting precipitate was crystallized by diffusion of Et_2_O (1 mL) into a solution of the solid (60.0 mg) in acetonitrile (3 mL) giving a mixture of [Bi(dmso)_6_(BF_4_)][BF_4_]_2_ (**3**) and [BiBr_3_(dmso)_2_]_∞_ (see main text).

#### Compound [Bi(dmso)_6_(BF_4_)][BF_4_]_2_ (**3**)

AgBF_4_ (130 mg, 0.67 mmol) was added to a solution of BiBr_3_ (100 mg, 0.22 mmol) in DMSO (2 mL) at 0 °C. The reaction mixture was allowed to reach room temperature, then stirred at this temperature for 2 h. The reaction mixture was filtered to get rid of the formed silver bromide. The solvent was removed at 50 °C under reduced pressure and the residue was washed with pentane (2 × 2 mL) and Et_2_O (2 × 2 mL) and dried in vacuo to give [Bi(dmso)_6_(BF_4_)][BF_4_]_2_ (**3**) as a yellow powder. Yield: 192 mg, 0.20 mmol, 92%. Crystals suitable for XRD analysis were obtained by diffusion of Et_2_O (1 mL) into a solution of compound **3** (20.0 mg) in acetonitrile (2 mL). Elemental analysis (%; a sample recrystallized from acetonitrile/Et_2_O was analyzed): Anal. calcd for C_12_H_36_BiB_3_F_12_O_6_S_6_ (938.17 g mol^–1^): C, 15.36; H, 3.87; S, 20.50. Found: C, 15.30; H, 3.81; S, 20.58. ^1^H NMR (300 MHz, CH_3_CN-*d*_3_): δ 2.78 (s, dmso) ppm. ^13^C NMR (300 MHz, CH_3_CN-*d*_3_): δ 38.46 (s, dmso) ppm. ^11^B NMR (128 MHz, DMSO-*d*_6_): δ −1.29 (s, BF_4_) ppm. ^19^F NMR (300 MHz, DMSO-*d*_6_): δ −148.25 (s, BF_4_) ppm.

#### Compound [Bi(dmso)_8_][Bi_2_I_9_]·MeCN (**4**)

AgBF_4_ (66 mg, 0.34 mmol) was added to a solution of BiI_3_ (100 mg, 0.17 mmol) in DMSO (2 mL) at 0 °C. The reaction mixture was allowed to reach room temperature, then stirred at this temperature for 2 h. The solvent was removed from the deep red solution at 50 °C under reduced pressure and the residue was washed with pentane and Et_2_O and dried in vacuo. The resulting precipitate was crystallized by diffusion of Et_2_O (1 mL) into a solution of the solid in acetonitrile (3 mL) giving a mixture of red **4** and **3** that can be easily separated from each other since **4** crystallized first as red crystals which can be isolated by filtration. From the remaining yellow solution, compound **3** was precipitated by adding Et_2_O (2 mL), isolated by filtration, and dried in vacuo. **4**, red crystals, Yield: 36 mg, 0.015 mmol, 79%. Elemental analysis (%; a sample recrystallized from acetonitrile/Et_2_O was analyzed), Anal. calcd for C_18_H_51_Bi_3_I_9_O_8_S_8_N (2435.17 g mol^–1^): C, 8.88; H, 2.12; N, 0.58; S, 10.53. Found: C, 8.98; H, 3.72; N, 0.59; S, 10.64. ^1^H NMR (300 MHz, CH_3_CN-*d*_3_): δ = 2.55 (s, dmso) ppm. ^13^C NMR (300 MHz, CH_3_CN-*d*_3_): δ = 41.18 (s, dmso) ppm. [Bi(dmso)_6_(BF_4_)][BF_4_]_2_ (**3**), yellow crystals, Yield: 88 mg, 0.094 mmol, 83%. Elemental analysis (%; a sample recrystallized from acetonitrile/Et_2_O was analyzed): Anal. calcd for C_12_H_36_BiB_3_F_12_O_6_S_6_ (938.17 g mol^–1^): C, 15.36; H, 3.87; S, 20.50. Found: C, 15.24; H, 3.72; S, 20.64. ^1^H, ^13^C, ^11^B and ^19^F NMR are identical with those that have been reported above for compound **3**.

#### (Modified) Gutmann–Beckett Method^21a,32^

The potential Lewis acid and one equivalent of the Lewis base EPR_3_ per Bi atom (i.e., a 1:1 stoichiometry for **1**/EPR_3_ and a 1:2 stoichiometry for **2**:EPR_3_) were dissolved in acetonitrile-*d*_3_ (E/R = O/Et, S/Me, Se/Me) and analyzed by ^1^H and ^31^P NMR spectroscopy.

### Computational Details

DFT calculations were conducted on [BiCl(dmso)_*n*_]^2+^ (*n* = 0–7) species employing the Gaussian 16, Revision C.01 software package.^42^ These calculations were carried out utilizing the B3LYP^43^ functional and two different basis sets: 6-31G(d,p)^44^ for H, C, O, S, and Cl atoms, and LanL2DZ/ECP^45^ for Bi. Grimme’s D3 dispersion model with the original D3 damping function^46^ was applied to account for dispersion interactions. We opted for this level of theory, which was demonstrated effective in describing similar bismuth-based complexes, to facilitate a direct comparison of our results with previously published work.^1f^ Solvation effects were already considered during geometry optimization and included using the PCM^47^ solvent model with DMSO (ε = 46.826) as the solvent. Our initial tests, which explicitly incorporated counteranions, yielded results that showed no significant differences compared to the analysis of the bare dicationic systems. Consequently, we focused our computational discussion solely on the latter. In our study, we systematically explored various initial geometries and local symmetries for each molecular stoichiometry, ultimately identifying the minimum energy structures by confirming the absence of imaginary frequencies. Gibbs free energies were determined under standard conditions of 298.15 K and 1.00 atm pressure. We incorporated a concentration correction term of Δ*G*^0→*^ = RT ln(24.46) = 1.894 kcal mol^–1^ (*T* = 298.15 K) to adjust the calculated gas-phase values at 1.00 atm to the standard state concentration of 1.00 mol L^–1^. For DMSO, which possesses a standard state concentration of 14.05 mol L^–1^ at 298.15 K, a Δ*G*^0→*^ correction of 3.460 kcal mol^–1^ was applied. This correction procedure enables a better description of associative/dissociative steps,^48^ facilitating improved estimation of free energies related to dmso addition to [BiCl]^2+^. To unravel the underlying factors contributing to the distorted pentagonal bipyramidal coordination geometry observed in [BiCl(dmso)_6_]^2+^, we conducted NBO^49^ calculations on both the optimized structure of [BiCl(dmso)_6_]^2+^ and a model system in which the angle between the Cl atom and the dmso ligand *trans* to it was constrained to 180°. These calculations were executed using the same level of theory previously mentioned. Finally, the characterization of the Bi(p) character of specific molecular orbitals was carried out using orbital composition analysis with the Mulliken partition method, as implemented in Multiwfn 3.8.^50^

## Supporting Information Available

Plots of NMR and IR spectra, crystallographic details, details on DFT computations (PDF). Crystallographic information (CIF) (PDF)
