# Supplementary material for: The Chlorido-Bismuth Dication: A Potent Lewis Acid Captured in a Hepta-Coordinate Species with a Stereochemically Active Lone Pair
Source: Inorg Chem. 2024 Jun 20;63(26):12089–99. doi: 10.1021/acs.inorgchem.4c01076 (PMC11220759; doi:10.1021/acs.inorgchem.4c01076)
Supplement: Supplementary file 1 — ic4c01076_si_001.pdf [file ic4c01076_si_001.pdf]

Supporting Information

for

The Chlorido-Bismuth Dication: A Potent Lewis Acid Captured in a Hepta-Coordinate Species with a Stereochemically Active Lone Pair

Ahmed Fetoh,[a,b] Felipe Fantuzzi,[c,*] and Crispin Lichtenberg[a,*]

a: Department of Chemistry, Philipps-University Marburg Hans-Meerwein-Str. 4, Marburg, 35032, Germany
E-mail: crispin.lichtenberg@chemie.uni-marburg.de

b: Department of Chemistry, Faculty of Science, Mansoura University
El Gomhouria, Mansoura Qism 2, Dakahlia Governorate,11432, Mansoura, Egypt

c: School of Chemistry and Forensic Science, University of Kent
Park Wood Rd, Canterbury CT2 7NH, United Kingdom
E-mail: f.fantuzzi@kent.ac.uk

Contents

Single Crystal X-ray Diffraction Analysis 2
Bond Lengths (Å) and Angles (°) of Isolated Complexes 6
^{11}B and ^{19}F NMR Spectra of Isolated Complexes 10
^{1}H and ^{13}C NMR Spectra of Isolated Complexes 13
Lewis Acidity 18
UV-vis Spectrum and Tauc plot of 4 26
IR Spectroscopy 30
Additional Computational Data 32
Cartesian Coordinates (Ångstrom) 46
References 58

Single Crystal X-ray Diffraction Analysis

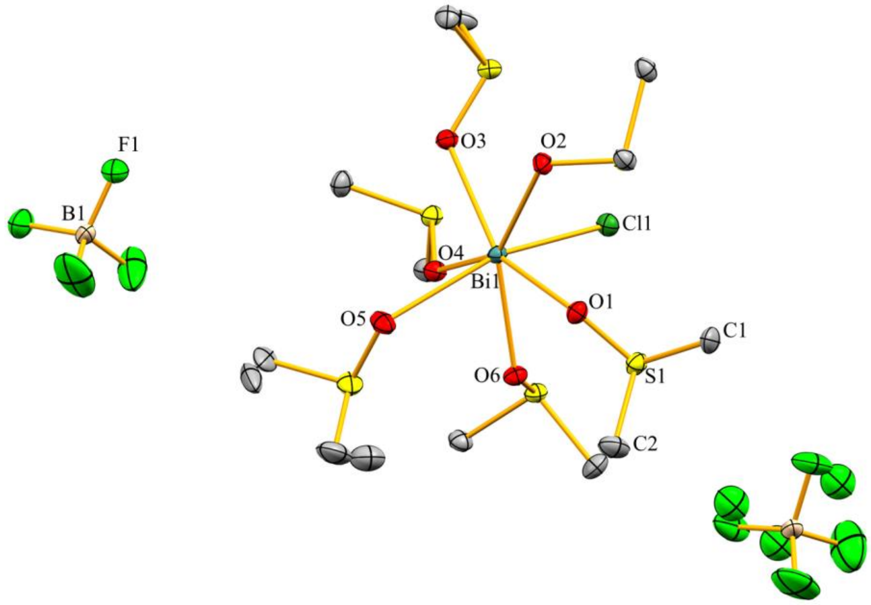

Figure S1. Molecular structure of **1** in the solid state. Displacement ellipsoids are shown at the 50% probability level. Hydrogen atoms are omitted for clarity. Selected bond lengths (Å) and angles (°): Bi1–Cl1, 2.536(1); Bi1–O1, 2.416(3); Bi1–O2, 2.431(3); Bi1–O3, 2.447(3); Bi1–O4, 2.405(3); Bi1–O5, 2.607(4); Bi1–O6, 2.405(3); Cl1–Bi1–O1, 86.79(8); Cl1–Bi1–O2, 84.37(8); Cl1–Bi1–O3, 88.02(8); Cl1–Bi1–O4, 87.51(8); Cl1–Bi1–O5, 159.09(8); Cl1–Bi1–O6, 85.80(8); O4–Bi1–O6, 71.40(11); O4–Bi1–O1, 143.11(11); O6–Bi1–O1, 71.84(11); O4–Bi1–O2, 143.21(11); O6–Bi1–O2, 143.12(11); O1–Bi1–O2, 72.18(11); O4–Bi1–O3, 71.21(10); O6–Bi1–O3, 142.31(10); O1–Bi1–O3, 144.82(11); O2–Bi1–O3, 72.69(10); O4–Bi1–O5, 78.25(11); O6–Bi1–O5, 75.23(11); O1–Bi1–O5, 95.40(11); O2–Bi1–O5, 116.13(11); O3–Bi1–O5, 101.54(11); S5–O5–Bi1, 122.87(19); S6–O6–Bi1, 121.41(17); S4–O4–Bi1, 124.85(17); S3–O3–Bi1, 122.82(17); S2–O2–Bi1, 120.60(17).

The coordination number of bismuth compounds that only bear oxygen-based ligands has been reported to be strongly correlated with the Bi–O bond lengths: octahedral configuration, *d*(Bi-O) ≈ 2.31 Å as in $[Bi(dmpu)_6]^{2+}$;[1] dodecahedral configuration, *d*(Bi-O) ≈ 6 × 2.45 + 2 × 2.37 Å (mean 2.43 Å) as in $[Bi(dmso\text{-}O)_8][Bi_2I_9]$;[2] tricapped trigonal prismatic configuration, *d*(Bi-O) ≈ 6 × 2.45 + 3 × 2.58 Å (mean 2.49 Å) as in $[Bi(H_2O)_9]^{3+}$.[2, 3] The differences in ionic radius between six- and eight-coordinated bismuth ions and between six- and nine-coordinated bismuth ions have been reported by Shannon to be 0.06 and 0.14 Å, respectively.[4] As there are no reports on seven-coordinated bismuth ions that are only coordinated by oxygen-based donors, compound **1** can help to fill this gap in an approximative way, since it shows only monodentate ligands (ruling out weak coordination by hemilabile ligands) and bears six oxygen-based ligands plus one chloride ligand (Bi–O bond length in the equatorial plane: ca. 2.42 Å). The differences in ionic radius between six- and seven-coordinated bismuth ions, between seven- and eight-coordinated bismuth ions, and between seven- and nine-coordinated bismuth ions are found to be 0.11, 0.01 and 0.07 Å, respectively.

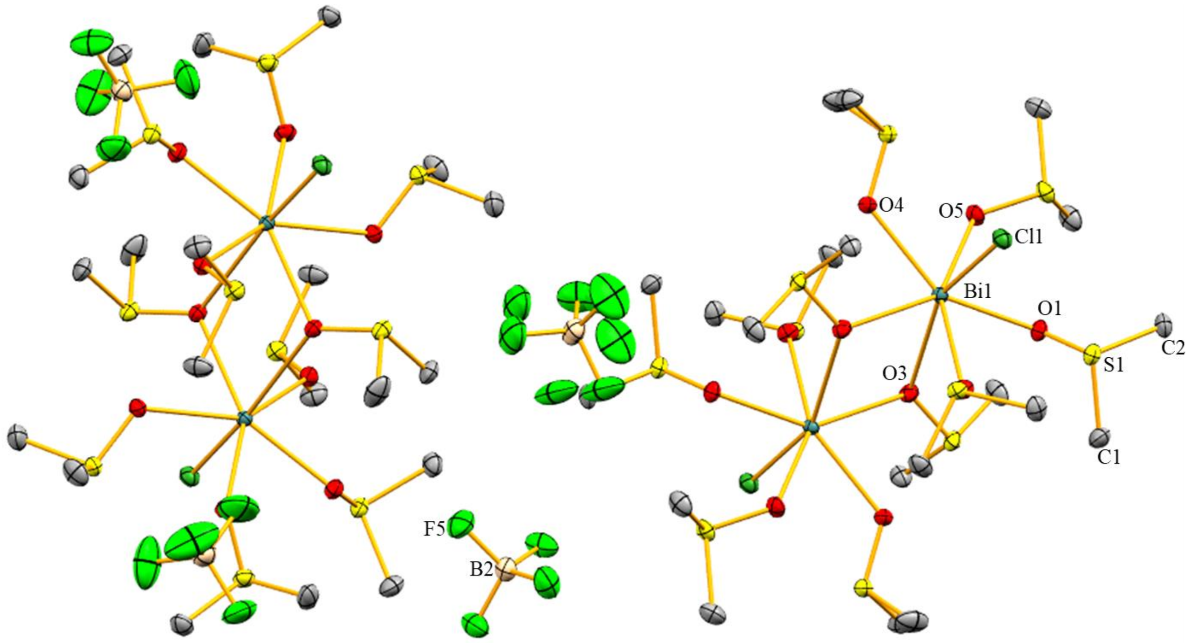

Figure S2. Molecular structure of **2** in the solid state. Displacement ellipsoids are shown at the 50% probability level. Hydrogen atoms are omitted for clarity.

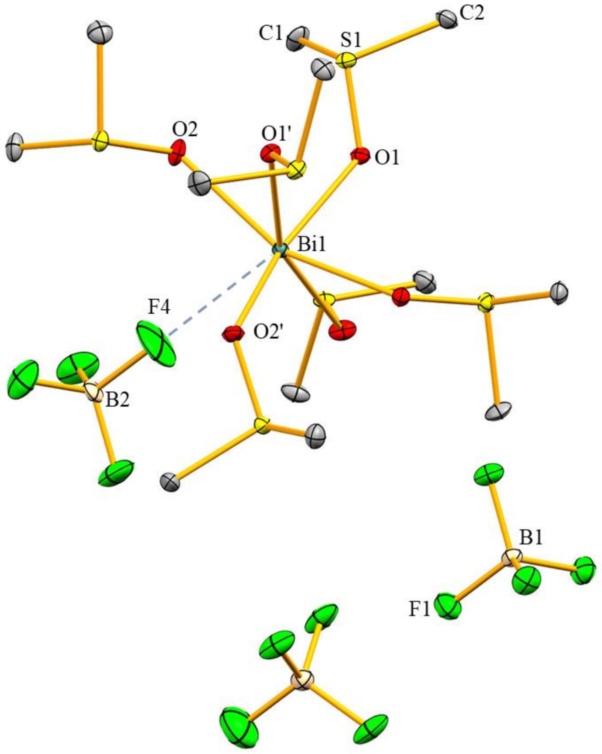

Figure S3. Molecular structure of $[Bi(dmso)_6(BF_4)]][BF_4]_2$ **(3)** in the solid state. Displacement ellipsoids are shown at the 50% probability level. Hydrogen atoms are omitted for clarity.

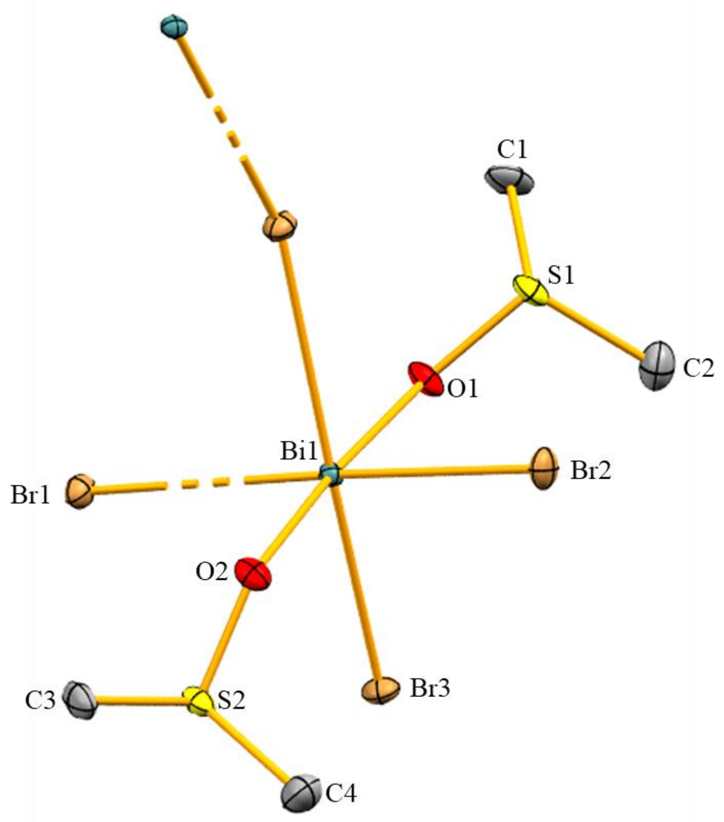

Figure S4. Molecular structure of **[BiBr$_3$(dmso)$_2$]**$_\infty$ in the solid state. Displacement ellipsoids are shown at the 50% probability level. Hydrogen atoms are omitted for clarity.

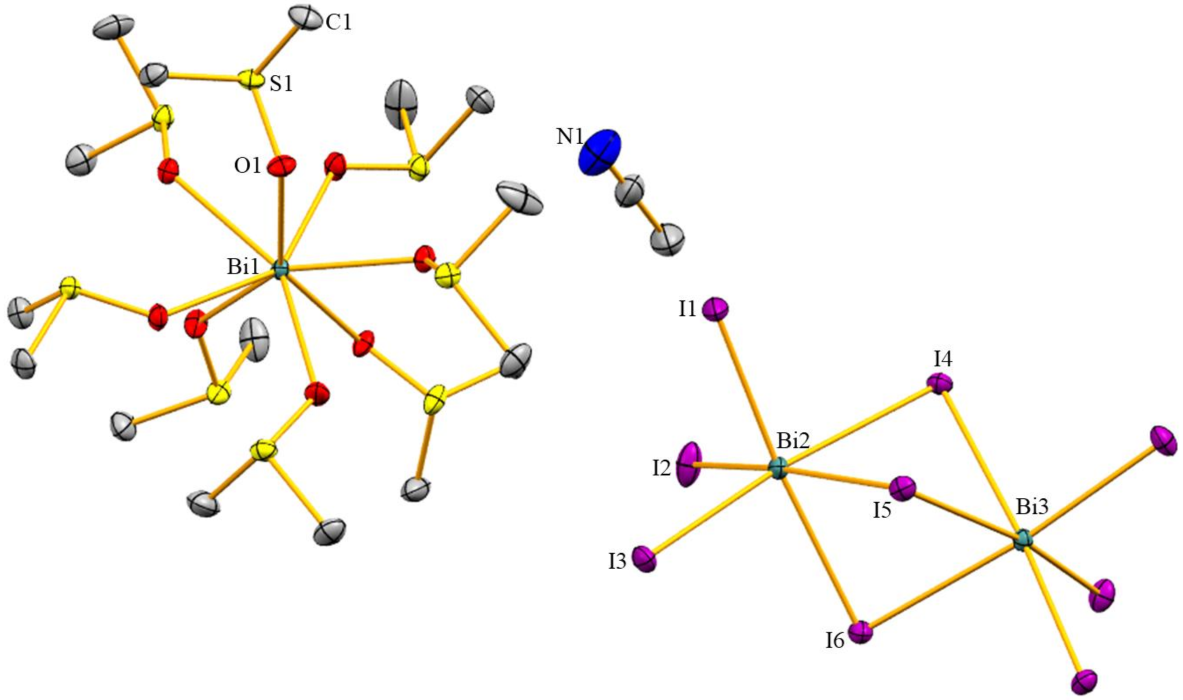

Figure S5. Molecular structure of **4** in the solid state. Displacement ellipsoids are shown at the 50% probability level. Hydrogen atoms are omitted for clarity.

Table S1. Crystallographic Data of **1-Cl**, **2-Cl**, **[Bi(dmso)$_6$(BF$_4$)][BF$_4$]$_2$**, and **[BiBr$_3$(dmso)$_2$]$_\infty$.**

Compound	**1**	**2**	**3**	**[BiBr$_3$(dmso)$_2$]$_\infty$**	**4**
CCDC number	2326079	2326077	2326075	2326076	2326078
Empirical formula	$C_{12}H_{36}B_2BiClF_8O_6S_6$	$C_{20}H_{60}B_4Bi_2Cl_2F_{16}O_{10}S_{10}$	$C_{12}H_{36}B_3BiF_{12}O_6S_6$	$C_4H_{12}BiBr_3O_2S_2$	$C_{18}H_{51}Bi_3I_9NO_8S_8$
Formula weight	886.82	1619.39	938.17	604.97	2435.11
Temperature [K]	100(2)	100(2)	100(2)	100(2)	100(2)
Crystal system	monoclinic	triclinic	trigonal	monoclinic	triclinic
Space group (number)	$P2_1/c$ (14)	$P\bar{1}$ (2)	$R3{:}H$ (146)	$C2/c$ (15)	$P\bar{1}$ (2)
a [Å]	14.012(3)	11.186(5)	11.948(2)	23.555(5)	12.562(3)
b [Å]	19.892(4)	12.435(4)	11.948(2)	8.3813(17)	14.890(3)
c [Å]	11.962(2)	20.821(5)	19.920(4)	14.504(3)	16.453(3)
α [°]	90	87.332(15)	90.00(3)	90	86.093(5)
β [°]	103.686(8)	77.54(2)	90.00(3)	106.622(12)	78.075(7)
γ [°]	90	71.78(2)	120.00(3)	90	73.102(8)
Volume [Å^3]	3239.3(11)	2685.6(17)	2462.5(10)	2743.9(10)	2880.9(10)
Z	4	2	3	8	2
ρ_{calc} [gcm^{-3}]	1.818	2.003	1.898	2.929	2.807
μ [mm^{-1}]	5.983	7.128	5.846	21.862	14.283
F(000)	1736	1572	1374	2176	2168
Crystal size [mm^3]	0.346×0.279×0.172	0.145×0.133×0.123	0.231×0.165×0.113	0.305×0.297×0.160	0.147×0.134×0.127
Crystal colour	colorless	colorless	yellow	colorless	red
Crystal shape	block	block	block	block	block
Radiation	MoK_α (λ=0.71073 Å)	MoK_α (λ=0.71073 Å)	MoK_α (λ=0.71073 Å)	MoK_α (λ=0.71073 Å)	MoK_α (λ=0.71073 Å)
2θ range [°]	4.06 to 52.74 (0.80 Å)	4.35 to 67.36 (0.64 Å)	4.44 to 66.83 (0.65 Å)	5.18 to 67.30 (0.64 Å)	4.67 to 67.32 (0.64 Å)
Index ranges	−17 ≤ h ≤ 17<br>−24 ≤ k ≤ 24<br>−14 ≤ l ≤ 14	−16 ≤ h ≤ 17<br>−17 ≤ k ≤ 17<br>−32 ≤ l ≤ 32	−18 ≤ h ≤ 18<br>−16 ≤ k ≤ 17<br>−30 ≤ l ≤ 29	−36 ≤ h ≤ 33<br>−12 ≤ k ≤ 12<br>−22 ≤ l ≤ 19	−19 ≤ h ≤ 19<br>−21 ≤ k ≤ 22<br>−25 ≤ l ≤ 23
Reflections collected	61788	110250	11404	49822	122521
Independent reflections	6620<br>R_{int} = 0.0314<br>R_{sigma} = 0.0183	18316<br>R_{int} = 0.0361<br>R_{sigma} = 0.0308	3367<br>R_{int} = 0.0539<br>R_{sigma} = 0.0526	4988<br>R_{int} = 0.0690<br>R_{sigma} = 0.0379	19629<br>R_{int} = 0.0462<br>R_{sigma} = 0.0385
Completeness to θ = 25.242°	99.9 %	99.9 %	100.0 %	100.0 %	99.9 %
Data / Restraints / Parameters	6620/1/374	18316/30/619	3367/152/126	4988/0/113	19629/0/441
Goodness-of-fit on F^2	1.075	1.046	1.004	1.023	1.032
Final *R* indexes<br>[I≥2σ(I)]	R_1 = 0.0299<br>wR_2 = 0.0680	R_1 = 0.0244<br>wR_2 = 0.0437	R_1 = 0.0241<br>wR_2 = 0.0344	R_1 = 0.0273<br>wR_2 = 0.0555	R_1 = 0.0271<br>wR_2 = 0.0448
Final *R* indexes<br>[all data]	R_1 = 0.0340<br>wR_2 = 0.0697	R_1 = 0.0352<br>wR_2 = 0.0468	R_1 = 0.0254<br>wR_2 = 0.0346	R_1 = 0.0336<br>wR_2 = 0.0573	R_1 = 0.0401<br>wR_2 = 0.0475
Largest peak/hole [eÅ^{-3}]	1.93/-0.61	1.38/-1.11	0.81/-0.74	2.01/-2.53	1.10/-1.58
Completeness to θ = 25.242°	99.9 %	99.9 %	100.0 %	100.0 %	99.9 %
Data / Restraints / Parameters	6620/1/374	18316/30/619	3367/152/126	4988 / 0 / 113	19629/0/441
Goodness-of-fit on F^2	1.075	1.046	1.004	1.023	1.032
Final *R* indexes [I≥2σ(I)]	R_1 = 0.0299, wR_2 = 0.0680	R_1 = 0.0244, wR_2 = 0.0437	R_1 = 0.0241, wR_2 = 0.0344	R1 = 0.0273, wR2 = 0.0555	R_1 = 0.0271, wR_2 = 0.0448
Final *R* indexes [all data]	R_1 = 0.0340, wR_2 = 0.0697	R_1 = 0.0352, wR_2 = 0.0468	R_1 = 0.0254, wR_2 = 0.0346	R1 = 0.0336, wR2 = 0.0573	R_1 = 0.0401, wR_2 = 0.0475
Largest peak/hole [eÅ^{-3}]	1.93/-0.61	1.38/-1.11	0.81/-0.74	2.007/-2.529	1.10/-1.58

Bond Lengths (Å) and Angles (°) of Isolated Complexes

Table S2. Bond lengths (Å) of **1**.

Atom–Atom	Length [Å]	Atom–Atom	Length [Å]	Atom–Atom	Length [Å]
F8–B2	1.351(7)	B2–F7	1.339(9)	C7–S4	1.785(5)
C8–S4	1.773(5)	B2–F6	1.414(8)	O4–S4	1.533(3)
S6–O6	1.538(3)	B2–F5A	1.469(19)	S5–C9	1.67(2)
S6–C12	1.775(5)	S3–O3	1.537(3)	S5–C10A	1.681(18)
S6–C11	1.793(5)	S3–C5	1.780(5)	S5–C10	1.85(2)
B1–F2	1.345(7)	S3–C6	1.783(5)	S5–C9A	1.868(16)
B1–F3	1.360(7)	O5–S5	1.524(3)	Bi1–O6	2.405(3)
B1–F4	1.369(6)	S1–O1	1.530(3)	Bi1–O1	2.415(3)
B1–F1	1.372(6)	S1–C2	1.780(5)	Bi1–O2	2.431(3)
S2–O2	1.541(3)	S1–C1	1.785(5)	Bi1–O3	2.447(3)
S2–C4	1.770(5)	B2–F7A	1.25(2)	Bi1–Cl1	2.5358(12)
S2–C3	1.786(5)	B2–F6A	1.29(2)	Bi1–O5	2.606(3)
Bi1–O4	2.405(3)	B2–F5	1.295(9)		

Table S3. Bond angles (°) of **1**.

	Angle [°]		Angle [°]		Angle [°]
S6–O6–Bi1	121.41(17)	O1–Bi1–O2	72.18(11)	O1–S1–C1	104.7(2)
S4–O4–Bi1	124.85(17)	O4–Bi1–O3	71.21(10)	C2–S1–C1	98.4(3)
S1–O1–Bi1	122.82(17)	O6–Bi1–O3	142.31(10)	F7A–B2–F6A	108.9(16)
C8–S4–C7	99.1(3)	O1–Bi1–O3	144.82(11)	F5–B2–F7	115.1(10)
S3–O3–Bi1	122.82(17)	O2–Bi1–O3	72.69(10)	F7A–B2–F8	119.9(10)
O4–S4–C7	105.0(2)	O4–Bi1–Cl1	87.51(8)	F6A–B2–F8	97.7(9)
F2–B1–F3	108.4(6)	O6–Bi1–Cl1	85.80(8)	F5–B2–F8	113.7(7)
F2–B1–F4	112.0(6)	O1–Bi1–Cl1	86.79(8)	F7–B2–F8	99.4(6)
F3–B1–F4	108.9(5)	O2–Bi1–Cl1	84.37(8)	F5–B2–F6	112.5(6)
F2–B1–F1	108.8(5)	O3–Bi1–Cl1	88.02(8)	F7–B2–F6	103.0(6)
F3–B1–F1	107.8(6)	O4–Bi1–O5	78.25(11)	F8–B2–F6	111.9(6)
F4–B1–F1	110.7(4)	O6–Bi1–O5	75.23(11)	F7A–B2–F5A	104.9(14)
O2–S2–C4	103.6(2)	O1–Bi1–O5	95.40(11)	F6A–B2–F5A	100.9(13)
O2–S2–C3	104.2(2)	O2–Bi1–O5	116.13(11)	F8–B2–F5A	121.8(9)
C4–S2–C3	99.6(2)	O3–Bi1–O5	101.54(11)	O3–S3–C5	105.1(2)
O4–Bi1–O6	71.40(11)	Cl1–Bi1–O5	159.09(8)	O3–S3–C6	104.0(2)
O4–Bi1–O1	143.11(11)	O1–S1–C2	103.7(2)	C5–S3–C6	98.6(3)
O6–Bi1–O1	71.84(11)	O6–S6–C12	102.8(2)	S5–O5–Bi1	122.87(19)
O4–Bi1–O2	143.21(11)	O6–S6–C11	105.0(2)	O4–S4–C8	103.2(2)
O6–Bi1–O2	143.12(10)	C12–S6–C11	98.0(2)	C10A–S5–C9A	98.5(9)
O5–S5–C9	105.4(6)	O5–S5–C10A	105.3(7)	S2–O2–Bi1	120.60(17)
O5–S5–C10	103.0(8)	C9–S5–C10	99.3(8)	O5–S5–C9A	105.6(6)

Table S4. Bond lengths (Å) of **2**.

Atom–Atom	Length [Å]	Atom–Atom	Length [Å]	Atom–Atom	Length [Å]
Bi1–O1	2.325(2)	S1–O1	1.5631(19)	Bi2–O7	2.3669(18)
Bi1–O5	2.3472(18)	S1–C2	1.778(3)	Bi2–O6	2.3923(17)
Bi1–O2	2.385(3)	C20–S10	1.780(3)	Bi2–O10	2.4113(18)
Bi1–O4	2.4197(17)	S2–O2	1.554(2)	Bi2–O8	2.4201(17)
Bi1–Cl1	2.4977(13)	S2–C3	1.774(3)	Bi2–Cl2	2.4712(13)
Bi1–O3	2.6626(18)	S2–C4	1.785(3)	Bi2–O9	2.4990(17)
Bi1–O3'	2.758(2)	S3–O3	1.5429(17)	C19–S10	1.776(3)
C1–S1	1.777(3)	S3–C6	1.781(3)	S4–O4	1.5308(18)
O10–S10	1.5410(18)	S3–C5	1.782(3)	S4–C8	1.777(3)
B2–F8	1.384(3)	B3–F10	1.356(4)	S4–C7	1.778(3)
B2–F5	1.390(3)	B3–F9	1.356(4)	B4–F15	1.366(4)

B2–F6	1.394(3)	B3–F12	1.380(4)	B4–F13	1.375(4)
B2–F7	1.396(3)	B3–F11	1.381(4)	B4–F14	1.380(4)
B1–F4	1.364(8)	S6–O6	1.5385(19)	B4–F16	1.383(4)
B1–F2	1.375(8)	S6–C11	1.775(3)	S5–O5	1.5424(18)
B1–F1	1.385(8)	S6–C12	1.787(3)	S5–C10	1.777(3)
B1–F3	1.393(8)	O7–S7	1.5381(18)	S5–C9	1.778(3)
B1A–F4A	1.349(13)	S7–C13	1.779(3)	S8–O8	1.5364(18)
B1A–F2A	1.364(13)	S7–C14	1.784(3)	S8–C15	1.780(3)
B1A–F1A	1.369(12)	O9–S9	1.5458(18)	S8–C16	1.782(3)
B1A–F3A	1.376(12)	S9–C17	1.778(3)		

Table S5. Bond angles (°) of **2**.

	**Angle [°]**		**Angle [°]**		**Angle [°]**
O1–Bi1–O5	74.36(7)	O1–S1–C1	104.37(13)	S1–O1–Bi1	121.29(12)
O1–Bi1–O2	74.88(8)	O1–S1–C2	102.46(13)	O7–Bi2–O6	73.49(6)
O5–Bi1–O2	148.71(8)	C1–S1–C2	99.85(13)	O7–Bi2–O10	144.92(6)
O1–Bi1–O4	146.66(7)	O2–S2–C3	103.33(14)	O6–Bi2–O10	72.20(6)
O5–Bi1–O4	73.74(6)	O2–S2–C4	104.67(14)	O7–Bi2–O8	72.48(6)
O2–Bi1–O4	134.88(7)	C3–S2–C4	98.75(13)	O6–Bi2–O8	145.27(6)
O1–Bi1–Cl1	86.08(6)	S2–O2–Bi1	121.30(13)	O10–Bi2–O8	139.76(6)
O5–Bi1–Cl1	87.81(5)	O3–S3–C6	104.73(11)	O7–Bi2–Cl2	88.78(5)
O2–Bi1–Cl1	84.51(6)	O3–S3–C5	104.95(12)	O6–Bi2–Cl2	88.32(5)
O4–Bi1–Cl1	83.26(5)	C6–S3–C5	96.59(13)	O10–Bi2–Cl2	83.16(5)
O1–Bi1–O3	142.49(7)	S3–O3–Bi1	124.37(9)	O8–Bi2–Cl2	84.25(5)
O5–Bi1–O3	143.04(6)	S3–O3–Bi1	130.79(9)	O7–Bi2–O9	142.59(6)
O2–Bi1–O3	68.10(8)	Bi1–O3–Bi1	90.51(6)	O6–Bi2–O9	143.91(6)
O4–Bi1–O3	70.28(5)	F10–B3–F9	108.7(3)	O10–Bi2–O9	72.07(6)
Cl1–Bi1–O3	96.05(5)	F10–B3–F12	108.7(3)	O8–Bi2–O9	70.45(6)
O1–Bi1–O3'	75.95(7)	F9–B3–F12	110.4(3)	Cl2–Bi2–O9	92.35(5)
O5–Bi1–O3'	101.34(6)	F10–B3–F11	112.8(3)	S5–O5–Bi1	123.98(10)
O2–Bi1–O3'	76.49(6)	F9–B3–F11	107.2(3)	O6–S6–C11	105.03(12)
O4–Bi1–O3'	119.89(6)	F12–B3–F11	109.1(3)	O6–S6–C12	102.86(12)
Cl1–Bi1–O3'	156.60(4)	O4–S4–C8	104.78(13)	C11–S6–C12	98.90(13)
O3–Bi1–O3'	89.49(6)	O4–S4–C7	103.65(12)	S6–O6–Bi2	122.31(10)
S8–O8–Bi2	120.82(9)	C8–S4–C7	98.97(16)	S7–O7–Bi2	124.87(10)
S9–O9–Bi2	119.14(9)	S4–O4–Bi1	124.61(9)	O7–S7–C13	103.87(13)
O9–S9–C17	104.81(12)	F15–B4–F13	110.7(3)	O7–S7–C14	103.91(12)
O9–S9–C18	103.95(13)	F15–B4–F14	109.8(2)	C13–S7–C14	99.30(13)
C17–S9–C18	98.34(14)	F13–B4–F14	107.6(3)	O8–S8–C15	104.80(12)
S10–O10–Bi2	120.36(9)	F15–B4–F16	108.7(3)	O8–S8–C16	103.89(12)
O10–S10–C19	104.31(13)	F13–B4–F16	108.3(3)	C15–S8–C16	98.98(15)
O10–S10–C20	104.86(12)	F14–B4–F16	111.7(3)	F8–B2–F5	109.7(2)
C19–S10–C20	99.12(14)	O5–S5–C10	103.77(12)	F8–B2–F6	109.6(2)
F4A–B1A–F2A	109.4(11)	O5–S5–C9	103.87(12)	F5–B2–F6	109.6(2)
F4A–B1A–F1A	113.3(12)	C10–S5–C9	98.81(14)	F8–B2–F7	109.8(2)
F2A–B1A–F1A	107.4(10)	F2A–B1A–F3A	108.6(12)	F5–B2–F7	108.9(2)
F4A–B1A–F3A	108.5(11)	F1A–B1A–F3A	109.5(12)	F6–B2–F7	109.1(2)
F1–B1–F3	111.9(6)	F2–B1–F1	111.3(7)	F4–B1–F2	106.8(6)
F2–B1–F3	110.4(7)	F4–B1–F3	108.8(7)	F4–B1–F1	107.5(6)

Table S6. Bond lengths (Å) of **3**.

**Atom–Atom**	**Length [Å]**	**Atom–Atom**	**Length [Å]**	**Atom–Atom**	**Length [Å]**
Bi1–O1'1	2.302(3)	S1–O1	1.549(3)	B3–F5	1.370(9)
Bi1–O1'2	2.302(3)	S1–C2	1.776(4)	B3–F6'5	1.372(5)
Bi1–O1'3	2.302(3)	F1–B1	1.392(10)	B3–F6'6	1.372(5)
Bi1–O2'1	2.413(3)	B1–F2'3	1.389(4)	B3–F6	1.372(5)
Bi1–O2'2	2.413(3)	B1–F2'4	1.389(4)	B2–F3'1	1.369(4)
Bi1–O2'3	2.413(3)	B1–F2	1.389(4)	B2–F3	1.369(4)
Bi1–F4	2.695(5)	O2–S2	1.544(3)	B2–F3'2	1.369(4)

C1–S1	1.789(4)	B2–F4	1.366(12)	S2–C3	1.777(4)
S2–C4	1.779(4)				

Table S7. Bond angles (°) of **3**.

	**Angle [°]**		**Angle [°]**		**Angle [°]**
O1–Bi1–O1'	87.97(10)	O1–Bi1–O1'	87.97(10)	O1–S1–C2	103.39(18)
O1–Bi1–F4	126.69(7)	O1–Bi1–O2$'^{3}$	78.11(11)	O1–S1–C1	104.23(19)
O2–S2–C3	104.00(19)	O1–Bi1–O2$'^{1}$	158.64(9)	C2–S1–C1	99.2(2)
O2–S2–C4	103.68(18)	O1–Bi1–O2$'^{2}$	75.55(11)	S1–O1–Bi1	124.23(15)
C3–S2–C4	100.4(2)	F6–B3–F6	109.9(5)	F2–B1–F2	110.0(3)
F5–B3–F6	109.1(5)	F5–B3–F6	109.1(5)	S2–O2–Bi1	116.02(15)
O2–Bi1–O2'	113.22(6)	B2–F4–Bi1	180.0	F4–B2–F3	109.0(5)
O1–Bi1–O2	158.64(9)	O2–Bi1–F4	74.61(7)	F2–B1–F1	108.9(3)
O1–Bi1–O2'	75.55(11)	F3–B2–F3	110.0(4)		

Table S8. Bond lengths (Å) of **[BiBr$_3$(dmso)$_2$]**$_\infty$.

**Atom–Atom**	**Length [Å]**	**Atom–Atom**	**Length [Å]**	**Atom–Atom**	**Length [Å]**
Bi1–O2	2.369(3)	S2–O2	1.543(3)	O1–S1	1.547(3)
Bi1–O1	2.392(3)	S2–C4	1.769(4)	S1–C2	1.773(4)
Bi1–Br2	2.6958(5)	S2–C3	1.773(4)	S1–C1	1.781(4)
Bi1–Br3	2.7149(5)	Bi1–Br1	3.0104(6)	Bi1–Br1$'^{1}$	3.0477(6)

Table S9. Bond angles (°) of **[BiBr$_3$(dmso)$_2$]**$_\infty$.

	**Angle [°]**		**Angle [°]**		**Angle [°]**
O2–Bi1–O1	175.55(9)	O2–S2–C4	104.02(19)	Br3–Bi1–Br1	88.03(2)
O2–Bi1–Br2	87.17(7)	O2–S2–C3	103.75(17)	Br1–Bi1–Br1	91.016(17)
O1–Bi1–Br2	88.73(6)	C4–S2–C3	98.9(2)	S1–O1–Bi1	120.55(14)
O2–Bi1–Br3	89.91(7)	S2–O2–Bi1	121.03(14)	Bi1–Br1–Bi1	159.891(15)
O1–Bi1–Br3	88.55(7)	O2–Bi1–Br1	86.92(7)	O1–S1–C2	103.97(19)
Br2–Bi1–Br3	92.68(2)	O1–Bi1–Br1	97.19(6)	O1–S1–C1	103.83(17)
O2–Bi1–Br1	94.54(7)	Br2–Bi1–Br1	174.047(11)	C2–S1–C1	99.2(2)
O1–Bi1–Br1	87.10(7)	Br3–Bi1–Br1	175.391(11)	Br2–Bi1–Br1	88.73(2)

Table S10. Bond lengths (Å) of **4**.

**Atom–Atom**	**Length [Å]**	**Atom–Atom**	**Length [Å]**	**Atom–Atom**	**Length [Å]**
Bi1–O8	2.342(2)	I1–Bi2	2.9912(6)		
Bi1–O6	2.399(2)	Bi2–I3	2.9612(6)	S6–O6	1.541(2)
Bi1–O4	2.432(2)	Bi2–I2	2.9678(5)	S6–C12	1.773(4)
Bi1–O5	2.471(2)	Bi2–I4	3.1779(7)	S6–C11	1.774(4)
Bi1–O3	2.489(2)	Bi2–I5	3.2492(5)	S8–O8	1.538(2)
Bi1–O7	2.489(2)	Bi2–I6	3.2839(7)	S8–C15	1.771(4)
Bi1–O1	2.492(2)	S2–O2	1.532(2)	S8–C16	1.780(4)
Bi1–O2	2.515(2)	S2–C4	1.783(4)	S3–O3	1.529(2)
C1–S1	1.784(4)	S2–C3	1.788(4)	S3–C6	1.780(4)
S1–O1	1.534(2)	Bi3–I7	2.9554(7)	S3–C5	1.790(4)
S1–C2	1.790(4)	Bi3–I8	2.9681(5)	C18–C17	1.460(6)
N1–C17	1.123(6)	Bi3–I9	2.9718(5)	S5–O5	1.532(3)
S4–O4	1.535(2)	Bi3–I6	3.2386(5)	S5–C9	1.771(4)
S4–C7	1.782(4)	Bi3–I5	3.2462(5)	S5–C10	1.784(4)
S4–C8	1.788(4)	Bi3–I4	3.2540(8)	S7–O7	1.529(2)
S7–C14	1.782(4)	S7–C13	1.789(4)		

Table S11. Bond angles (°) of **4**.

	**Angle [°]**		**Angle [°]**		**Angle [°]**
O8–Bi1–O6	77.33(8)	O3–Bi1–O7	130.69(7)	O1–S1–C1	105.01(16)
O8–Bi1–O4	94.00(9)	O8–Bi1–O1	85.61(8)	O1–S1–C2	105.03(16)
O6–Bi1–O4	70.50(8)	O6–Bi1–O1	144.61(8)	C1–S1–C2	98.09(18)
O8–Bi1–O5	142.44(8)	O4–Bi1–O1	142.33(8)	S1–O1–Bi1	120.60(12)
O6–Bi1–O5	75.99(8)	O5–Bi1–O1	101.49(8)	I3–Bi2–I2	90.675(17)
O4–Bi1–O5	101.41(9)	O3–Bi1–O1	82.26(8)	I3–Bi2–I1	92.057(11)
O8–Bi1–O3	145.44(8)	O7–Bi1–O1	74.41(8)	I2–Bi2–I1	95.928(12)
O6–Bi1–O3	127.94(8)	O8–Bi1–O2	72.26(8)	I3–Bi2–I4	91.251(10)
O4–Bi1–O3	76.95(8)	O6–Bi1–O2	127.40(7)	I2–Bi2–I4	87.675(12)
O5–Bi1–O3	71.95(8)	O4–Bi1–O2	70.01(8)	I1–Bi2–I4	175.077(8)
O8–Bi1–O7	75.64(8)	O5–Bi1–O2	145.25(8)	I3–Bi2–I5	95.054(16)
O6–Bi1–O7	71.44(8)	O3–Bi1–O2	73.30(8)	I2–Bi2–I5	169.420(8)
O4–Bi1–O7	141.88(8)	O7–Bi1–O2	136.15(8)	I1–Bi2–I5	92.754(12)
O5–Bi1–O7	71.10(8)	O1–Bi1–O2	74.09(7)	I4–Bi2–I5	83.322(11)
O2–S2–C4	105.29(16)	O3–S3–C6	103.31(16)	I3–Bi2–I6	173.951(8)
O2–S2–C3	104.87(16)	O3–S3–C5	107.00(16)	I2–Bi2–I6	92.373(16)
C4–S2–C3	98.03(19)	C6–S3–C5	97.81(18)	I1–Bi2–I6	92.823(12)
S2–O2–Bi1	125.80(13)	S3–O3–Bi1	134.88(13)	I4–Bi2–I6	83.657(10)
I7–Bi3–I8	93.387(12)	Bi2–I4–Bi3	81.504(10)	I5–Bi2–I6	81.146(16)
I7–Bi3–I9	91.417(11)	O4–S4–C7	103.49(16)	I9–Bi3–I6	173.399(8)
I8–Bi3–I9	92.474(15)	O4–S4–C8	103.82(18)	I7–Bi3–I5	97.289(13)
I7–Bi3–I6	90.722(11)	C7–S4–C8	99.09(19)	I8–Bi3–I5	168.453(8)
I8–Bi3–I6	93.633(15)	S4–O4–Bi1	126.47(13)	I9–Bi3–I5	91.653(14)
O5–S5–C9	105.14(18)	Bi3–I5–Bi2	80.550(13)	I6–Bi3–I5	81.881(14)
O5–S5–C10	104.08(17)	O7–S7–C14	104.43(18)	I7–Bi3–I4	173.902(8)
C9–S5–C10	97.51(19)	O7–S7–C13	105.92(16)	I8–Bi3–I4	86.727(12)
S5–O5–Bi1	129.66(14)	C14–S7–C13	98.7(2)	I9–Bi3–I4	94.669(12)
O6–S6–C12	103.94(17)	S7–O7–Bi1	120.49(13)	I6–Bi3–I4	83.187(12)
O6–S6–C11	103.87(16)	Bi3–I6–Bi2	80.144(10)	I5–Bi3–I4	82.190(13)
C12–S6–C11	98.0(2)	O8–S8–C15	105.53(17)	N1–C17–C18	178.7(5)
S6–O6–Bi1	126.67(13)	O8–S8–C16	102.11(18)	S8–O8–Bi1	126.58(14)
C15–S8–C16	98.4(2)				

^{11}B and ^{19}F NMR Spectra of Isolated Complexes

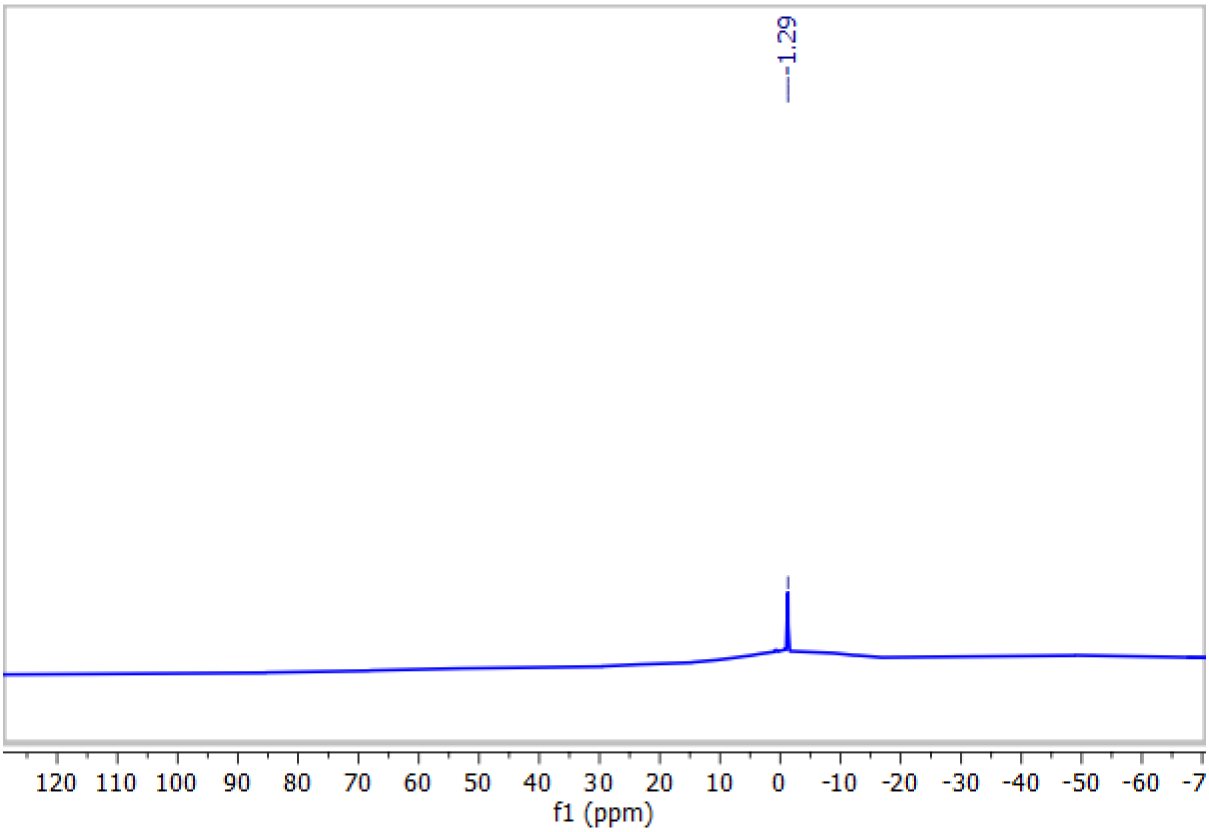

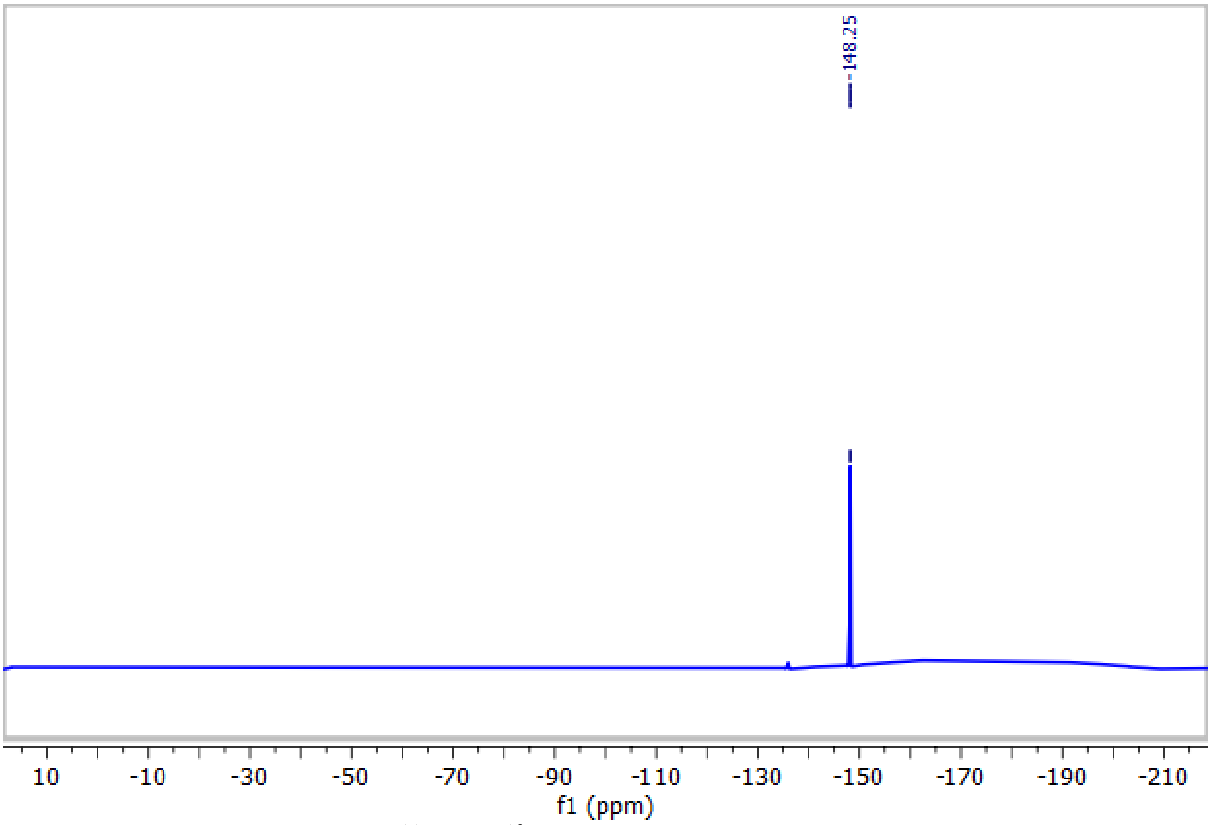

Figure S6. ^{11}B and ^{19}F NMR spectra of **1** in DMSO-d_6.

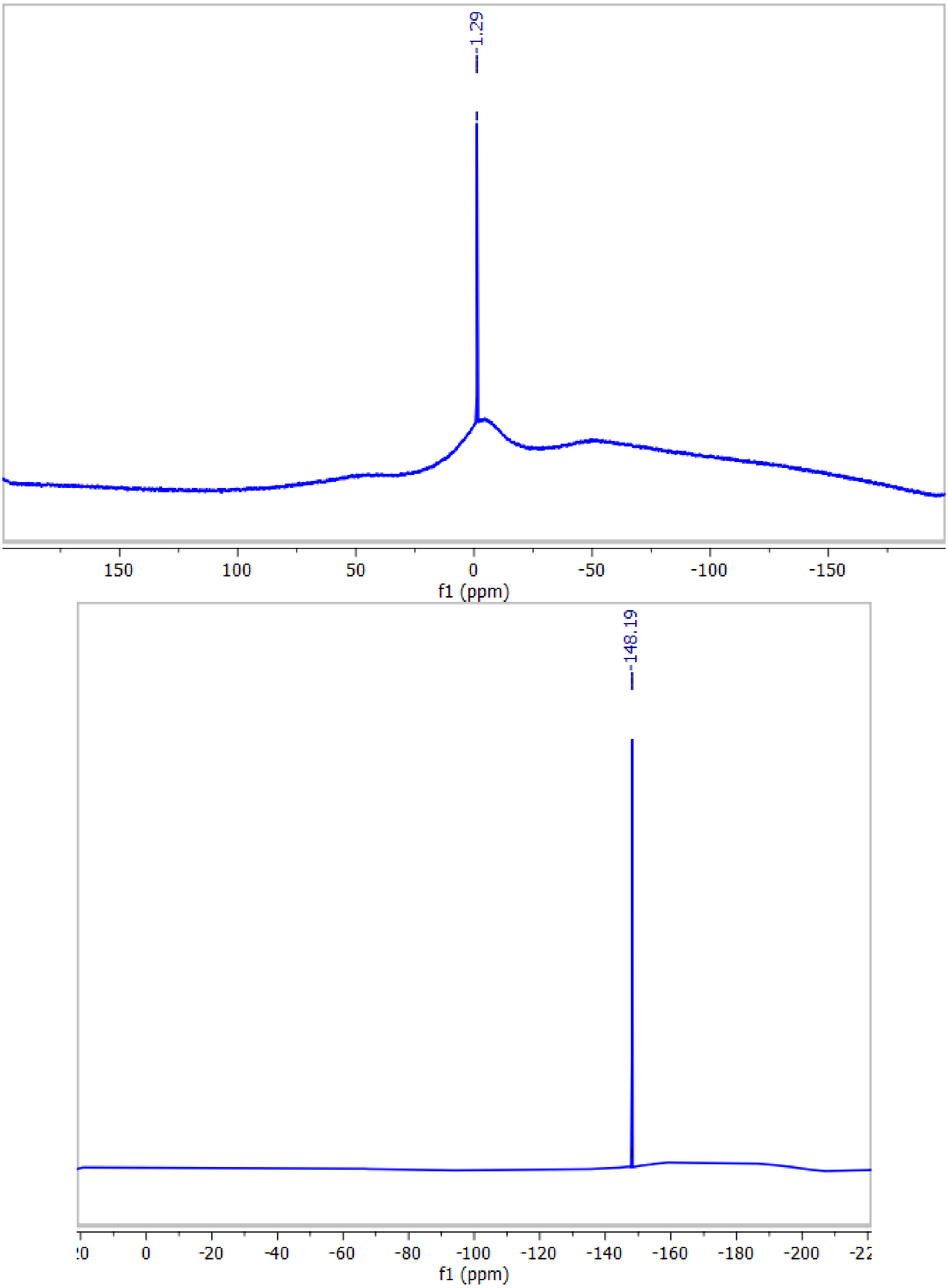

Figure S7. ${}^{11}B$ and ${}^{19}F$ NMR spectra of **2** in DMSO-d_6.

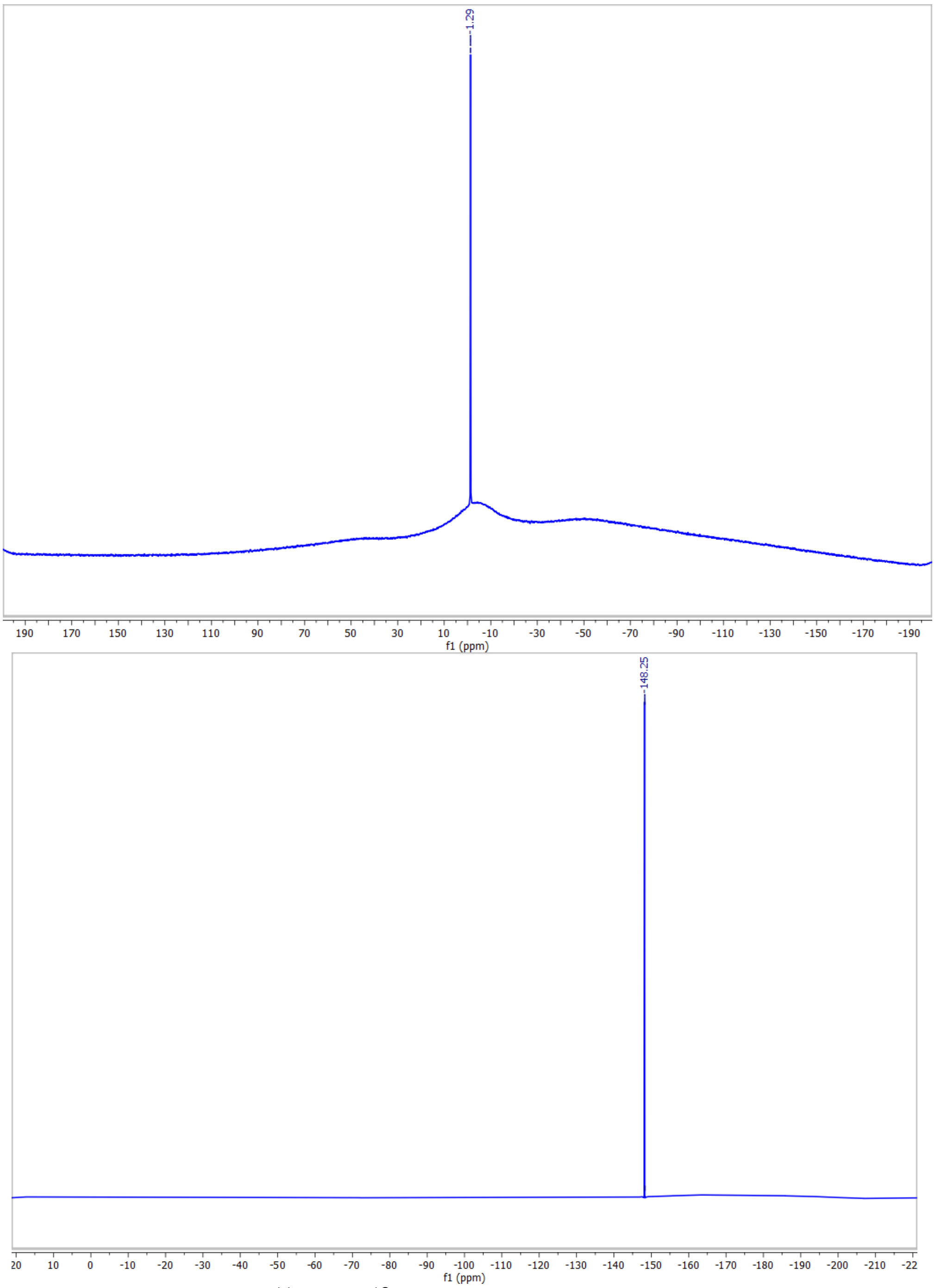

Figure S8. ${}^{11}B$ and ${}^{19}F$ NMR spectra of **3** in DMSO-d_6.

^{1}H and ^{13}C NMR Spectra of Isolated Complexes

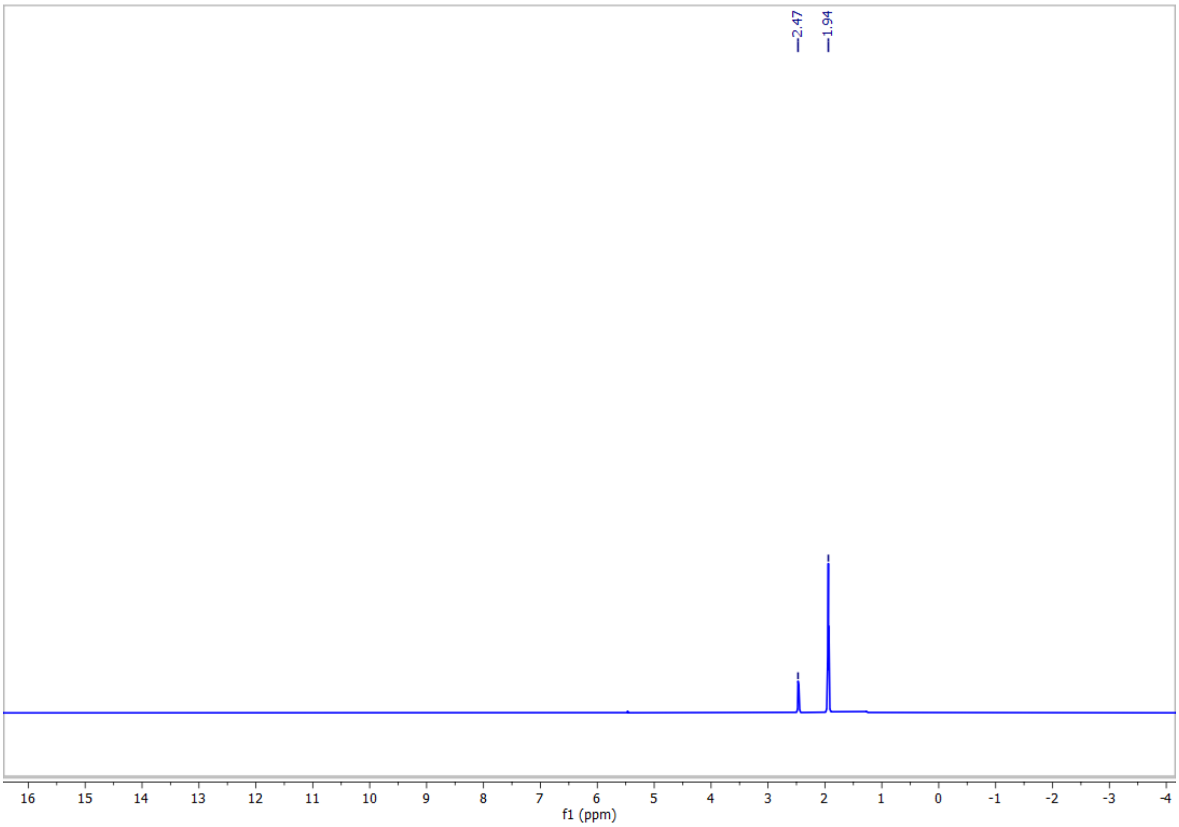

Figure S9. ^{1}H NMR spectrum of pure CH_3CN-d_3 after addition of 1 drop of DMSO.

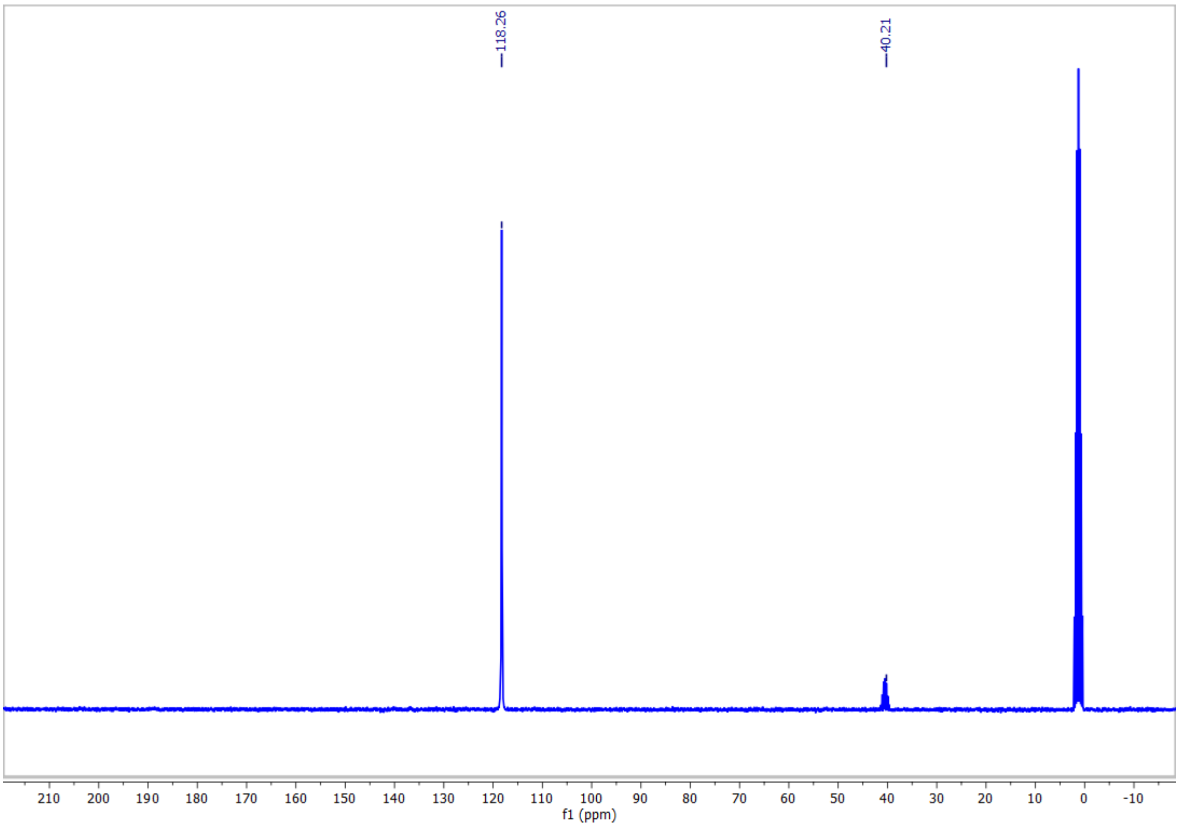

Figure S10. ^{13}C NMR spectrum of pure CH_3CN-d_3 after addition of 1 drop of DMSO.

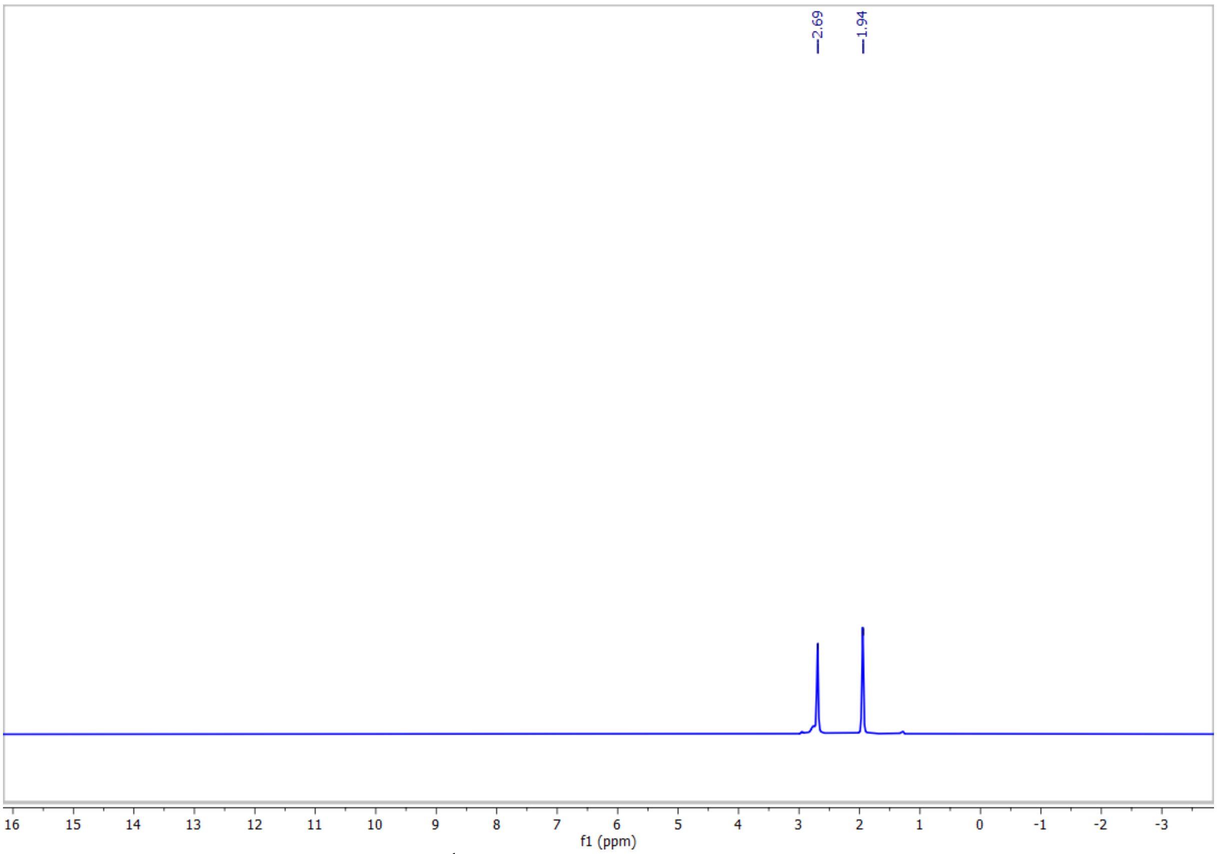

Figure S11. ^{1}H NMR spectrum of **1** in CH_3CN-d_3.

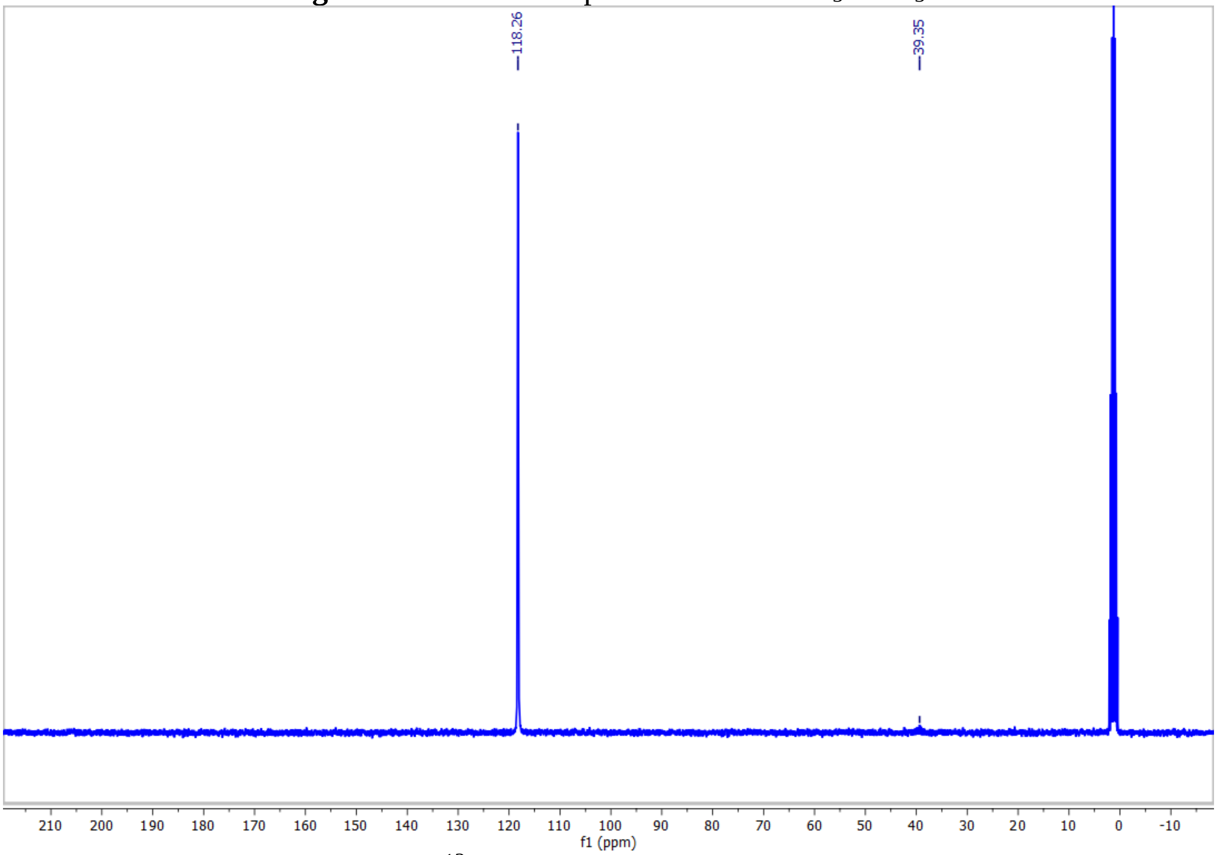

Figure S12. ^{13}C NMR spectrum of **1** in CH_3CN-d_3.

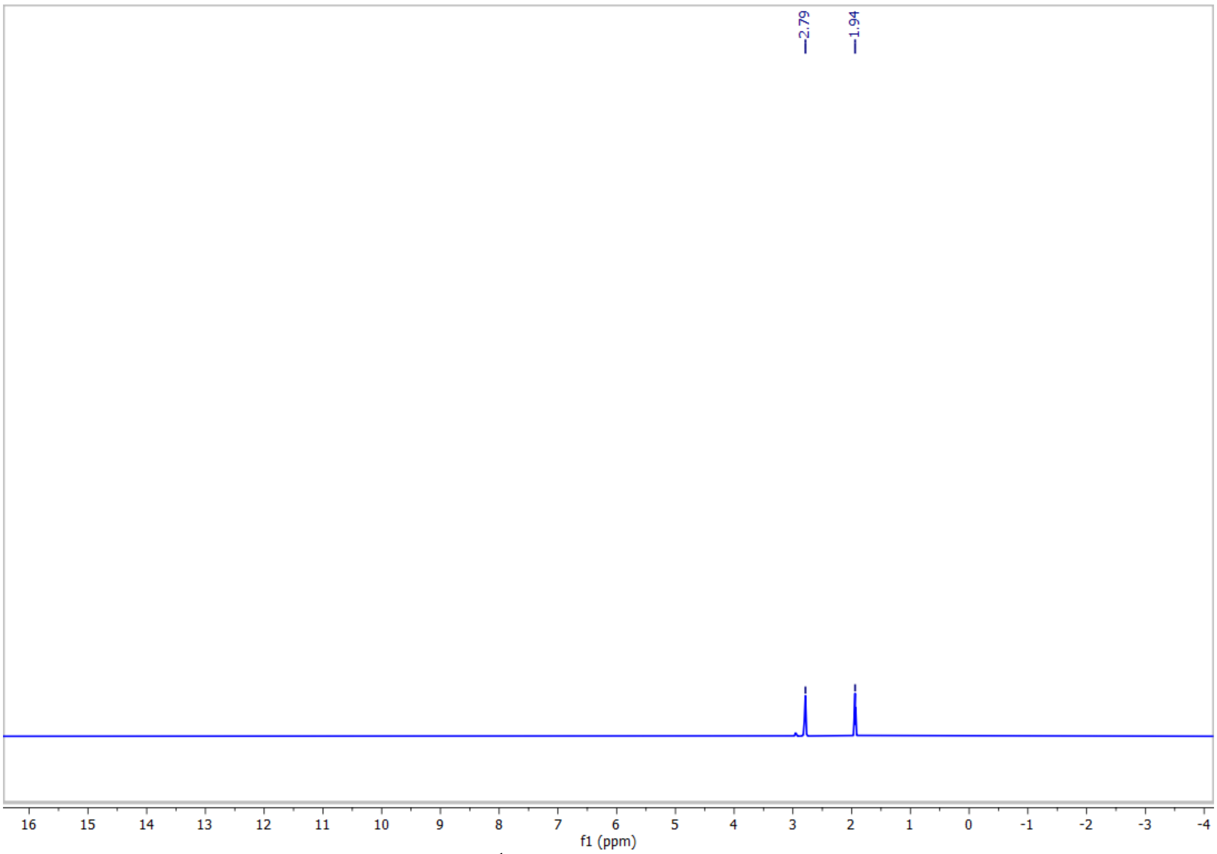

Figure S13. ^1H NMR spectrum of **2** in CH_3CN-d_3.

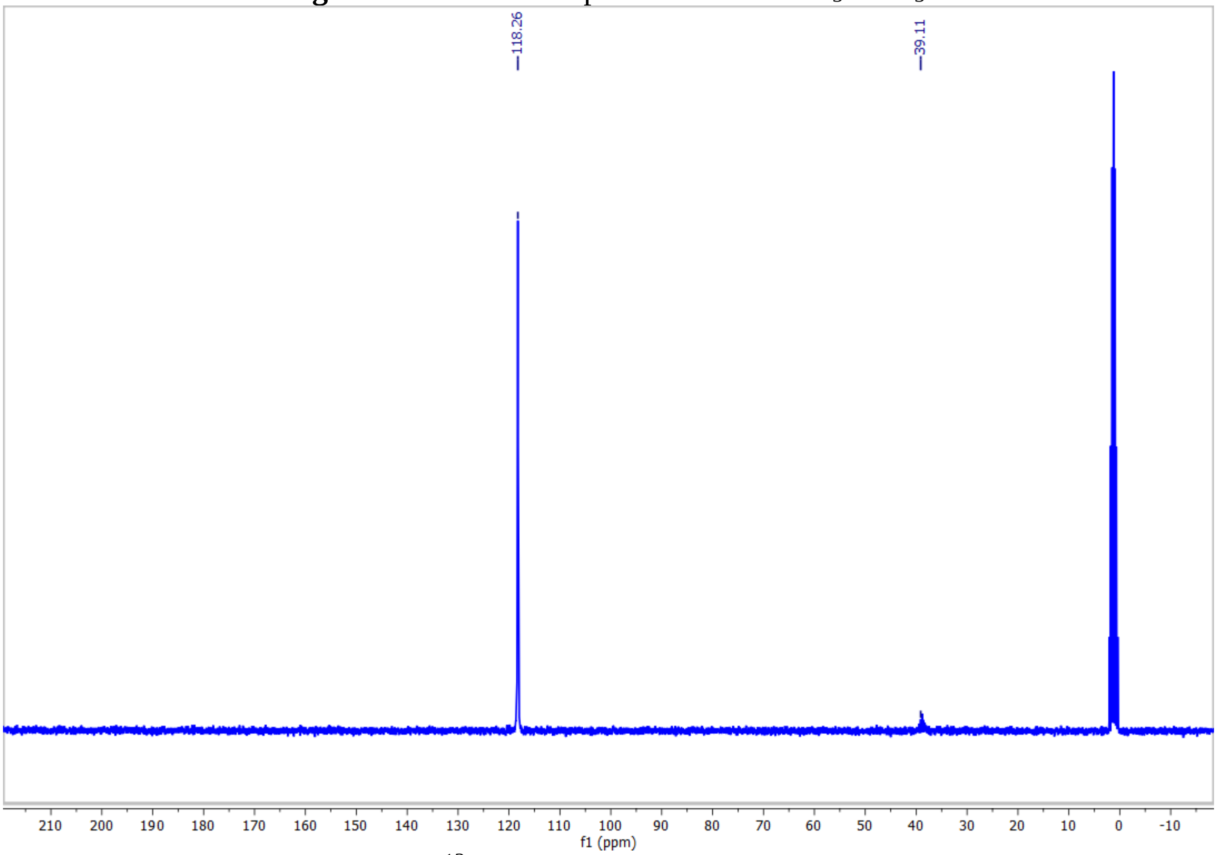

Figure S14. ^{13}C NMR spectrum of **2** in CH_3CN-d_3.

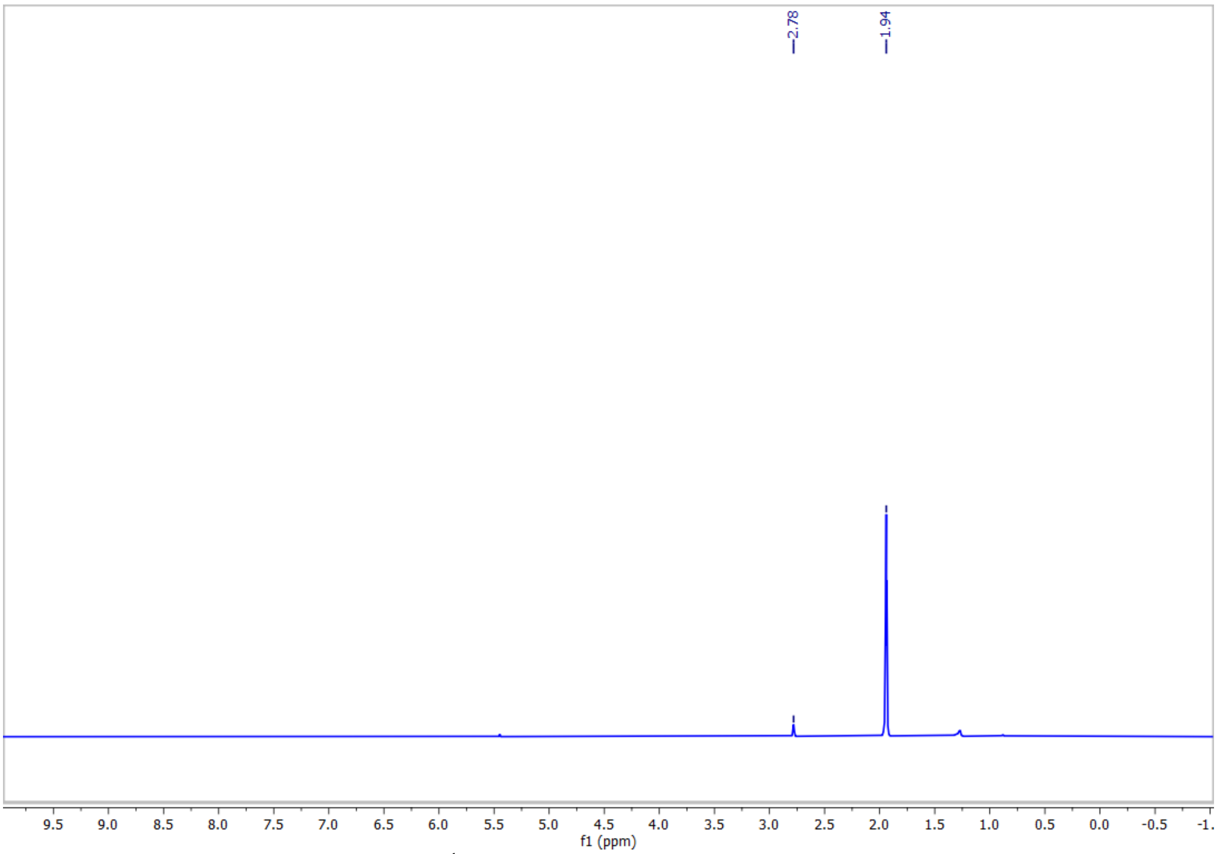

Figure S15. ^{1}H NMR spectrum of **3** in CH_3CN-d_3.

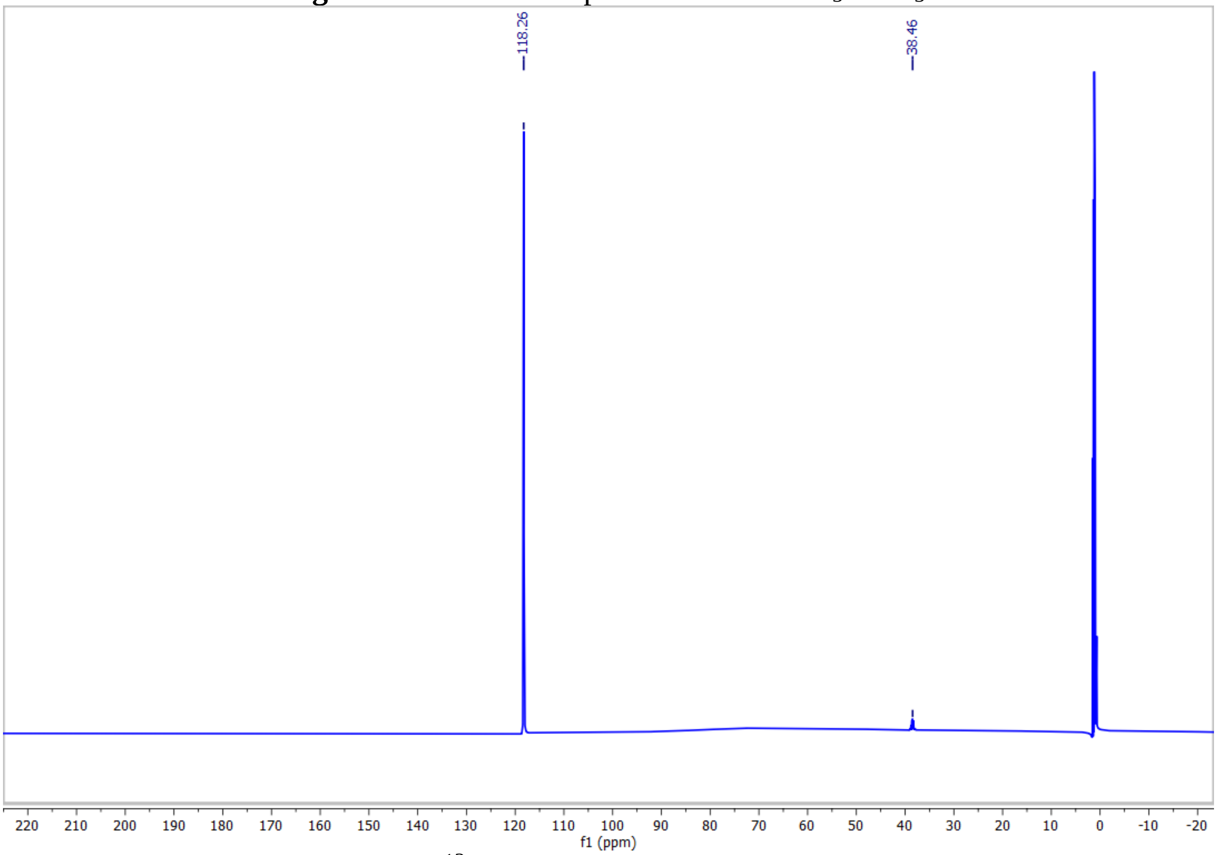

Figure S16. ^{13}C NMR spectrum of **3** in CH_3CN-d_3.

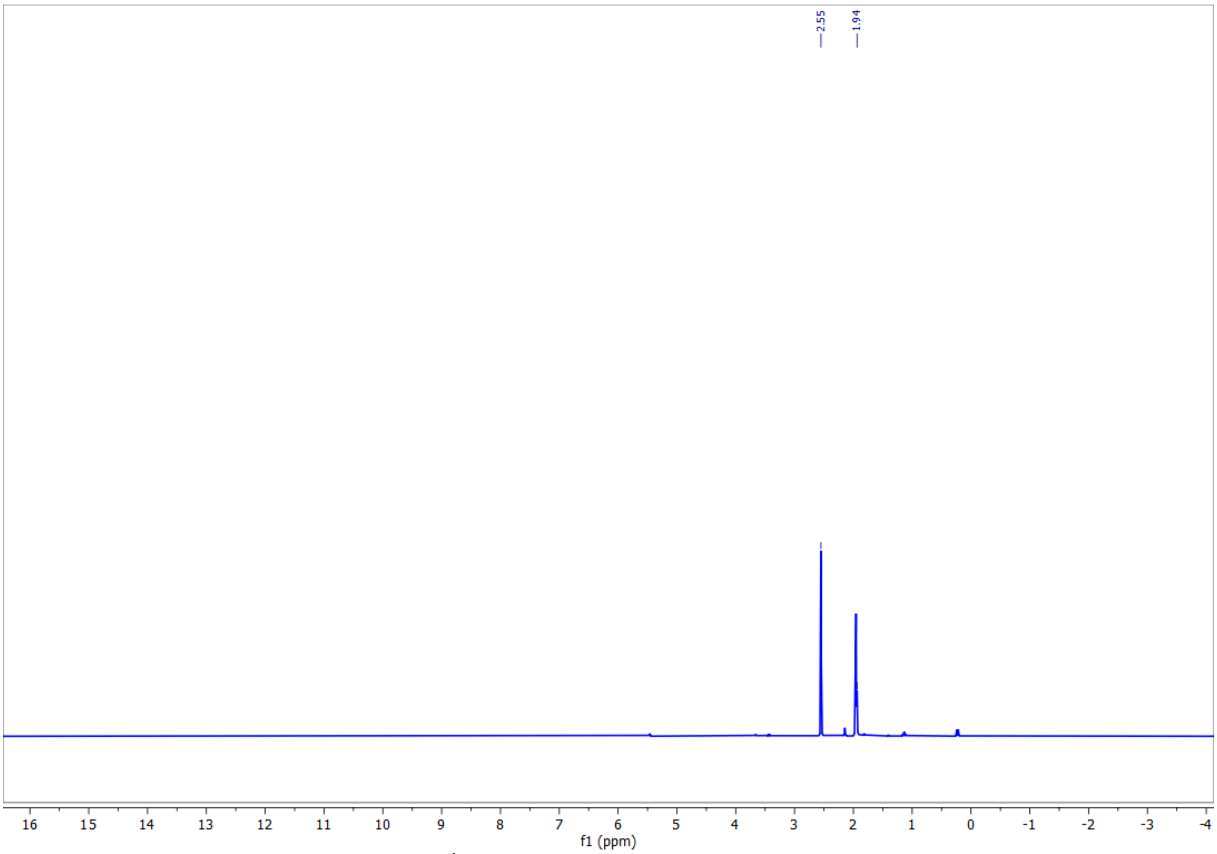

Figure S17. ^{1}H NMR spectrum of **4** in CH_3CN-d_3.

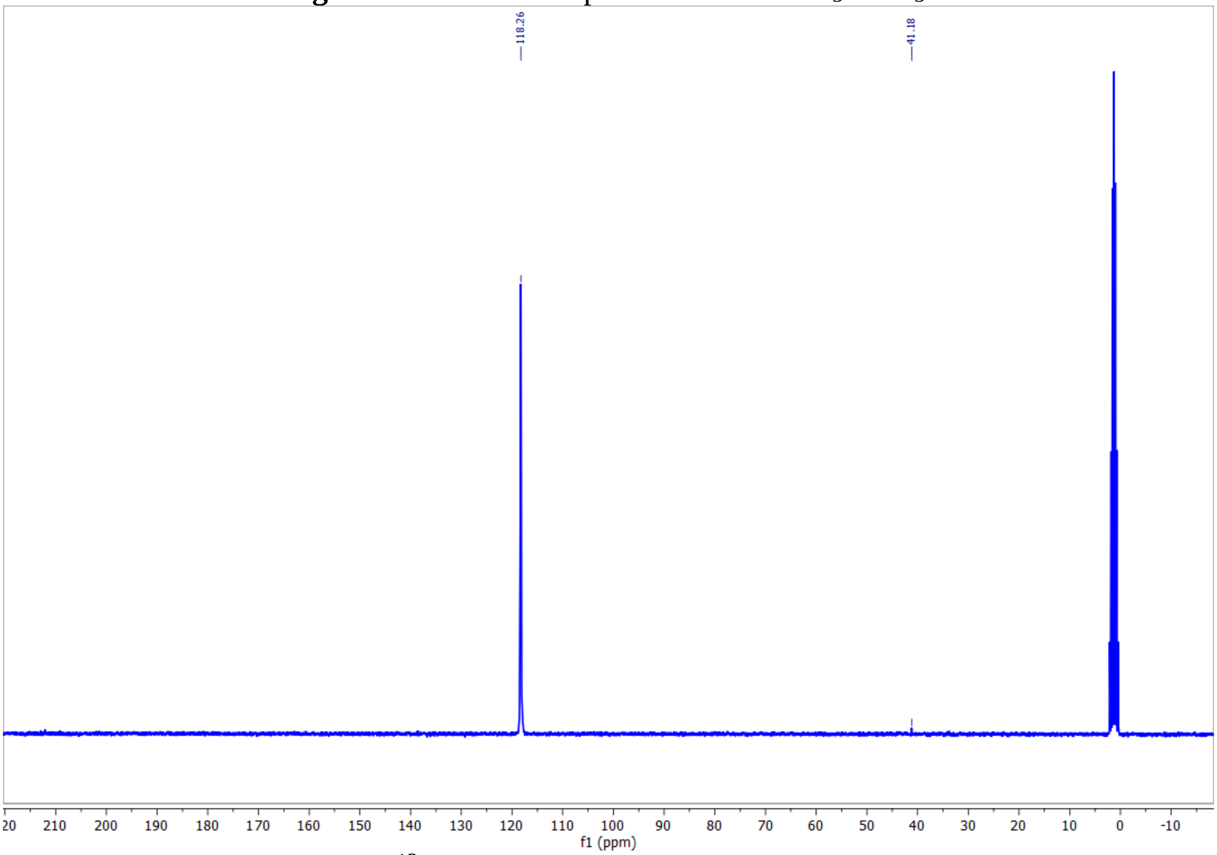

Figure S18. ^{13}C NMR spectrum of **4** in CH_3CN-d_3.

Lewis Acidity

All compounds were analyzed by the (modified) Gutmann-Beckett method in order to gain insights into its Lewis acidity. The acidity of compound **1** is discussed in detail in the manuscript. In addition, ^{1}H NMR data for the Gutmann-Beckett analysis of compound **1** have been obtained:

^{1}H NMR (300 MHz, CD_3CN): δ = 1.17 (dt, 9H, $^{3}J_{H\text{-}H}$ = 7.7 Hz, $^{3}J_{P\text{-}H}$ = 17.6 Hz $PCH_2\mathit{CH_3}$), 2.71 (s, 36H, dmso). The signal for the CH_2 protons of $P\mathit{CH_2}CH_3$ is obscured by the CD_3CN residual peak at 1.96 ppm.

The ^{1}H NMR data for an acetonitrile-d_3 solution containing **1** and $OPEt_3$ show one signal for all dmso ligands. This can be rationalized by two different scenarios: i) one dmso ligand has been substituted by the $OPEt_3$ ligand, but all dmso molecules (five dmso ligands in the coordination sphere of Bi and one free dmso molecule) exchange rapidly on the time scale of the spectroscopic experiment; ii) all dmso ligands remain in the coordination sphere of bismuth, $OPEt_3$ is added as an additional ligand, and the coordination number of bismuth is increased by one. The fact that the chemical shift of the dmso molecules does not approach that of free dmso, but essentially remains the same (in fact a minor downfield shift is even observed) let us tentatively favor the latter interpretation for the main bonding interactions in solution.

A titration of compound **1** with the Gutmann-Beckett donor $OPEt_3$ has been performed (Figure S20), revealing that the Lewis acidity towards the donor remains constant for stoichiometric ratios of $OPEt_3$/**1** = 0-1 (reflected by one resonance with a constant chemical shift in the corresponding ^{31}P NMR spectra). When more than one equivalent of $OPEt_3$ is present, a second signal appears in the ^{31}P NMR spectra, suggesting the presence of a second Lewis acidic site of this species, as previously reported for cationic bismuth compounds.[5] The donor in the second Lewis acidic site seems to be easier susceptible to ligand exchange reactions, as suggested by the chemical shift of the second peak being lower at $OPEt_3$/**1** = 2.0 than at $OPEt_3$/**1** = 4.0, for instance. An ESI-HR-MS analysis was conducted for the reaction mixture containing compound **1** and $OPEt_3$ in a 1:1 stoichiometry, showing the presence of the following species: (ESI(+), MeCN) m/z = 640.155 (640.1503 calculated for $[Bi_1B_1F_4Cl_1(O_1P_1C_6H_{15})_2(N_1C_2H_3)]^{+}$), 323.1018 (323.1031 calculated for $[Bi_1Cl_1(O_1P_1C_6H_{15})_3]^{2+}$), and 390.1462 (390.1462 calculated for $[Bi_1Cl_1(O_1P_1C_6H_{15})_4]^{2+}$). The presence of these fragments in the mass spectrum is evidence of the interaction between compound **1** and $OPEt_3$, while they do not necessarily reflect the dominant type of $[BiCl(OPEt_3)(L)_n]^{2+}$ species in solution (L = neutral ligand). The nature of the detected species was unambiguously verified by careful analysis of the isotope pattern.

The analyses of compound **2** were performed in acetonitrile-d_3 with a 1:2 stoichiometry (i.e. one equivalent of EPR_3 per bismuth atom was used). As a result, acceptor numbers of AN($OPEt_3$) = 68, AN($SPMe_3$) = 36, and AN($SePMe_3$) = 57 were obtained. These values are lower than those obtained for compound **1**. This demonstrates that the dinuclear arrangement of **2** affects its Lewis acidic properties in solution, suggesting a more complex coordination chemistry towards the Gutmann-Beckett donors.
The analyses of compound **3** were performed in acetonitrile-d_3 with a 1:1 stoichiometry (i.e. one equivalent of EPR_3 per bismuth atom was used). As a result, acceptor numbers of AN($OPEt_3$) = 75, AN($SPMe_3$) = 34, and AN($SePMe_3$) = 31 were obtained.
The analyses of compound **4** were performed in acetonitrile-d_3 with a 1:1 stoichiometry (i.e. one equivalent of EPR_3 per bismuth atom of the complex cation was used). As a result, acceptor numbers of AN($OPEt_3$) = 67, AN($SPMe_3$) = 26, and AN($SePMe_3$) = 30 were obtained.

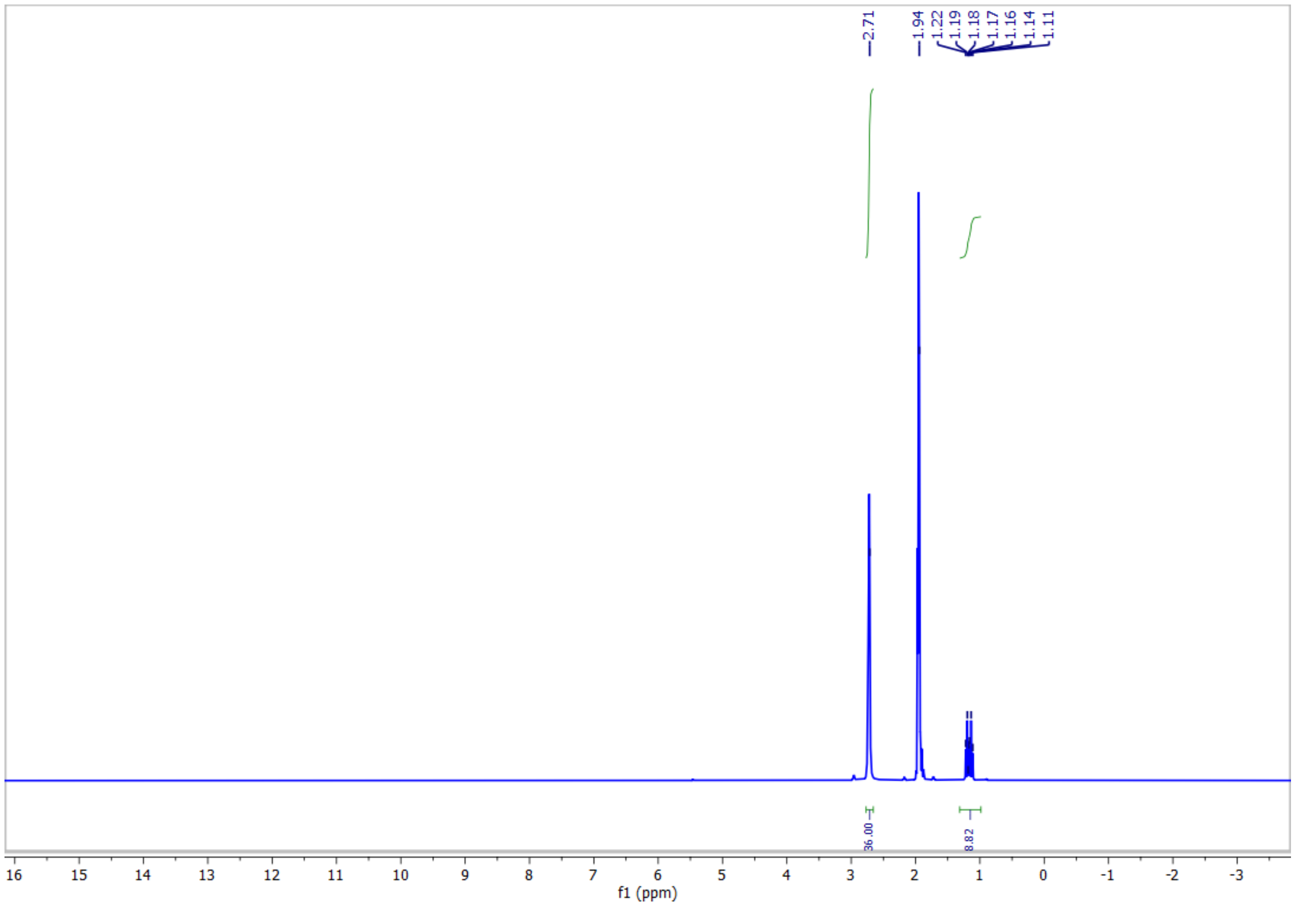

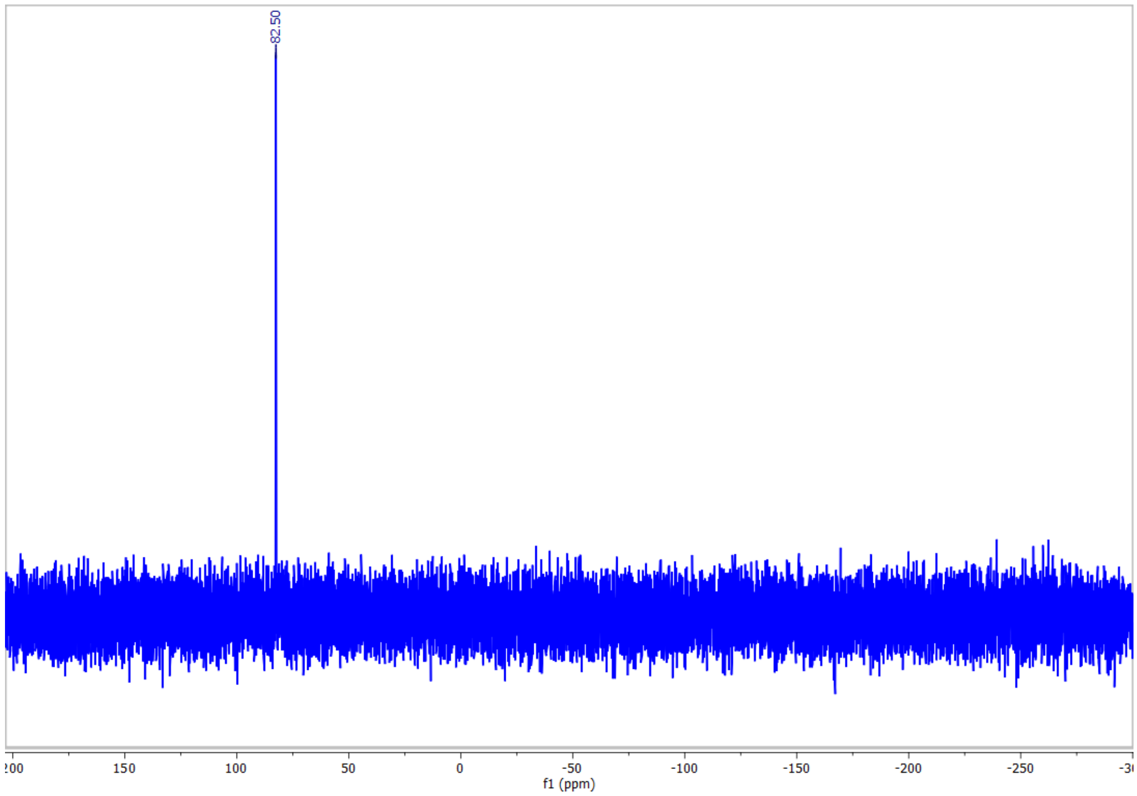

Figure S19. ^{1}H and ^{31}P NMR spectrum of **1** with $OPEt_3$ (molar ratio = 1:1) in CH_3CN-d_3.

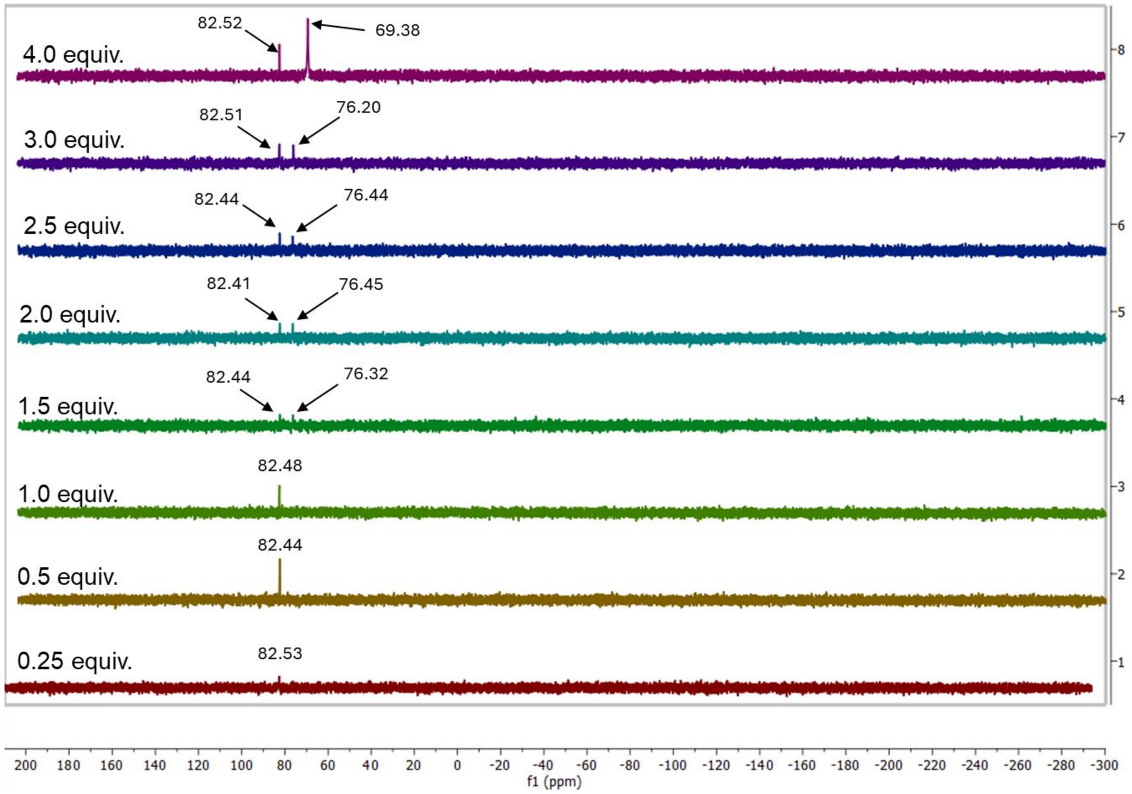

Figure S20. ^{31}P NMR spectra of **1** in the presence of varying amounts of $OPEt_3$ (0.25 – 4.0 equiv.) in CH_3CN-d_3.

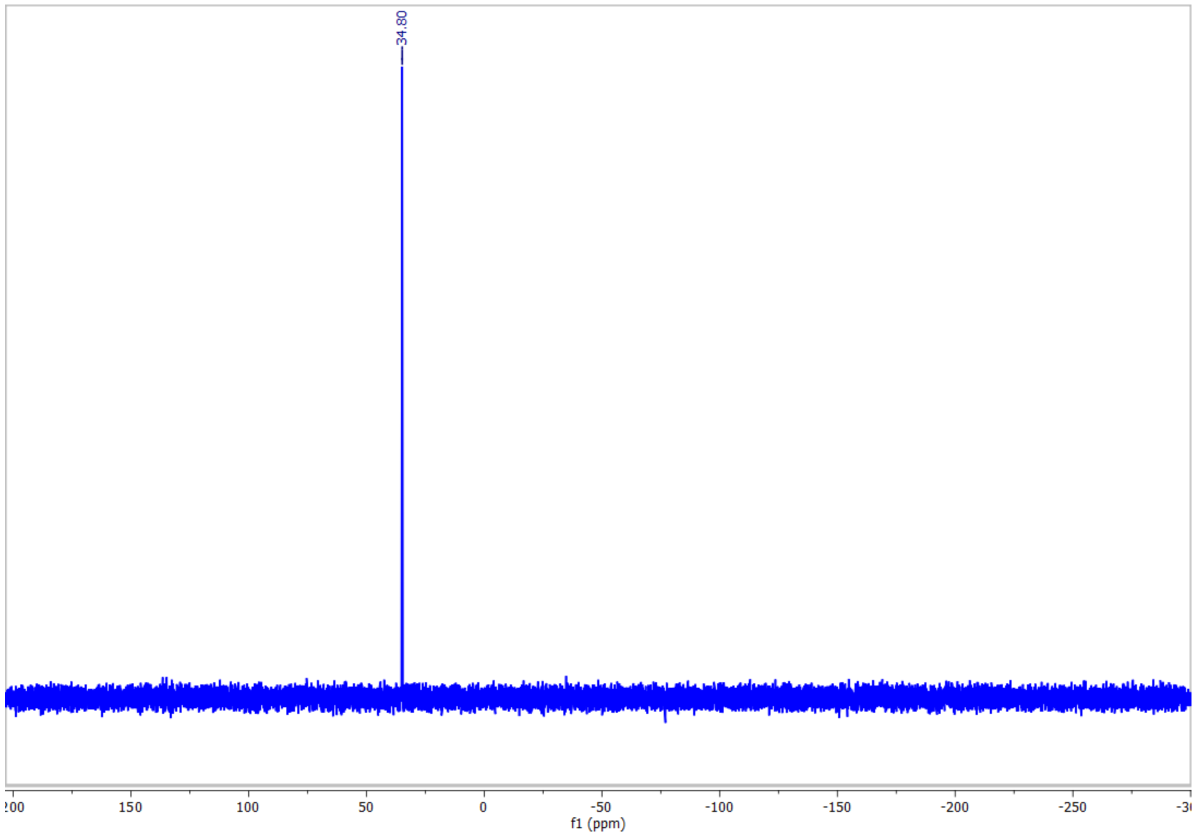

Figure S21. ^{31}P NMR spectrum of **1** with $SPMe_3$ (molar ratio = 1:1) in CH_3CN-d_3.

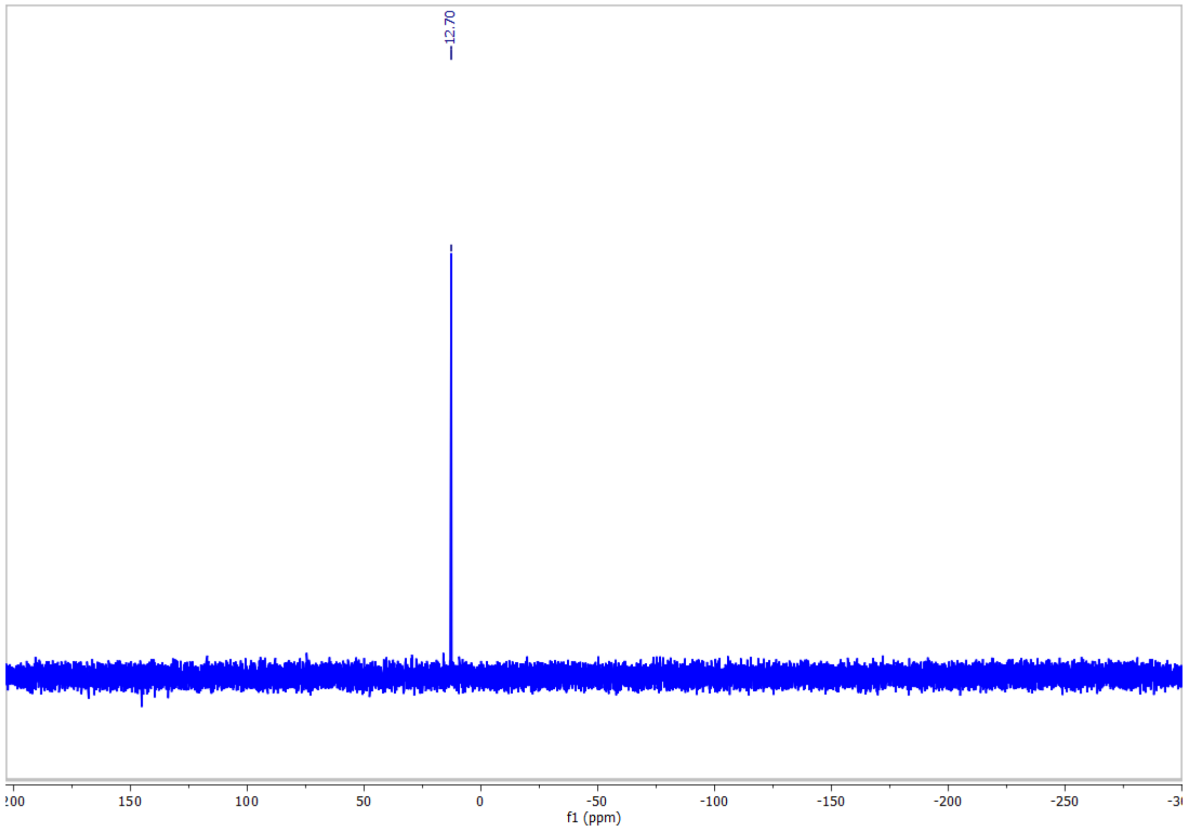

Figure S22. ^{31}P NMR spectrum of **1** with $SePMe_3$ (molar ratio = 1:1) in CH_3CN-d_3.

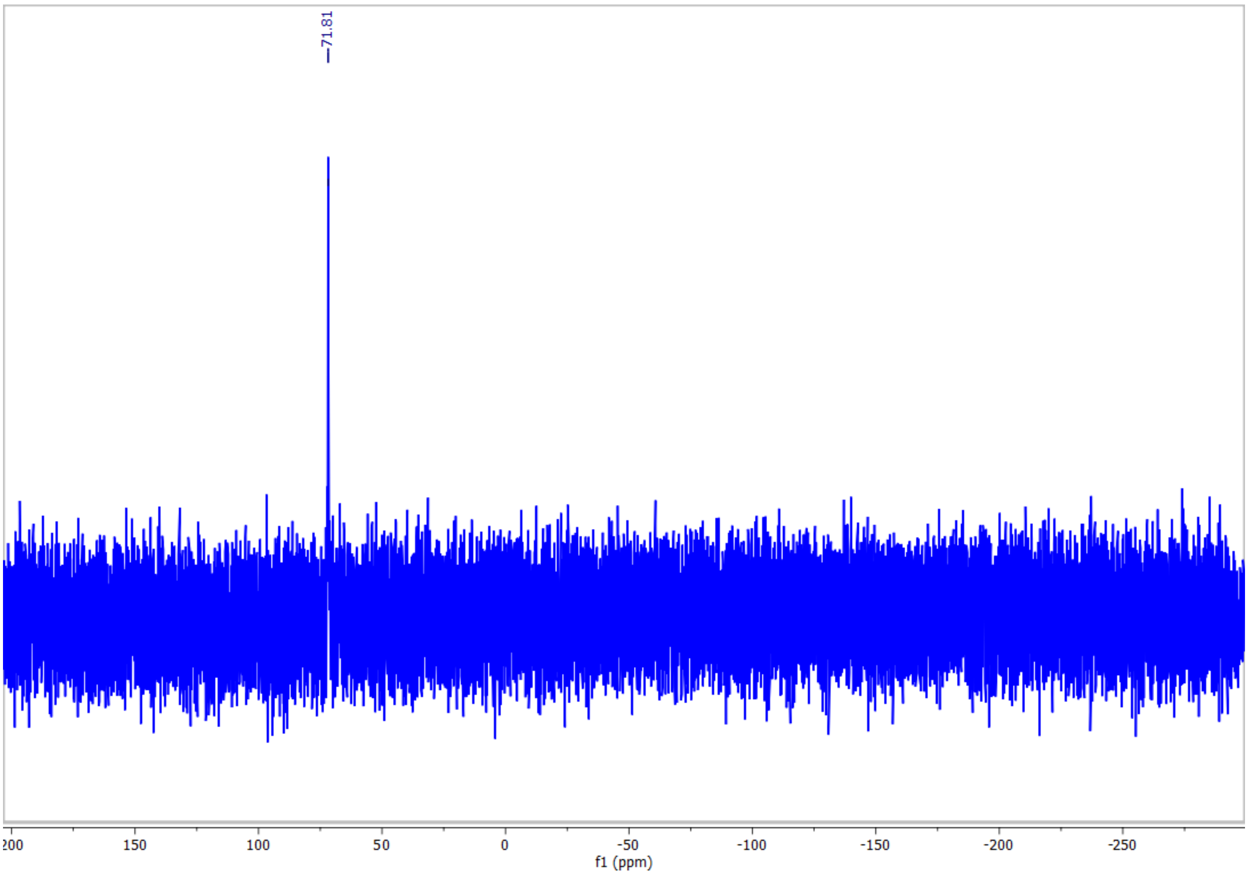

Figure S23. ^{31}P NMR spectrum of **2** with $OPEt_3$ (molar ratio = 1:2) in CH_3CN-d_3.

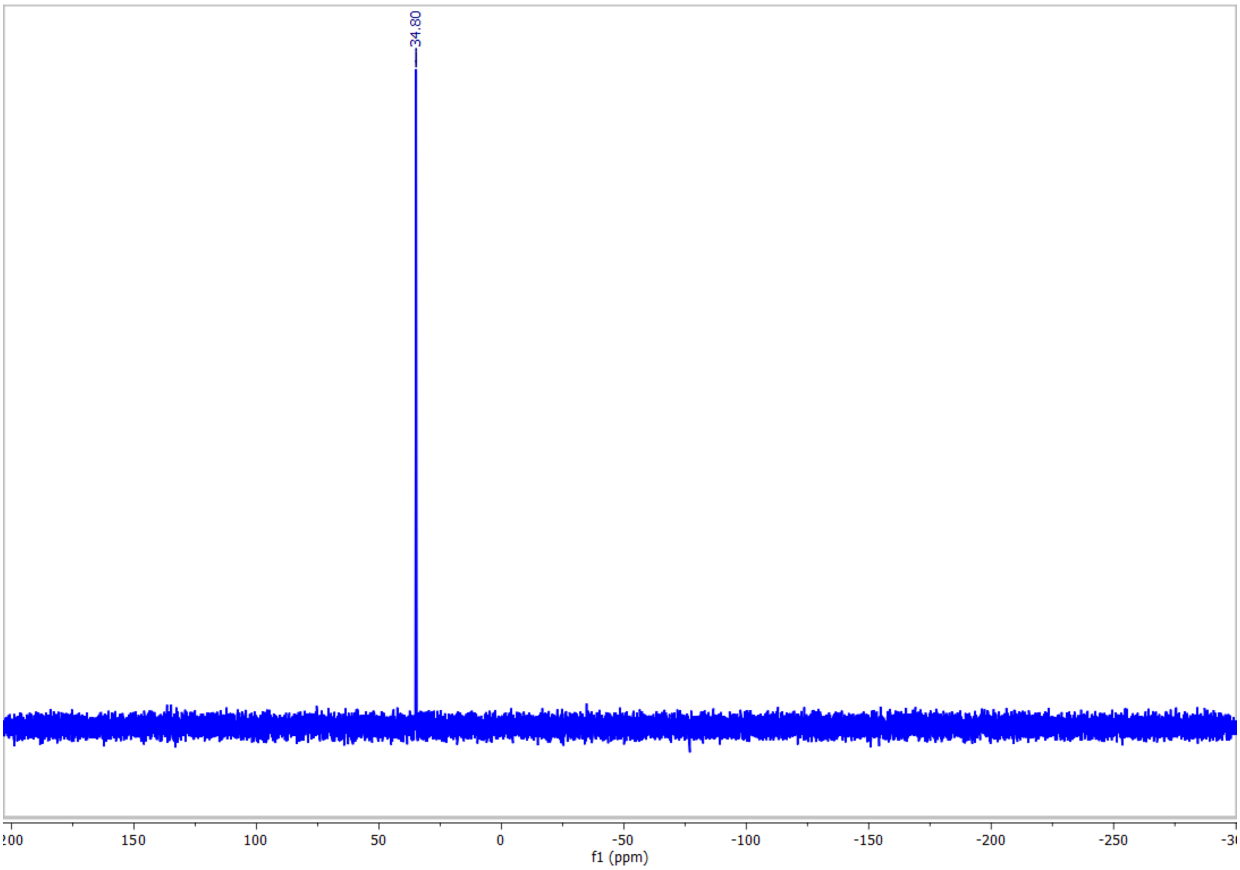

Figure S24. ^{31}P NMR spectrum of **2** with $SPMe_3$ (molar ratio = 1:2) in CH_3CN-d_3.

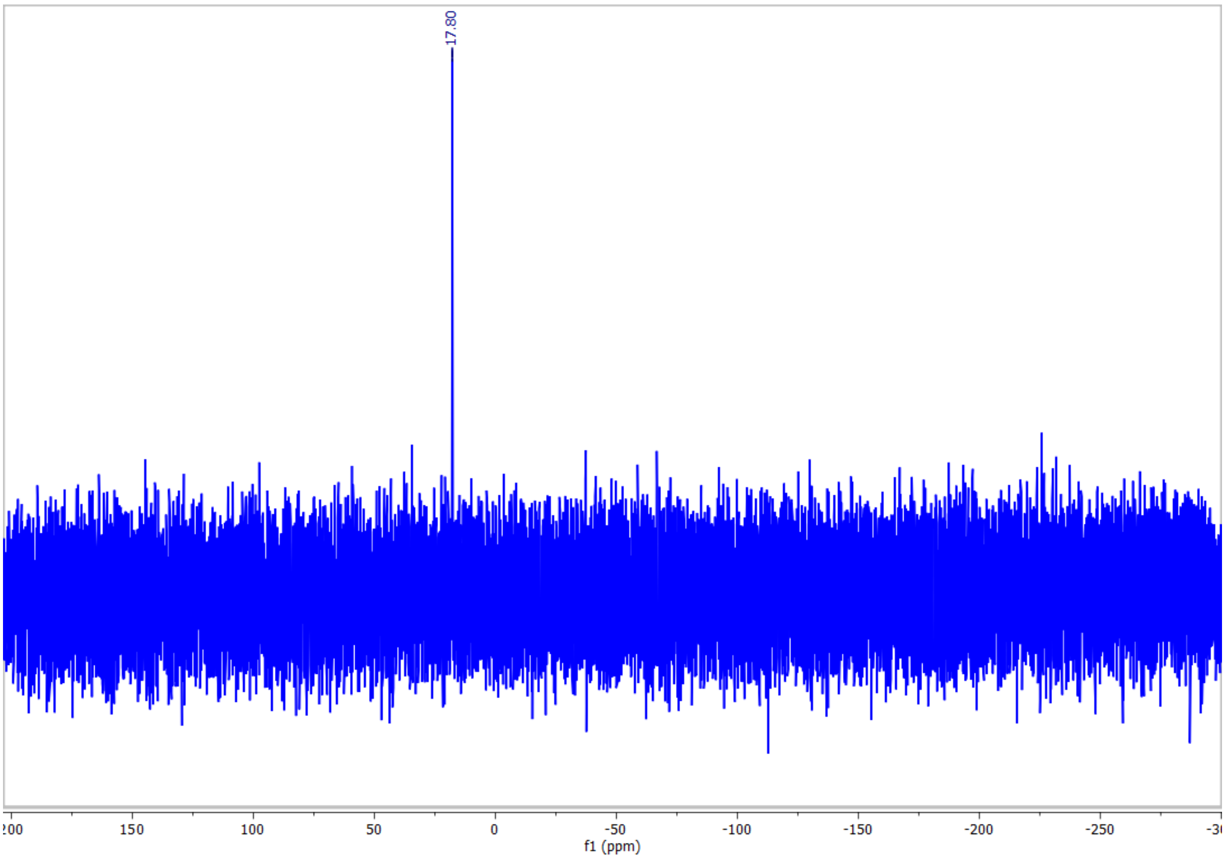

Figure S25. ^{31}P NMR spectrum of **2** with $SePMe_3$ (molar ratio = 1:2) in CH_3CN-d_3.

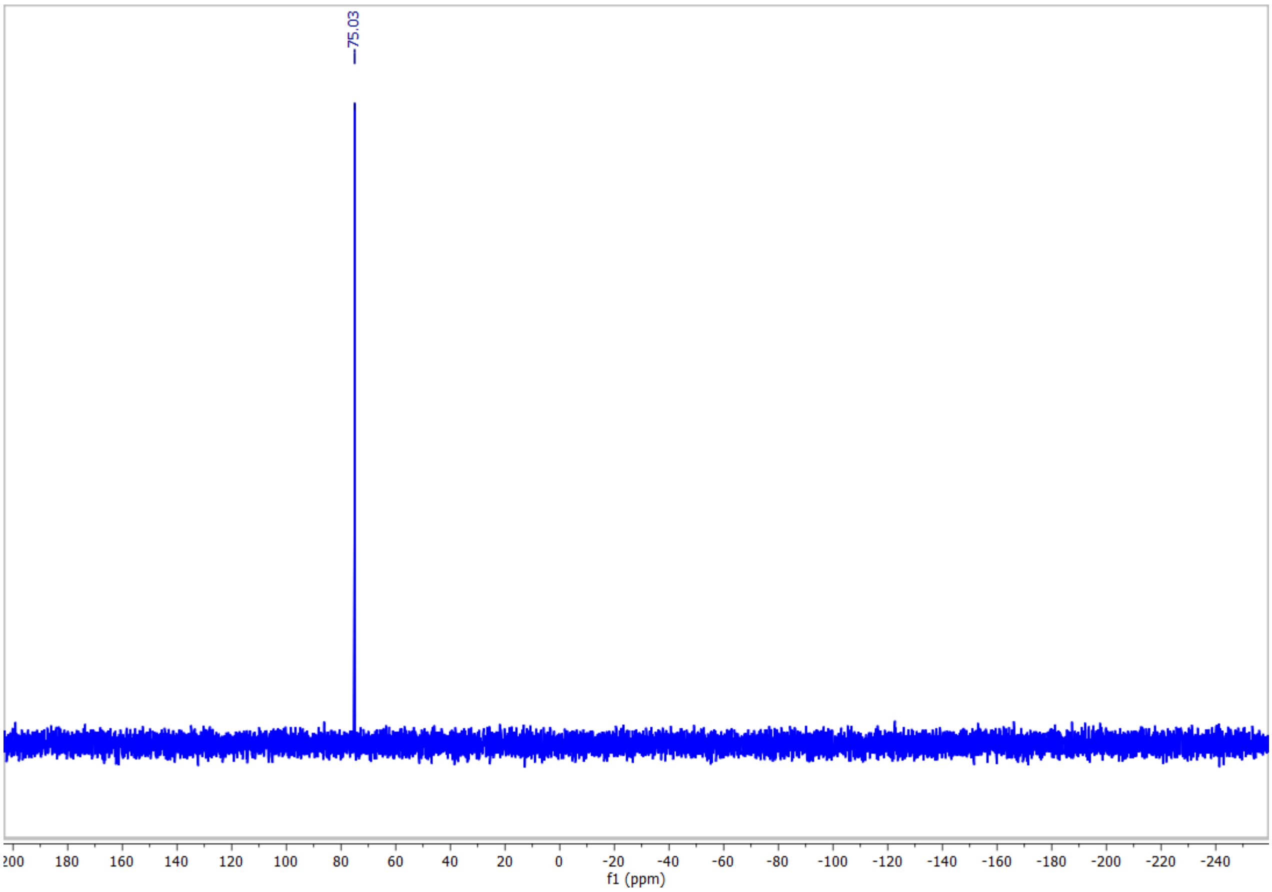

Figure S26. ^{31}P NMR spectrum of **3** with $OPEt_3$ (molar ratio = 1:1) in CH_3CN-d_3.

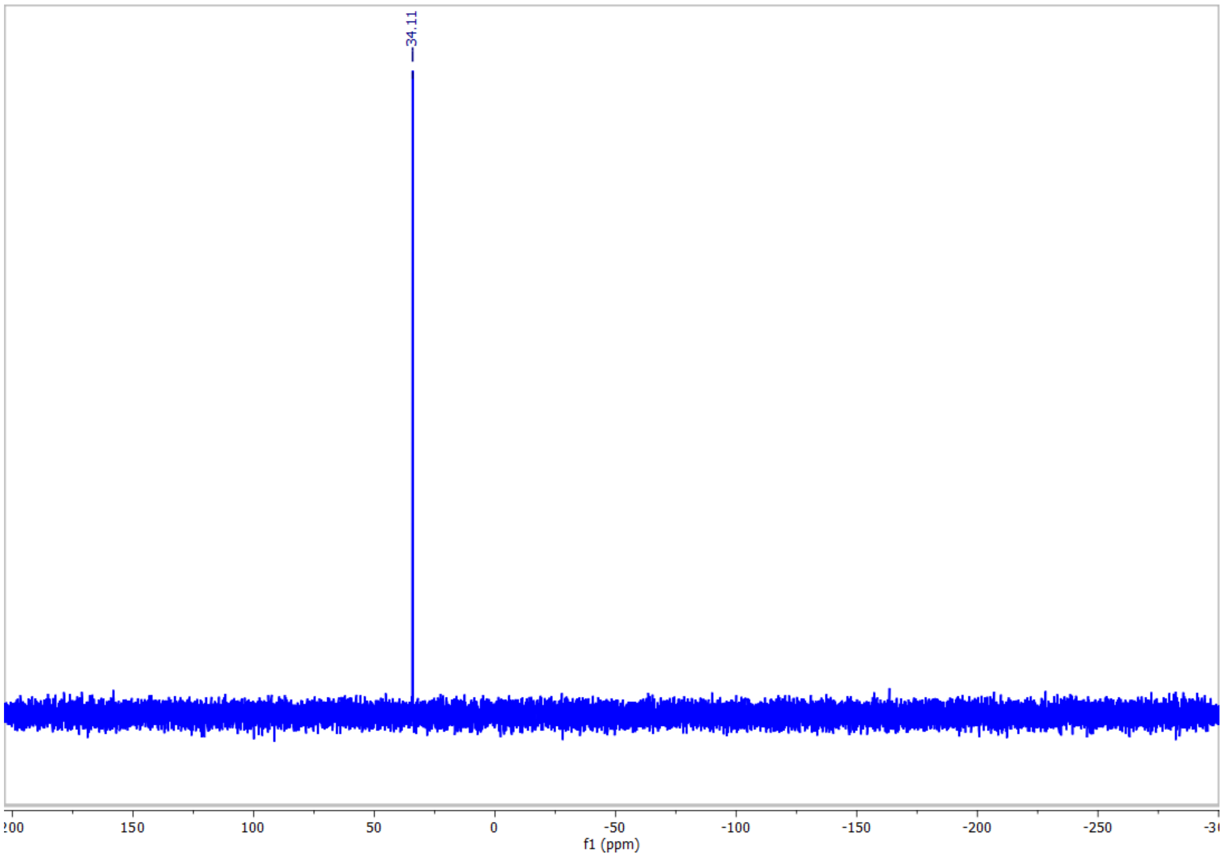

Figure S27. ^{31}P NMR spectrum of **3** with $SPMe_3$ (molar ratio = 1:1) in CH_3CN-d_3.

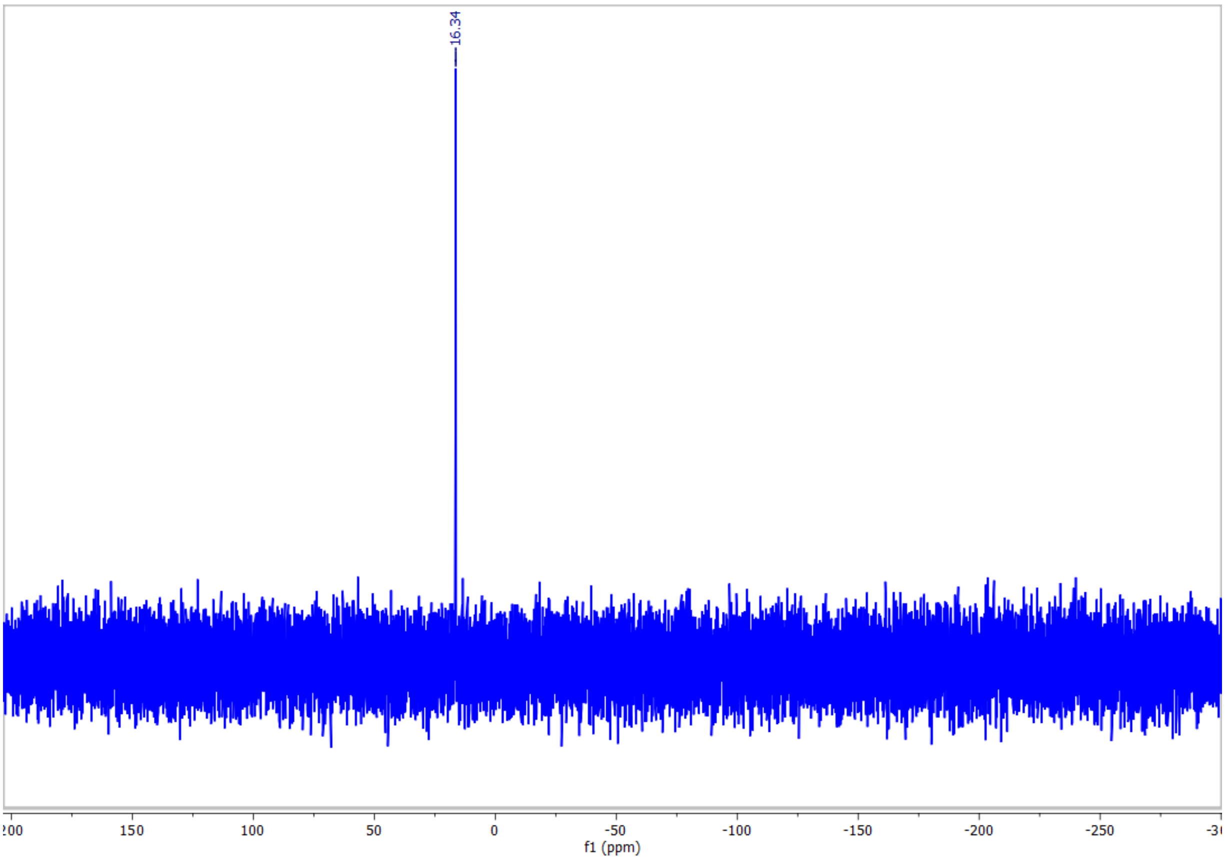

Figure S28. ^{31}P NMR spectrum of **3** with $SePMe_3$ (molar ratio = 1:1) in CH_3CN-d_3.

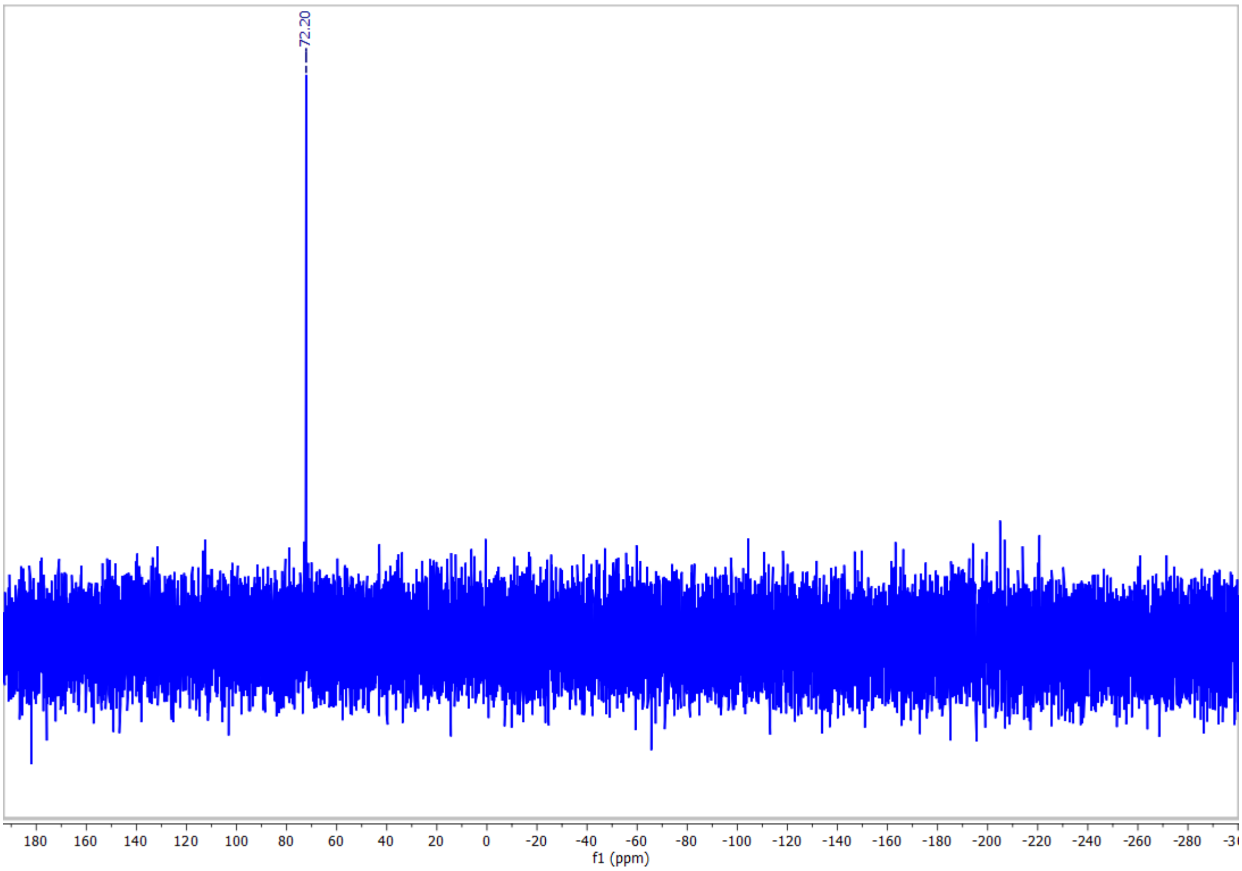

Figure S29. ^{31}P NMR spectrum of **4** with $OPEt_3$ (molar ratio = 1:1) in CH_3CN-d_3.

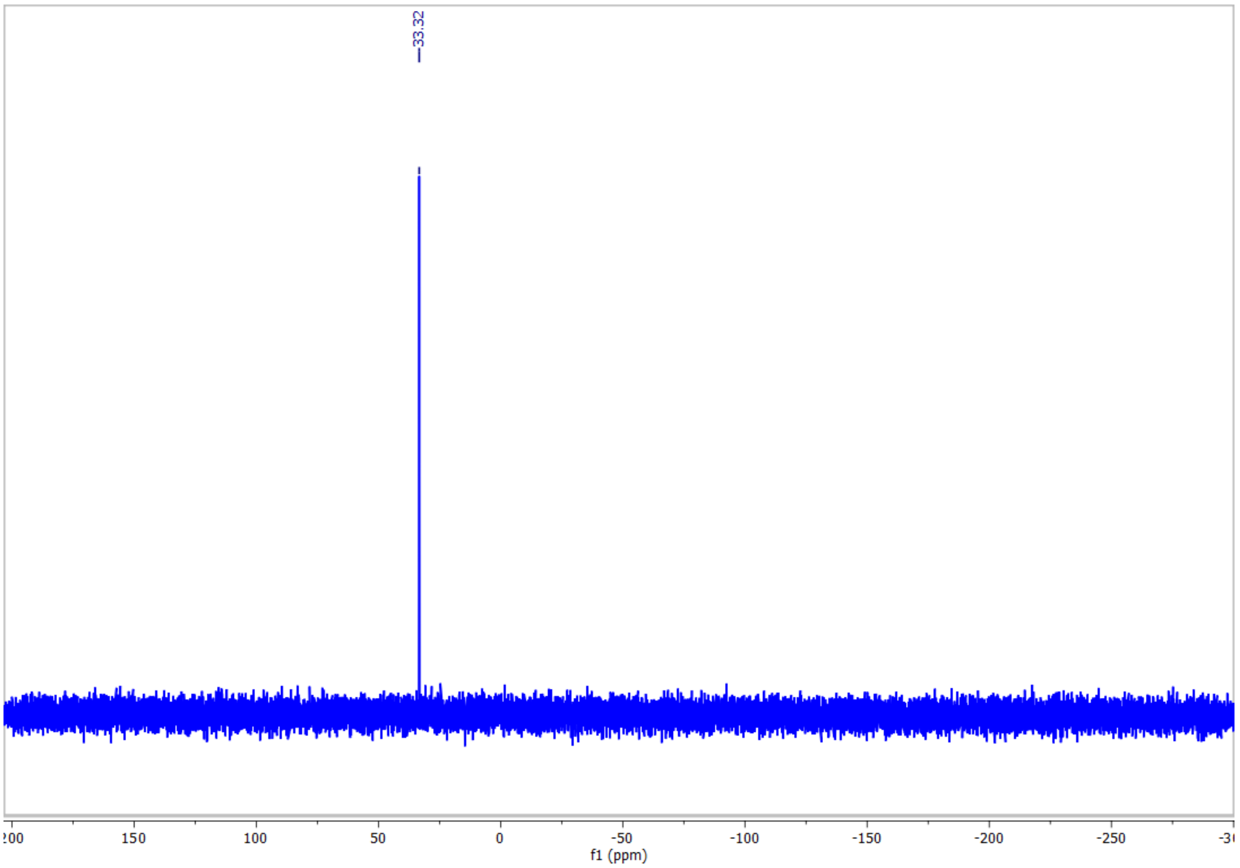

Figure S30. ^{31}P NMR spectrum of **4** with $SPMe_3$ (molar ratio = 1:1) in CH_3CN-d_3.

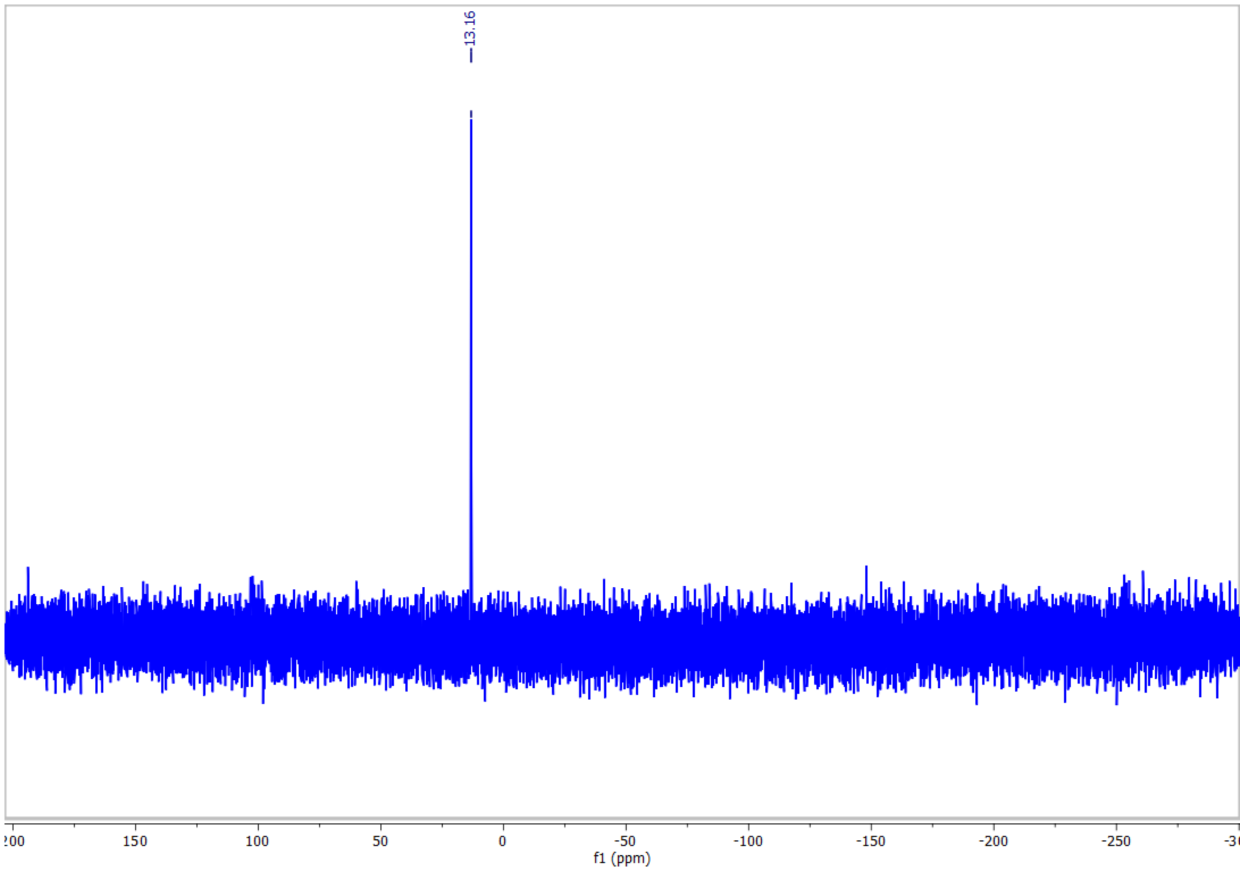

Figure S31. ^{31}P NMR spectrum of **4** with $SePMe_3$ (molar ratio = 1:1) in CH_3CN-d_3

UV-vis Spectrum and Tauc plot of 4

Table S12. Band gap (in eV) for selected organic–inorganic halogenido metalates.

Compound	Optical Band gap [eV]
$(HPy)_3Bi_2I_9$, Py=pyridine	2.01[6]
$(C_3N_2H_5)_3Bi_2I_9$	1.97[7]
$(CH_3NH_3)_3Bi_2I_9$	1.94[8]
$(NH_2CHNH_2)_3Bi_2I_9$	1.85[9]
$(S(CH_3)_3)_3Bi_2I_9$	2.10[10]
$(Hpyz)_3Bi_2I_9 \cdot 2H_2O$, pyz=pyrazine	1.98[11]
BiI_3	1.73[12]
$[Bi(DMSO)_8][Bi_2I_9]$	2.17[12]
$[La(DMSO)_8][Bi_2I_9]$	2.21[12]
$(PhCH_2NEt_3)_3(Bi_2I_9)$	2.19[13]
$(CH_3ImC_5)_3(Bi_2I_9)_2$, Im = imidazole	2.15[14]
$[(CH_3)_3NH]_3Bi_2I_9$	2.00[15]
$(PhCH_2NH_3)_3Bi_2I_9$	2.23[16]

**[Thiazole]$_3$[Bi$_2$I$_9$]**	2.00[17]
(EMP)$_3$(Bi$_2$I$_9$) (EMP$^+$ = N-ethyl-4-methyl-pyridinium)	2.17[18]
[Ln(DMF)$_8$][Bi$_2$I$_9$] [Ln = Y, Tb]	1.90–2.15[19]
[*N*,*N*-dimethylimidazole]$_3$Bi$_2$I$_9$	2.10
(N-MePy)$_3$[Bi$_2$I$_9$]·CH$_3$CN	1.87[20]
(N-EtPy)$_3$[Bi$_2$I$_9$]·CH$_3$CN	2.21[20]
[L$_1$][Bi$_2$I$_9$], (L$_1^{3+}$ = 1,1′,1″-(benzene-1,3,5)tris(3-methyl-1*H*-imidazol-3-ium))	2.28[21]
(cy-hexyl-NH$_3$)$_3$Bi$_2$I$_9$	2.47[22]
(4-methylpiperidinium)Bi$_2$I$_9$	2.05[23]
[Dy(DMF)$_8$](Bi$_2$I$_9$)	2.19[24]
(bis-(2-dimethylaminoethyl) ether)$_2$IBi$_2$I$_9$	1.92[25]
Rb$_3$Bi$_2$I$_9$	1.93[26]
{[Ln(dpdo)(DMF)$_6$](Bi$_2$I$_9$)$_2$}$_n$ (Ln = La, Pr, Er), (bpdo = 4,4′-bipyridine *N*,*N*′-dioxide)	2.16, 2.13 and 2.09 eV[27]
**4**	1.71[This work]

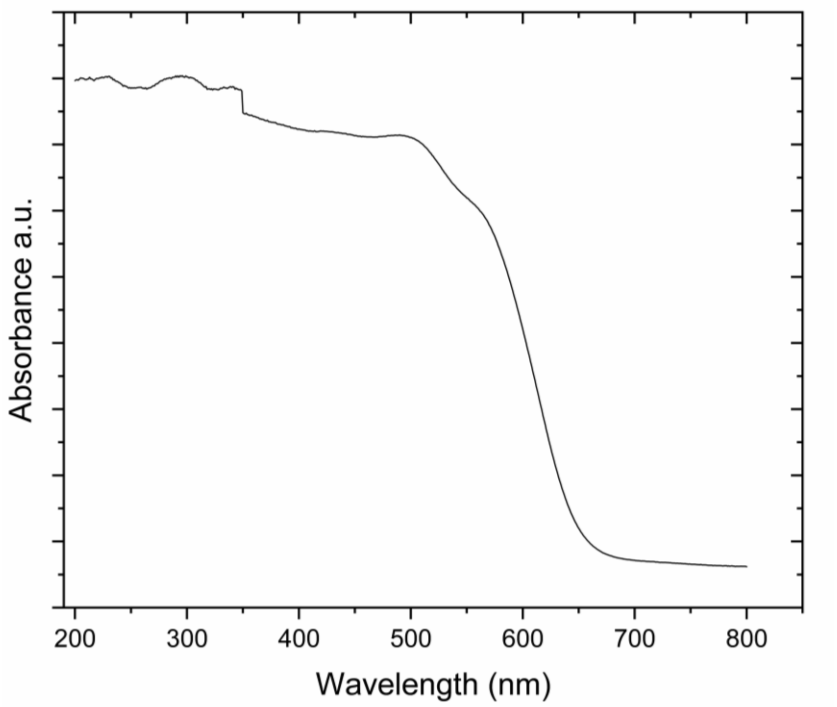

Figure S32. Solid UV-Vis. spectrum of **4** (298 K).

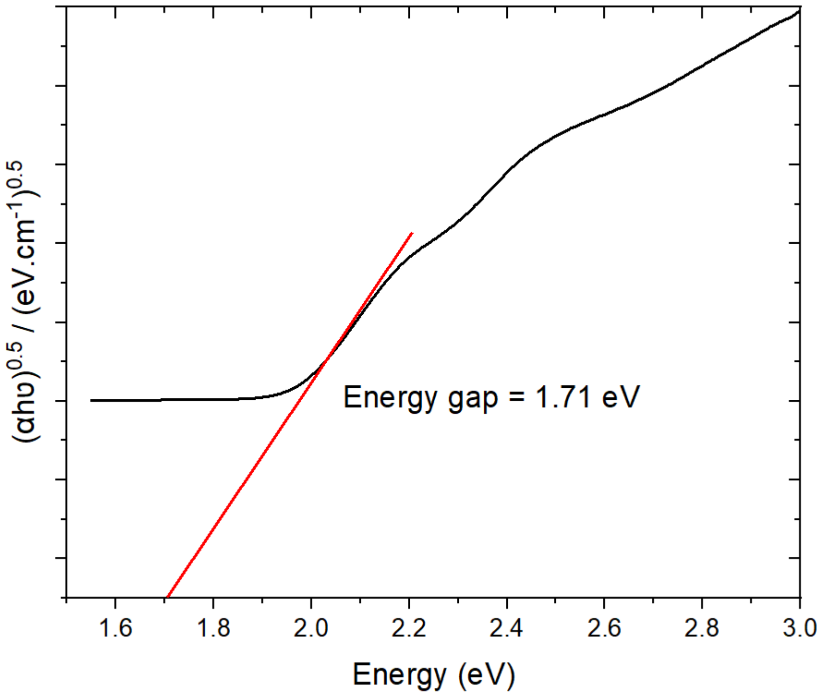

Figure S33. Optical-band-gap determination via Tauc coordinates for **4** (298 K).

[Bi(dmso)$_8$][Bi$_2$I$_9$]·CH$_3$CN (**4**) crystallizes in the triclinic space group $P\overline{1}$ with Z = 2 (Figure S5). The structure is characteristic of [Bi$_2$I$_9$]$^{3-}$ anionic clusters and solvated bismuth cations acting as charge compensators. The cation of **4** consists of a single bismuth atom coordinated by eight DMSO molecules, arranged in a square antiprismatic geometry. The bismuth atoms within the Bi$_2$I$_9$ cluster are coordinated in distorted octahedral environments and consists of only two, face-sharing BiI$_6$ octahedra also sits on a site of D_3 crystallographic symmetry.

The Bi–I bond lengths in **4** range from 2.968(6)–3.283(8) Å. Notably, Bi–I$_{bridging}$ bond lengths (3.178(8)–3.283(8) Å) are significantly longer than Bi–I$_{terminal}$ bond lengths (2.968(6)–2.991(7) Å). There is still some distortion in BiI$_6$ octahedra, which can also be judged from the I–Bi-I bond angles. Specifically, I$_{terminal}$-Bi-I$_{terminal}$ angles are greater than 90° (91.42−95.93°), while I$_{bridging}$-Bi-I$_{bridging}$ angles being smaller than 90° (80.15−81.51°). This distortion could be ascribed as the start of localization of the lone pairs trans to the Bi–Bi vector, or a geometric arrangement aimed at reducing the Bi–Bi interaction. [28] The stereochemical activity of the bismuth center, which results from its 6s^2 nonbonding lone pair electrons, can be explained by Brown's model. [29] According to this model, the coordination polyhedron of the main group nS^2 elements is based on an octahedron that enables the coordination of the lone pair cation in three possible ways: (a) with 2 strong, 2 intermediate, and 2 weak bonds; (b) with 3 strong and 3 weak bonds; (c) with 1 strong, 1 weak and 4 intermediate bonds. Based on the Bi-I distances, it can be inferred that the geometry of BiI$_6$ octahedra conforms to distortion type (b) and exhibits a stereochemical activity. [24]

The bond lengths and angles differ slightly from that of $[Bi(dmso)_8][Bi_2I_9]$ (2.913(4)–3.328(4) Å, 80.19−96.76°) although the difference between the two structures is one uncoordinated acetonitrile molecule in **4**. $[Bi(dmso)_8][Bi_2I_9]$ was prepared by dropwise dissolving BiI_3 in DMSO. [12, 30, 31] It is important to note that no I···I distances in the compounds fall closer than twice the van der Waals radius of I, indicating the absence of I···I interactions in **4**. [31]

4 has excellent absorbing properties (Figures S32, 33), which can be attributed to the iodobismuthate anion responsible for its red color. The absorption spectra of **4** show a prominent absorption band in the range 370-600 nm in the UV-vis. spectrum. This absorption behavior is primarily attributed to the iodobismuthate anion, as the solvated metal cation has negligible direct contributions to the conduction and/or valence bands of the anionic framework. [32] Analysis of the absorption spectra between 200 and 800 nm reveals an optical bandgap of 1.71 eV, as determined from a Tauc plot. This value is comparable to or even lower than those observed for similar compounds such as $[CH_3NH_3)_3][Bi_2I_9]$, which exhibits an indirect bandgap at 1.94 eV. Consequently, this material can be classified as semiconductor, aligning with the characteristics of two-dimensional semiconducting materials. [8, 12, 33] The bismuthates will generally display values of E_g ranging from 1.73 to 2.47 eV as illustrated in Table S12. However, it is important to note that the values of E_g differ significantly depending on the used method and the nature of sample (e.g., single crystals, powders or thin films). [34]

IR Spectroscopy

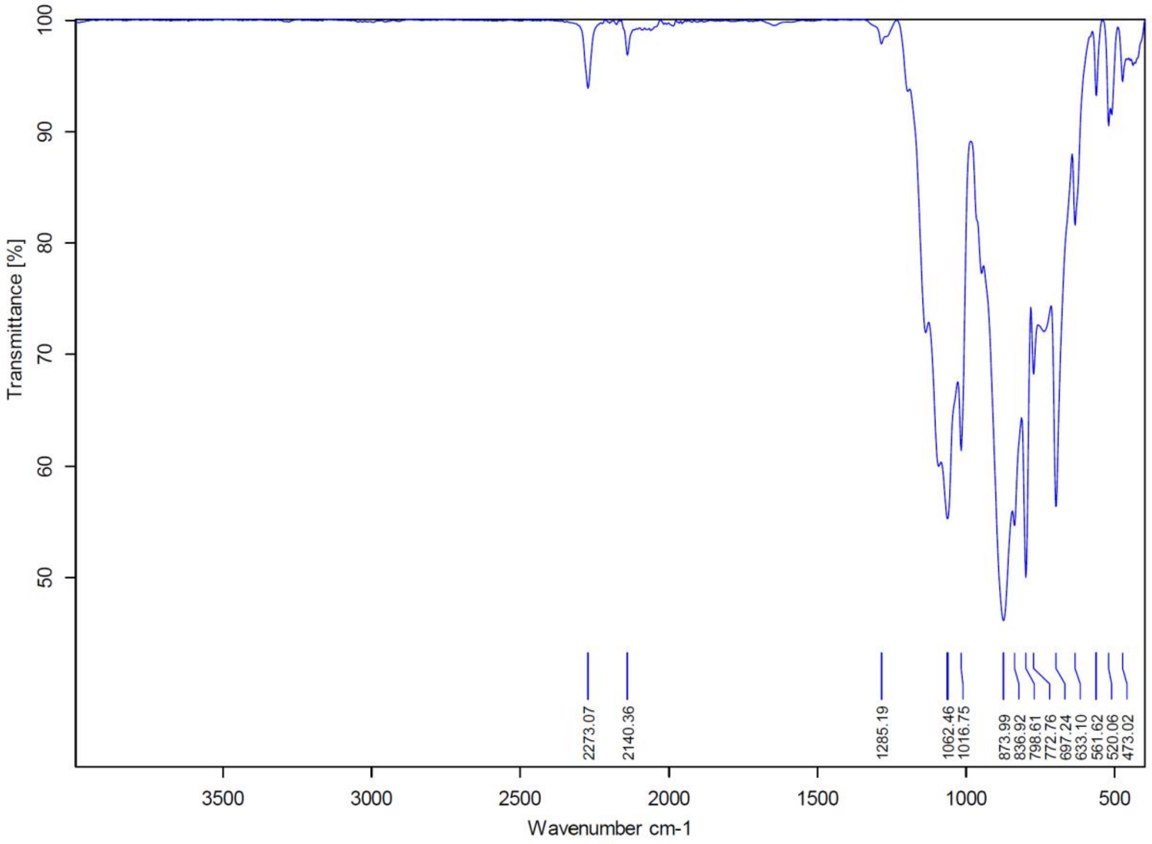

Figure S34. Solid-state-IR spectrum of **1**.

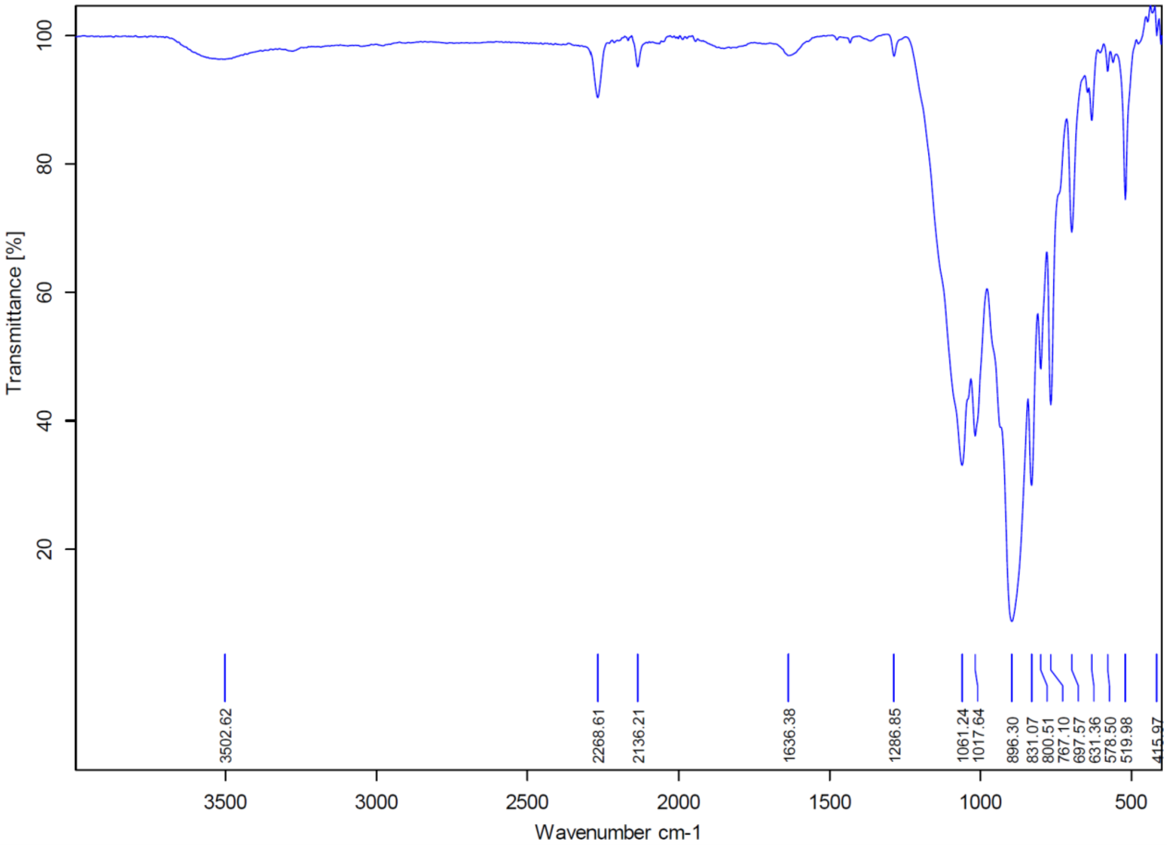

Figure S35. Solid-state-IR spectrum of **2**.

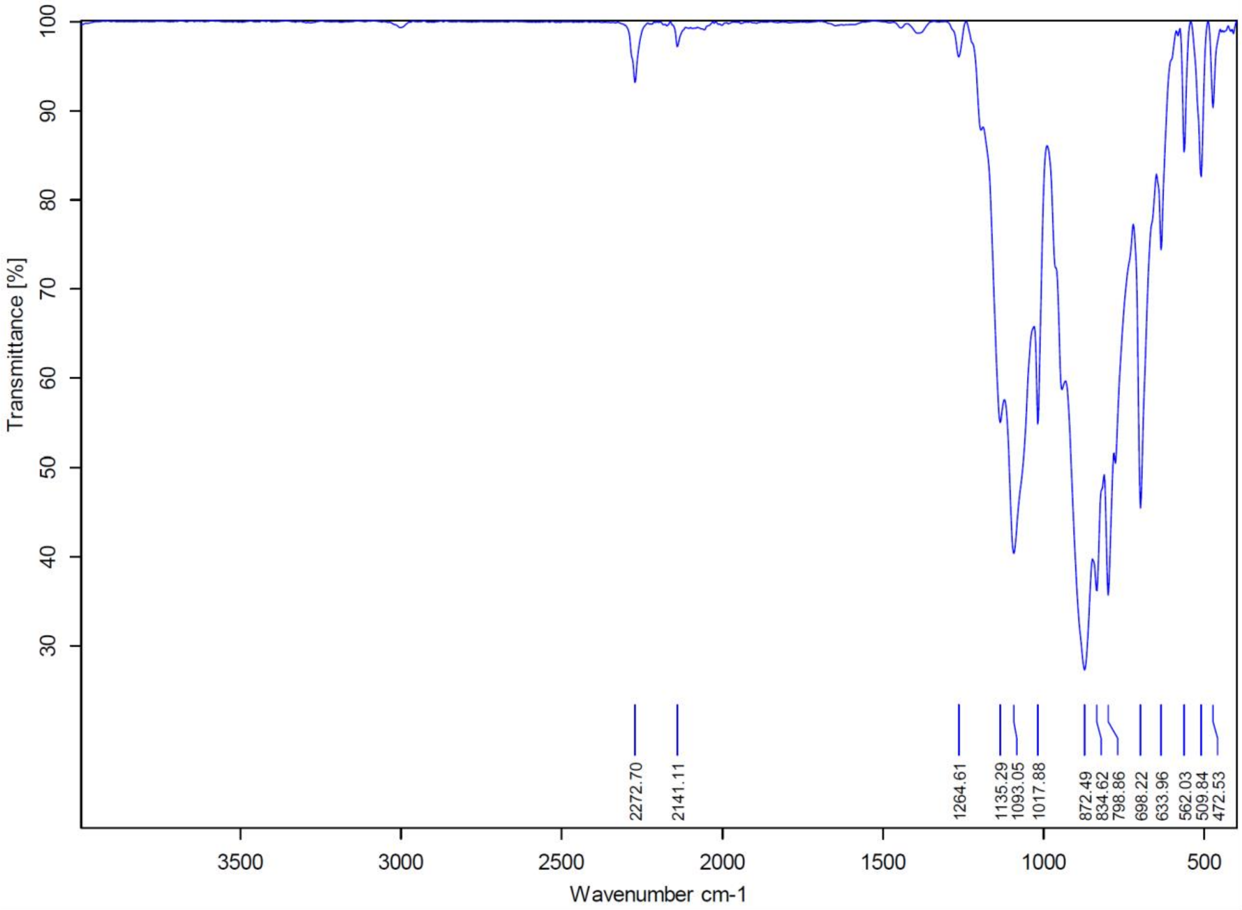

Figure S36. Solid state IR spectrum of a sample of **1-Cl** obtained by heating **2-Cl** for 1 hour in DMSO, removing the solvent and subliming the residual.

Heating compound **2** (200 mg) in DMSO (1 mL) to 150 °C for 2 h, followed by removal of all volatiles under reduced pressure, followed by sublimation gave a solid, the IR spectrum of which was qualitatively identical with that of pure **1** (Figures S34 and S36). Thus, the conversion of **2** into **1** via this pathway is possible.

Additional Computational Data

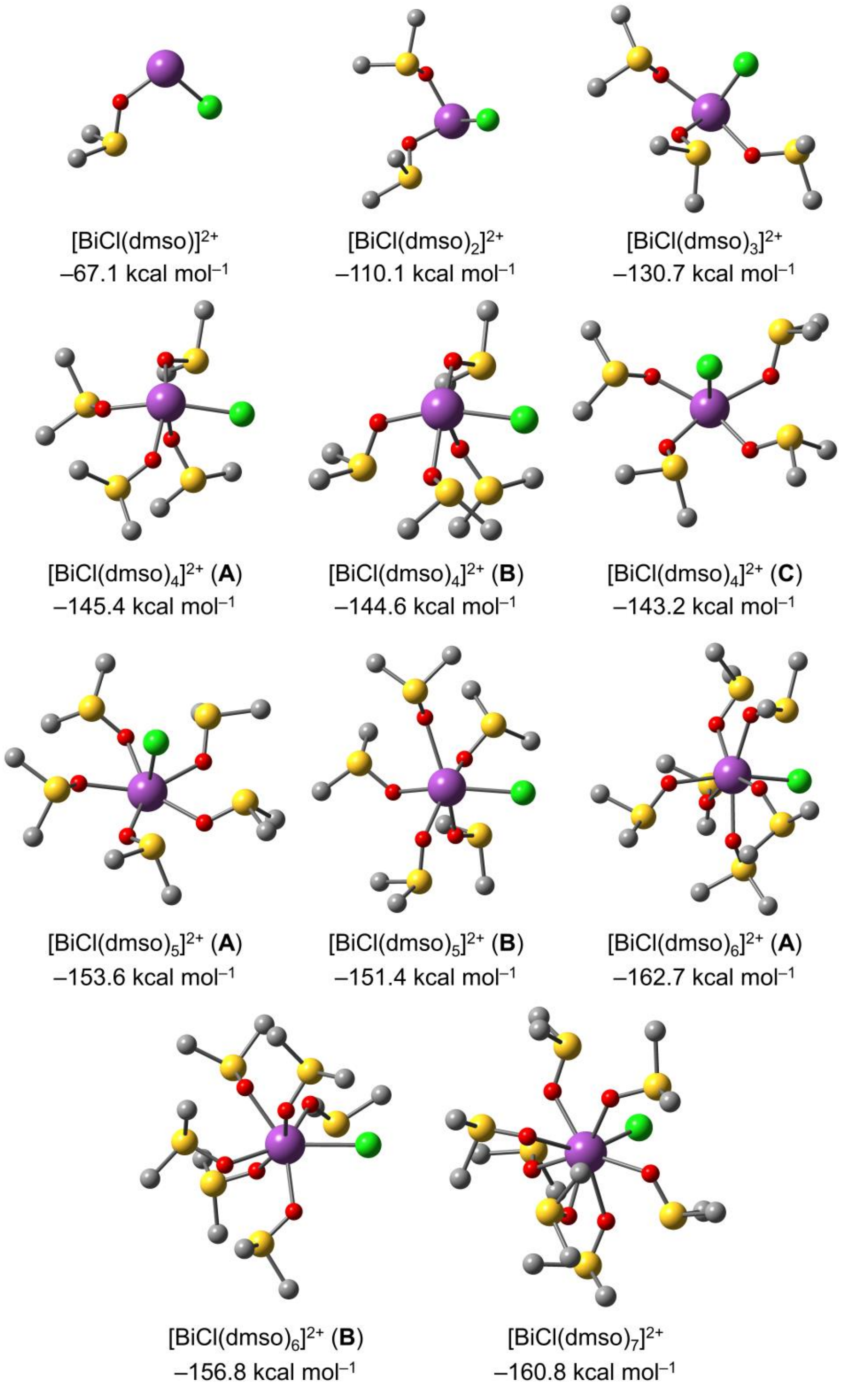

Figure S37. Three-dimensional structures of the $[BiCl(dmso)_n]^{2+}$ complexes (n = 1-7) computed in this study. The figure also displays the computed free energy of formation for each $[BiCl(dmso)_n]^{2+}$ system, which takes into account the incremental addition of n dmso ligands to $[BiCl]^{2+}$. Hydrogen atoms are omitted for clarity. Level of theory: PCM(DMSO)/B3LYP-D3/6-31G**;LANL2DZ(Bi).

Table S13. Energies of the computed systems. Electronic energies (*E*) values are corrected by zero-point energies. Gibbs free energies (*G*) include concentration correction. Level of theory: PCM(DMSO)/B3LYP-D3/6-31G**;LANL2DZ(Bi).

Entry	Compound	*E* [hartree]	*H* [hartree]	*G* [hartree]
1	$[BiCl]^{2+}$	-465.193652	-465.190031	-465.215714
2	dmso	-553.131539	-553.124946	-553.154381
3	$[BiCl(dmso)]^{2+}$	-1018.442252	-1018.431885	-1018.476952
4	$[BiCl(dmso)_2]^{2+}$	-1571.657206	-1571.640087	-1571.700004
5	$[BiCl(dmso)_3]^{2+}$	-2124.835370	-2124.811178	-2124.887068
6	$[BiCl(dmso)_4]^{2+}$ (**A**)	-2678.004640	-2677.973434	-2678.064902
7	$[BiCl(dmso)_4]^{2+}$ (**B**)	-2678.003790	-2677.972524	-2678.063729
8	$[BiCl(dmso)_4]^{2+}$ (**C**)	-2677.999749	-2677.968001	-2678.061519
9	$[BiCl(dmso)_5]^{2+}$ (**A**)	-3231.163996	-3231.126231	-3231.232357
10	$[BiCl(dmso)_5]^{2+}$ (**B**)	-3231.159624	-3231.120941	-3231.228909
11	$[BiCl(dmso)_6]^{2+}$ (**A**)	-3784.318999	-3784.272441	-3784.401278
12	$[BiCl(dmso)_6]^{2+}$ (**B**)	-3784.310952	-3784.264501	-3784.391856
13	$[BiCl(dmso)_7]^{2+}$	-4337.467754	-4337.415144	-4337.552621

Table S14. Total, Lewis and non-Lewis contributions in the NBO calculations of $[BiCl(dmso)_6]^{2+}$ (**A**), herein labelled as **X** and its model system in which the angle between the Cl atom and the dmso ligand *trans* to it was constrained to 180 degrees, herein labelled as **Y**.

System	*E* (X) [hatree]	*E* (Y) [hartree]	Δ*E* (Y–X) [kcal mol^{-1}]
Total	-3784.807654	-3784.804744	1.8
Lewis	-3782.956764	-3782.955317	0.9
non-Lewis	-1.850890	-1.849427	0.9

Table S15. NBO second-order perturbation energies, $E^{(2)}$, of $[BiCl(dmso)_6]^{2+}$ (**A**), herein labelled as **X**, its model system in which the angle between the Cl atom and the dmso ligand *trans* to it was constrained to 180 degrees, herein labelled as **Y**, and their energy difference (**Y**–**X**). Energy values are in kcal mol^{-1}. The predominant factor contributing to the destabilization of **Y** in comparison to **X** arises from a LP(O)→LV(Bi) donor-acceptor interaction, the corresponding orbitals herein labelled as LP (3) O 9 and LV (2) Bi 6 (from unit 6 to unit 3, $\Delta E^{(2)}$ = -6.34 kcal mol^{-1}). LP: valence lone pair; LV: lone vacant orbital. Corresponding NBO occupancies: 1.86 e$^-$; 0.31 e$^-$.

donor NBO	acceptor NBO	$E^{(2)}$ (X)	$E^{(2)}$ (Y)	$\Delta E^{(2)}$ (Y–X)
within unit 1				
71. LP (1) O 34	142. BD*(1) C 1- S 39	0.94	1.01	0.07
71. LP (1) O 34	427. RY (2) S 39	1.96	2.03	0.07
72. LP (2) O 34	142. BD*(1) C 1- S 39	0.90	0.72	-0.18
72. LP (2) O 34	165. BD*(1) C 21- H 24	0.74	10.43	9.69
72. LP (2) O 34	166. BD*(1) C 21- S 39	10.92	5.44	-5.48
72. LP (2) O 34	426. RY (1) S 39	5.76	0.67	-5.09
72. LP (2) O 34	430. RY (5) S 39	0.83	0.70	-0.13
72. LP (2) O 34	433. RY (8) S 39	0.51	0.52	0.01
73. LP (3) O 34	139. BD*(1) C 1- H 2	0.55	0.58	0.03
73. LP (3) O 34	142. BD*(1) C 1- S 39	6.88	7.29	0.41
73. LP (3) O 34	427. RY (2) S 39	4.50	0.72	-3.78
73. LP (3) O 34	430. RY (5) S 39	0.52	4.21	3.69
74. LP (1) S 39	140. BD*(1) C 1- H 3	1.97	0.66	-1.31

74. LP (1) S 39	164. BD*(1) C 21- H 23	1.83	1.97	0.14
82. BD (1) C 1- H 2	173. BD*(1) O 34- S 39	2.44	1.83	-0.61
83. BD (1) C 1- H 3	173. BD*(1) O 34- S 39	0.61	2.44	1.83
84. BD (1) C 1- H 4	166. BD*(1) C 21- S 39	1.50	0.61	-0.89
84. BD (1) C 1- H 4	429. RY (4) S 39	0.81	1.50	0.69
85. BD (1) C 1- S 39	163. BD*(1) C 21- H 22	1.25	0.81	-0.44
85. BD (1) C 1- S 39	166. BD*(1) C 21- S 39	0.59	1.25	0.66
85. BD (1) C 1- S 39	173. BD*(1) O 34- S 39	0.67	0.59	-0.08
85. BD (1) C 1- S 39	324. RY (1) C 21	0.78	0.67	-0.11
85. BD (1) C 1- S 39	396. RY (1) O 34	0.73	0.79	0.06
106. BD (1) C 21- H 22	142. BD*(1) C 1- S 39	1.52	0.73	-0.79
106. BD (1) C 21- H 22	429. RY (4) S 39	1.02	1.52	0.50
107. BD (1) C 21- H 23	173. BD*(1) O 34- S 39	0.65	1.02	0.37
108. BD (1) C 21- H 24	173. BD*(1) O 34- S 39	2.43	0.65	-1.78
109. BD (1) C 21- S 39	141. BD*(1) C 1- H 4	1.34	2.43	1.09
109. BD (1) C 21- S 39	142. BD*(1) C 1- S 39	0.56	1.34	0.78
109. BD (1) C 21- S 39	173. BD*(1) O 34- S 39	0.85	0.55	-0.30
109. BD (1) C 21- S 39	194. RY (1) C 1	0.52	0.86	0.34
109. BD (1) C 21- S 39	396. RY (1) O 34	0.64	0.64	0.00
116. BD (1) O 34- S 39	139. BD*(1) C 1- H 2	0.75	0.75	0.00
116. BD (1) O 34- S 39	142. BD*(1) C 1- S 39	1.03	1.03	0.00
116. BD (1) O 34- S 39	165. BD*(1) C 21- H 24	0.75	0.76	0.01
116. BD (1) O 34- S 39	166. BD*(1) C 21- S 39	1.03	1.03	0.00
116. BD (1) O 34- S 39	195. RY (2) C 1	0.56	0.55	-0.01
116. BD (1) O 34- S 39	325. RY (2) C 21	0.60	0.59	-0.01

from unit 1 to unit 2
None above threshold

from unit 1 to unit 3

71. LP (1) O 34	137. LV (1)Bi 6	2.64	3.30	0.66
71. LP (1) O 34	138. LV (2)Bi 6	13.43	12.29	-1.14
71. LP (1) O 34	146. BD*(1)Bi 6-Cl 14	0.13	0.27	0.14
71. LP (1) O 34	225. RY (2)Bi 6	0.00	0.18	0.18
71. LP (1) O 34	226. RY (3)Bi 6	2.04	1.92	-0.12
72. LP (2) O 34	137. LV (1)Bi 6	0.44	1.73	1.29
72. LP (2) O 34	138. LV (2)Bi 6	7.12	10.24	3.12
72. LP (2) O 34	146. BD*(1)Bi 6-Cl 14	2.89	2.91	0.02
72. LP (2) O 34	224. RY (1)Bi 6	0.11	0.13	0.02
72. LP (2) O 34	226. RY (3)Bi 6	0.09	0.08	-0.01
72. LP (2) O 34	227. RY (4)Bi 6	0.05	0.09	0.04
72. LP (2) O 34	276. RY (1)Cl 14	0.05	0.00	-0.05
73. LP (3) O 34	137. LV (1)Bi 6	17.89	20.82	2.93
73. LP (3) O 34	138. LV (2)Bi 6	30.26	25.01	-5.25
73. LP (3) O 34	146. BD*(1)Bi 6-Cl 14	0.39	0.00	-0.39
73. LP (3) O 34	226. RY (3)Bi 6	0.15	0.17	0.02
73. LP (3) O 34	227. RY (4)Bi 6	0.86	0.80	-0.06
73. LP (3) O 34	276. RY (1)Cl 14	0.11	0.07	-0.04
74. LP (1) S 39	137. LV (1)Bi 6	1.51	1.65	0.14
74. LP (1) S 39	138. LV (2)Bi 6	0.64	0.59	-0.05

74. LP (1) S 39	146. BD*(1)Bi 6-Cl 14	0.14	0.12	-0.02
74. LP (1) S 39	224. RY (1)Bi 6	0.06	0.07	0.01
74. LP (1) S 39	225. RY (2)Bi 6	0.06	0.43	0.37
74. LP (1) S 39	226. RY (3)Bi 6	0.36	0.08	-0.28
85. BD (1) C 1- S 39	137. LV (1)Bi 6	0.07	0.12	0.05
85. BD (1) C 1- S 39	138. LV (2)Bi 6	0.14	0.07	-0.07
106. BD (1) C 21- H 22	137. LV (1)Bi 6	0.07	0.07	0.00
109. BD (1) C 21- S 39	137. LV (1)Bi 6	0.06	0.09	0.03
109. BD (1) C 21- S 39	138. LV (2)Bi 6	0.09	1.30	1.21
116. BD (1) O 34- S 39	137. LV (1)Bi 6	1.19	0.85	-0.34
116. BD (1) O 34- S 39	138. LV (2)Bi 6	0.99	0.06	-0.93
116. BD (1) O 34- S 39	146. BD*(1)Bi 6-Cl 14	0.07	0.05	-0.02
116. BD (1) O 34- S 39	226. RY (3)Bi 6	0.20	0.17	-0.03
from unit 1 to unit 4				
None above threshold				
from unit 1 to unit 5				
72. LP (2) O 34	152. BD*(1) S 8- O 40	0.05	0.00	-0.05
73. LP (3) O 34	152. BD*(1) S 8- O 40	0.10	0.11	0.01
73. LP (3) O 34	437. RY (3) O 40	0.09	0.10	0.01
74. LP (1) S 39	152. BD*(1) S 8- O 40	0.07	0.07	0.00
74. LP (1) S 39	162. BD*(1) C 17- H 20	0.11	0.11	0.00
106. BD (1) C 21- H 22	152. BD*(1) S 8- O 40	0.05	0.05	0.00
from unit 1 to unit 6				
71. LP (1) O 34	181. BD*(1) S 45- C 59	0.28	0.00	-0.28
72. LP (2) O 34	181. BD*(1) S 45- C 59	0.14	0.00	-0.14
107. BD (1) C 21- H 23	190. BD*(1) C 55- H 58	0.06	0.00	-0.06
from unit 1 to unit 7				
71. LP (1) O 34	157. BD*(1) S 15- O 16	0.12	0.12	0.00
71. LP (1) O 34	159. BD*(1) S 15- C 51	0.64	0.64	0.00
71. LP (1) O 34	170. BD*(1) C 30- H 31	0.54	0.57	0.03
72. LP (2) O 34	157. BD*(1) S 15- O 16	0.24	0.24	0.00
72. LP (2) O 34	159. BD*(1) S 15- C 51	1.41	1.35	-0.06
72. LP (2) O 34	285. RY (1) S 15	0.05	0.00	-0.05
72. LP (2) O 34	287. RY (3) S 15	0.05	0.00	-0.05
72. LP (2) O 34	289. RY (5) S 15	0.05	0.00	-0.05
73. LP (3) O 34	170. BD*(1) C 30- H 31	0.22	0.22	0.00
83. BD (1) C 1- H 3	170. BD*(1) C 30- H 31	0.07	0.07	0.00
85. BD (1) C 1- S 39	291. RY (7) S 15	0.07	0.07	0.00
from unit 2 to unit 1				
None above threshold				
within unit 2				
54. LP (1) S 5	175. BD*(1) C 35- H 37	1.98	1.98	0.00
54. LP (1) S 5	179. BD*(1) C 41- H 44	1.89	1.89	0.00
79. LP (1) O 46	143. BD*(1) S 5- C 35	0.75	0.73	-0.02

79. LP (1) O 46	216. RY (2) S 5	1.76	1.75	-0.01
80. LP (2) O 46	144. BD*(1) S 5- C 41	9.85	10.85	1.00
80. LP (2) O 46	177. BD*(1) C 41- H 42	0.65	0.70	0.05
80. LP (2) O 46	215. RY (1) S 5	4.58	5.33	0.75
80. LP (2) O 46	216. RY (2) S 5	1.82	1.17	-0.65
80. LP (2) O 46	219. RY (5) S 5	0.00	0.57	0.57
80. LP (2) O 46	222. RY (8) S 5	0.63	0.62	-0.01
81. LP (3) O 46	143. BD*(1) S 5- C 35	9.59	9.25	-0.34
81. LP (3) O 46	144. BD*(1) S 5- C 41	1.38	0.59	-0.79
81. LP (3) O 46	174. BD*(1) C 35- H 36	0.68	0.66	-0.02
81. LP (3) O 46	215. RY (1) S 5	1.94	1.23	-0.71
81. LP (3) O 46	216. RY (2) S 5	4.11	4.68	0.57
81. LP (3) O 46	219. RY (5) S 5	0.88	0.75	-0.13
86. BD (1) S 5- C 35	144. BD*(1) S 5- C 41	0.61	0.61	0.00
86. BD (1) S 5- C 35	145. BD*(1) S 5- O 46	0.75	0.74	-0.01
86. BD (1) S 5- C 35	178. BD*(1) C 41- H 43	1.25	1.25	0.00
86. BD (1) S 5- C 35	444. RY (1) C 41	0.75	0.75	0.00
86. BD (1) S 5- C 35	474. RY (1) O 46	0.66	0.66	0.00
87. BD (1) S 5- C 41	143. BD*(1) S 5- C 35	0.57	0.57	0.00
87. BD (1) S 5- C 41	145. BD*(1) S 5- O 46	0.89	0.89	0.00
87. BD (1) S 5- C 41	176. BD*(1) C 35- H 38	1.34	1.34	0.00
87. BD (1) S 5- C 41	474. RY (1) O 46	0.64	0.64	0.00
88. BD (1) S 5- O 46	143. BD*(1) S 5- C 35	1.03	1.03	0.00
88. BD (1) S 5- O 46	144. BD*(1) S 5- C 41	1.03	1.04	0.01
88. BD (1) S 5- O 46	174. BD*(1) C 35- H 36	0.75	0.74	-0.01
88. BD (1) S 5- O 46	177. BD*(1) C 41- H 42	0.74	0.74	0.00
88. BD (1) S 5- O 46	406. RY (2) C 35	0.50	0.51	0.01
88. BD (1) S 5- O 46	445. RY (2) C 41	0.56	0.57	0.01
117. BD (1) C 35- H 36	145. BD*(1) S 5- O 46	2.36	2.36	0.00
118. BD (1) C 35- H 37	145. BD*(1) S 5- O 46	0.59	0.59	0.00
119. BD (1) C 35- H 38	144. BD*(1) S 5- C 41	1.47	1.47	0.00
119. BD (1) C 35- H 38	218. RY (4) S 5	0.82	0.82	0.00
120. BD (1) C 41- H 42	145. BD*(1) S 5- O 46	2.39	2.40	0.01
121. BD (1) C 41- H 43	143. BD*(1) S 5- C 35	1.50	1.50	0.00
121. BD (1) C 41- H 43	218. RY (4) S 5	1.00	1.00	0.00
122. BD (1) C 41- H 44	145. BD*(1) S 5- O 46	0.60	0.60	0.00
from unit 2 to unit 3				
54. LP (1) S 5	137. LV (1)Bi 6	0.20	0.28	0.08
54. LP (1) S 5	138. LV (2)Bi 6	1.79	1.65	-0.14
54. LP (1) S 5	146. BD*(1)Bi 6-Cl 14	0.07	0.08	0.01
54. LP (1) S 5	225. RY (2)Bi 6	0.00	0.10	0.10
54. LP (1) S 5	226. RY (3)Bi 6	0.36	0.30	-0.06
79. LP (1) O 46	137. LV (1)Bi 6	0.48	0.21	-0.27
79. LP (1) O 46	138. LV (2)Bi 6	13.56	14.38	0.82
79. LP (1) O 46	146. BD*(1)Bi 6-Cl 14	0.59	0.47	-0.12
79. LP (1) O 46	224. RY (1)Bi 6	0.15	0.34	0.19
79. LP (1) O 46	225. RY (2)Bi 6	0.79	1.17	0.38
79. LP (1) O 46	226. RY (3)Bi 6	1.23	0.67	-0.56
80. LP (2) O 46	137. LV (1)Bi 6	0.69	0.47	-0.22

80. LP (2) O 46	138. LV (2)Bi 6	18.54	13.94	-4.60
80. LP (2) O 46	146. BD*(1)Bi 6-Cl 14	1.48	1.79	0.31
80. LP (2) O 46	224. RY (1)Bi 6	0.08	0.00	-0.08
80. LP (2) O 46	225. RY (2)Bi 6	0.19	0.25	0.06
80. LP (2) O 46	226. RY (3)Bi 6	0.15	0.05	-0.10
80. LP (2) O 46	227. RY (4)Bi 6	0.13	0.08	-0.05
81. LP (3) O 46	137. LV (1)Bi 6	0.39	0.75	0.36
81. LP (3) O 46	138. LV (2)Bi 6	29.11	34.34	5.23
81. LP (3) O 46	146. BD*(1)Bi 6-Cl 14	0.32	0.00	-0.32
81. LP (3) O 46	224. RY (1)Bi 6	0.09	0.11	0.02
81. LP (3) O 46	225. RY (2)Bi 6	0.00	0.05	0.05
81. LP (3) O 46	226. RY (3)Bi 6	0.16	0.09	-0.07
81. LP (3) O 46	227. RY (4)Bi 6	0.33	0.42	0.09
86. BD (1) S 5- C 35	138. LV (2)Bi 6	0.16	0.17	0.01
87. BD (1) S 5- C 41	138. LV (2)Bi 6	0.13	0.12	-0.01
88. BD (1) S 5- O 46	137. LV (1)Bi 6	0.05	0.09	0.04
88. BD (1) S 5- O 46	138. LV (2)Bi 6	1.63	1.63	0.00
88. BD (1) S 5- O 46	225. RY (2)Bi 6	0.07	0.09	0.02
from unit 2 to unit 4				
54. LP (1) S 5	147. BD*(1) S 7- O 25	0.05	0.05	0.00
81. LP (3) O 46	147. BD*(1) S 7- O 25	0.17	0.15	-0.02
81. LP (3) O 46	348. RY (4) O 25	0.08	0.10	0.02
from unit 2 to unit 5				
79. LP (1) O 46	151. BD*(1) S 8- C 17	0.60	0.60	0.00
79. LP (1) O 46	152. BD*(1) S 8- O 40	0.10	0.10	0.00
79. LP (1) O 46	155. BD*(1) C 10- H 12	1.03	1.02	-0.01
80. LP (2) O 46	151. BD*(1) S 8- C 17	1.12	1.26	0.14
80. LP (2) O 46	152. BD*(1) S 8- O 40	0.19	0.20	0.01
80. LP (2) O 46	241. RY (5) S 8	0.00	0.05	0.05
81. LP (3) O 46	151. BD*(1) S 8- C 17	0.20	0.10	-0.10
81. LP (3) O 46	155. BD*(1) C 10- H 12	0.37	0.38	0.01
86. BD (1) S 5- C 35	243. RY (7) S 8	0.06	0.06	0.00
86. BD (1) S 5- C 35	268. RY (1) H 12	0.07	0.07	0.00
118. BD (1) C 35- H 37	155. BD*(1) C 10- H 12	0.06	0.06	0.00
from unit 2 to unit 6				
88. BD (1) S 5- O 46	470. RY (6) S 45	0.07	0.06	-0.01
from unit 2 to unit 7				
None above threshold				
from unit 3 to unit 1				
55. LP (1)Bi 6	173. BD*(1) O 34- S 39	1.29	1.43	0.14
55. LP (1)Bi 6	398. RY (3) O 34	0.12	0.16	0.04
55. LP (1)Bi 6	399. RY (4) O 34	0.06	0.00	-0.06
55. LP (1)Bi 6	431. RY (6) S 39	0.08	0.10	0.02
62. LP (2)Cl 14	166. BD*(1) C 21- S 39	0.18	0.18	0.00

from unit 3 to unit 2				
55. LP (1)Bi 6	145. BD*(1) S 5- O 46	1.30	1.21	-0.09
55. LP (1)Bi 6	220. RY (6) S 5	0.07	0.05	-0.02
55. LP (1)Bi 6	477. RY (4) O 46	0.08	0.00	-0.08
55. LP (1)Bi 6	478. RY (5) O 46	0.00	0.05	0.05
62. LP (2)Cl 14	144. BD*(1) S 5- C 41	0.06	0.06	0.00
within unit 3				
55. LP (1)Bi 6	276. RY (1)Cl 14	0.93	0.91	-0.02
61. LP (1)Cl 14	224. RY (1)Bi 6	4.96	4.80	-0.16
62. LP (2)Cl 14	137. LV (1)Bi 6	4.93	6.18	1.25
62. LP (2)Cl 14	138. LV (2)Bi 6	2.80	1.58	-1.22
63. LP (3)Cl 14	137. LV (1)Bi 6	2.76	1.58	-1.18
63. LP (3)Cl 14	138. LV (2)Bi 6	4.89	6.02	1.13
from unit 3 to unit 4				
55. LP (1)Bi 6	147. BD*(1) S 7- O 25	1.45	1.40	-0.05
55. LP (1)Bi 6	233. RY (6) S 7	0.07	0.07	0.00
55. LP (1)Bi 6	347. RY (3) O 25	0.09	0.00	-0.09
55. LP (1)Bi 6	348. RY (4) O 25	0.00	0.08	0.08
62. LP (2)Cl 14	148. BD*(1) S 7- C 26	0.08	0.08	0.00
from unit 3 to unit 5				
55. LP (1)Bi 6	152. BD*(1) S 8- O 40	1.36	1.32	-0.04
55. LP (1)Bi 6	242. RY (6) S 8	0.08	0.07	-0.01
55. LP (1)Bi 6	437. RY (3) O 40	0.13	0.11	-0.02
63. LP (3)Cl 14	150. BD*(1) S 8- C 10	0.13	0.11	-0.02
from unit 3 to unit 6				
55. LP (1)Bi 6	153. BD*(1) O 9- S 45	1.79	1.74	-0.05
55. LP (1)Bi 6	248. RY (3) O 9	0.09	0.09	0.00
55. LP (1)Bi 6	470. RY (6) S 45	0.05	0.00	-0.05
61. LP (1)Cl 14	153. BD*(1) O 9- S 45	0.09	0.09	0.00
89. BD (1)Bi 6-Cl 14	153. BD*(1) O 9- S 45	0.21	0.20	-0.01
89. BD (1)Bi 6-Cl 14	246. RY (1) O 9	0.11	0.11	0.00
from unit 3 to unit 7				
55. LP (1)Bi 6	157. BD*(1) S 15- O 16	1.36	1.49	0.13
55. LP (1)Bi 6	290. RY (6) S 15	0.07	0.10	0.03
55. LP (1)Bi 6	296. RY (3) O 16	0.13	0.18	0.05
55. LP (1)Bi 6	297. RY (4) O 16	0.05	0.00	-0.05
63. LP (3)Cl 14	158. BD*(1) S 15- C 30	0.34	0.37	0.03
89. BD (1)Bi 6-Cl 14	158. BD*(1) S 15- C 30	0.07	0.07	0.00
from unit 4 to unit 1				
None above threshold				
from unit 4 to unit 2				
68. LP (1) O 25	143. BD*(1) S 5- C 35	0.56	0.56	0.00
68. LP (1) O 25	145. BD*(1) S 5- O 46	0.09	0.09	0.00

68. LP (1) O 25	178. BD*(1) C 41- H 43	0.95	0.95	0.00
69. LP (2) O 25	143. BD*(1) S 5- C 35	1.07	1.10	0.03
69. LP (2) O 25	145. BD*(1) S 5- O 46	0.17	0.17	0.00
70. LP (3) O 25	143. BD*(1) S 5- C 35	0.10	0.09	-0.01
70. LP (3) O 25	178. BD*(1) C 41- H 43	0.35	0.35	0.00
92. BD (1) S 7- C 47	221. RY (7) S 5	0.06	0.06	0.00
92. BD (1) S 7- C 47	457. RY (1) H 43	0.07	0.07	0.00
127. BD (1) C 47- H 50	178. BD*(1) C 41- H 43	0.07	0.07	0.00
from unit 4 to unit 3				
56. LP (1) S 7	137. LV (1)Bi 6	2.01	2.01	0.00
56. LP (1) S 7	146. BD*(1)Bi 6-Cl 14	0.08	0.08	0.00
56. LP (1) S 7	225. RY (2)Bi 6	0.29	0.18	-0.11
56. LP (1) S 7	226. RY (3)Bi 6	0.13	0.24	0.11
68. LP (1) O 25	137. LV (1)Bi 6	11.95	12.63	0.68
68. LP (1) O 25	138. LV (2)Bi 6	3.34	2.86	-0.48
68. LP (1) O 25	146. BD*(1)Bi 6-Cl 14	0.56	0.52	-0.04
68. LP (1) O 25	224. RY (1)Bi 6	0.12	0.16	0.04
68. LP (1) O 25	225. RY (2)Bi 6	0.63	0.26	-0.37
68. LP (1) O 25	226. RY (3)Bi 6	1.37	1.73	0.36
69. LP (2) O 25	137. LV (1)Bi 6	10.93	11.24	0.31
69. LP (2) O 25	138. LV (2)Bi 6	4.28	3.33	-0.95
69. LP (2) O 25	146. BD*(1)Bi 6-Cl 14	2.03	2.08	0.05
69. LP (2) O 25	224. RY (1)Bi 6	0.09	0.05	-0.04
69. LP (2) O 25	226. RY (3)Bi 6	0.26	0.28	0.02
69. LP (2) O 25	227. RY (4)Bi 6	0.10	0.09	-0.01
70. LP (3) O 25	137. LV (1)Bi 6	36.56	37.80	1.24
70. LP (3) O 25	138. LV (2)Bi 6	1.30	0.92	-0.38
70. LP (3) O 25	224. RY (1)Bi 6	0.07	0.08	0.01
70. LP (3) O 25	225. RY (2)Bi 6	0.08	0.06	-0.02
70. LP (3) O 25	226. RY (3)Bi 6	0.08	0.09	0.01
70. LP (3) O 25	227. RY (4)Bi 6	0.54	0.57	0.03
70. LP (3) O 25	276. RY (1)Cl 14	0.08	0.06	-0.02
90. BD (1) S 7- O 25	137. LV (1)Bi 6	1.84	1.89	0.05
90. BD (1) S 7- O 25	226. RY (3)Bi 6	0.12	0.14	0.02
91. BD (1) S 7- C 26	137. LV (1)Bi 6	0.13	0.13	0.00
92. BD (1) S 7- C 47	137. LV (1)Bi 6	0.17	0.17	0.00
within unit 4				
56. LP (1) S 7	169. BD*(1) C 26- H 29	1.86	1.86	0.00
56. LP (1) S 7	184. BD*(1) C 47- H 50	1.97	1.98	0.01
68. LP (1) O 25	149. BD*(1) S 7- C 47	0.89	0.89	0.00
68. LP (1) O 25	229. RY (2) S 7	1.82	1.85	0.03
69. LP (2) O 25	148. BD*(1) S 7- C 26	10.47	10.56	0.09
69. LP (2) O 25	167. BD*(1) C 26- H 27	0.67	0.68	0.01
69. LP (2) O 25	228. RY (1) S 7	5.30	5.36	0.06
69. LP (2) O 25	229. RY (2) S 7	1.09	1.02	-0.07
69. LP (2) O 25	232. RY (5) S 7	0.55	0.58	0.03
69. LP (2) O 25	235. RY (8) S 7	0.62	0.60	-0.02
70. LP (3) O 25	148. BD*(1) S 7- C 26	0.80	0.70	-0.10

70. LP (3) O 25	149. BD*(1) S 7- C 47	8.70	8.63	-0.07
70. LP (3) O 25	183. BD*(1) C 47- H 49	0.64	0.63	-0.01
70. LP (3) O 25	228. RY (1) S 7	1.19	1.12	-0.07
70. LP (3) O 25	229. RY (2) S 7	4.37	4.40	0.03
70. LP (3) O 25	232. RY (5) S 7	0.71	0.68	-0.03
90. BD (1) S 7- O 25	148. BD*(1) S 7- C 26	1.04	1.04	0.00
90. BD (1) S 7- O 25	149. BD*(1) S 7- C 47	1.03	1.04	0.01
90. BD (1) S 7- O 25	167. BD*(1) C 26- H 27	0.74	0.74	0.00
90. BD (1) S 7- O 25	183. BD*(1) C 47- H 49	0.74	0.74	0.00
90. BD (1) S 7- O 25	355. RY (2) C 26	0.54	0.54	0.00
90. BD (1) S 7- O 25	484. RY (2) C 47	0.53	0.53	0.00
91. BD (1) S 7- C 26	147. BD*(1) S 7- O 25	0.89	0.89	0.00
91. BD (1) S 7- C 26	149. BD*(1) S 7- C 47	0.56	0.56	0.00
91. BD (1) S 7- C 26	182. BD*(1) C 47- H 48	1.33	1.33	0.00
91. BD (1) S 7- C 26	345. RY (1) O 25	0.60	0.60	0.00
92. BD (1) S 7- C 47	147. BD*(1) S 7- O 25	0.71	0.71	0.00
92. BD (1) S 7- C 47	148. BD*(1) S 7- C 26	0.60	0.60	0.00
92. BD (1) S 7- C 47	168. BD*(1) C 26- H 28	1.26	1.26	0.00
92. BD (1) S 7- C 47	345. RY (1) O 25	0.70	0.70	0.00
92. BD (1) S 7- C 47	354. RY (1) C 26	0.77	0.76	-0.01
110. BD (1) C 26- H 27	147. BD*(1) S 7- O 25	2.40	2.40	0.00
111. BD (1) C 26- H 28	149. BD*(1) S 7- C 47	1.50	1.51	0.01
111. BD (1) C 26- H 28	231. RY (4) S 7	1.02	1.02	0.00
112. BD (1) C 26- H 29	147. BD*(1) S 7- O 25	0.66	0.66	0.00
125. BD (1) C 47- H 48	148. BD*(1) S 7- C 26	1.50	1.49	-0.01
125. BD (1) C 47- H 48	231. RY (4) S 7	0.79	0.79	0.00
126. BD (1) C 47- H 49	147. BD*(1) S 7- O 25	2.41	2.41	0.00
127. BD (1) C 47- H 50	147. BD*(1) S 7- O 25	0.58	0.58	0.00
from unit 4 to unit 5				
None above threshold				
from unit 4 to unit 6				
111. BD (1) C 26- H 28	193. BD*(1) C 59- H 62	0.06	0.00	-0.06
from unit 4 to unit 7				
56. LP (1) S 7	187. BD*(1) C 51- H 54	0.19	0.19	0.00
70. LP (3) O 25	157. BD*(1) S 15- O 16	0.15	0.14	-0.01
70. LP (3) O 25	295. RY (2) O 16	0.00	0.05	0.05
70. LP (3) O 25	296. RY (3) O 16	0.12	0.08	-0.04
111. BD (1) C 26- H 28	157. BD*(1) S 15- O 16	0.05	0.05	0.00
from unit 5 to unit 1				
75. LP (1) O 40	142. BD*(1) C 1- S 39	0.70	0.69	-0.01
75. LP (1) O 40	163. BD*(1) C 21- H 22	1.02	1.02	0.00
75. LP (1) O 40	173. BD*(1) O 34- S 39	0.11	0.11	0.00
76. LP (2) O 40	142. BD*(1) C 1- S 39	1.50	1.55	0.05
76. LP (2) O 40	173. BD*(1) O 34- S 39	0.25	0.25	0.00
76. LP (2) O 40	399. RY (4) O 34	0.05	0.00	-0.05
76. LP (2) O 40	427. RY (2) S 39	0.05	0.06	0.01

76. LP (2) O 40	428. RY (3) S 39	0.06	0.06	0.00
76. LP (2) O 40	430. RY (5) S 39	0.05	0.05	0.00
76. LP (2) O 40	432. RY (7) S 39	0.05	0.05	0.00
77. LP (3) O 40	142. BD*(1) C 1- S 39	0.09	0.06	-0.03
77. LP (3) O 40	163. BD*(1) C 21- H 22	0.30	0.30	0.00
94. BD (1) S 8- C 17	333. RY (1) H 22	0.05	0.05	0.00
94. BD (1) S 8- C 17	432. RY (7) S 39	0.08	0.08	0.00
from unit 5 to unit 2				
57. LP (1) S 8	145. BD*(1) S 5- O 46	0.06	0.06	0.00
57. LP (1) S 8	176. BD*(1) C 35- H 38	0.06	0.06	0.00
77. LP (3) O 40	145. BD*(1) S 5- O 46	0.12	0.12	0.00
77. LP (3) O 40	476. RY (3) O 46	0.00	0.10	0.10
77. LP (3) O 40	477. RY (4) O 46	0.11	0.00	-0.11
95. BD (1) S 8- O 40	220. RY (6) S 5	0.05	0.00	-0.05
from unit 5 to unit 3				
57. LP (1) S 8	137. LV (1)Bi 6	1.37	1.25	-0.12
57. LP (1) S 8	138. LV (2)Bi 6	0.83	0.90	0.07
57. LP (1) S 8	146. BD*(1)Bi 6-Cl 14	0.10	0.11	0.01
57. LP (1) S 8	225. RY (2)Bi 6	0.45	0.45	0.00
75. LP (1) O 40	137. LV (1)Bi 6	14.47	14.36	-0.11
75. LP (1) O 40	138. LV (2)Bi 6	0.18	0.54	0.36
75. LP (1) O 40	146. BD*(1)Bi 6-Cl 14	0.49	0.47	-0.02
75. LP (1) O 40	224. RY (1)Bi 6	0.06	0.10	0.04
75. LP (1) O 40	225. RY (2)Bi 6	1.81	1.34	-0.47
75. LP (1) O 40	226. RY (3)Bi 6	0.24	0.69	0.45
76. LP (2) O 40	137. LV (1)Bi 6	14.78	13.18	-1.60
76. LP (2) O 40	138. LV (2)Bi 6	0.00	0.07	0.07
76. LP (2) O 40	146. BD*(1)Bi 6-Cl 14	2.12	2.10	-0.02
76. LP (2) O 40	224. RY (1)Bi 6	0.10	0.09	-0.01
76. LP (2) O 40	225. RY (2)Bi 6	0.13	0.09	-0.04
76. LP (2) O 40	226. RY (3)Bi 6	0.00	0.09	0.09
76. LP (2) O 40	227. RY (4)Bi 6	0.11	0.08	-0.03
77. LP (3) O 40	137. LV (1)Bi 6	33.47	32.80	-0.67
77. LP (3) O 40	138. LV (2)Bi 6	4.76	7.19	2.43
77. LP (3) O 40	224. RY (1)Bi 6	0.06	0.08	0.02
77. LP (3) O 40	225. RY (2)Bi 6	0.16	0.11	-0.05
77. LP (3) O 40	227. RY (4)Bi 6	0.56	0.60	0.04
77. LP (3) O 40	276. RY (1)Cl 14	0.06	0.05	-0.01
93. BD (1) S 8- C 10	137. LV (1)Bi 6	0.13	0.12	-0.01
93. BD (1) S 8- C 10	224. RY (1)Bi 6	0.00	0.06	0.06
94. BD (1) S 8- C 17	137. LV (1)Bi 6	0.17	0.16	-0.01
95. BD (1) S 8- O 40	137. LV (1)Bi 6	1.51	1.43	-0.08
95. BD (1) S 8- O 40	138. LV (2)Bi 6	0.45	0.56	0.11
95. BD (1) S 8- O 40	225. RY (2)Bi 6	0.09	0.06	-0.03
95. BD (1) S 8- O 40	226. RY (3)Bi 6	0.12	0.14	0.02

from unit 5 to unit 4
None above threshold

within unit 5				
57. LP (1) S 8	156. BD*(1) C 10- H 13	1.86	1.86	0.00
57. LP (1) S 8	161. BD*(1) C 17- H 19	1.99	1.99	0.00
75. LP (1) O 40	151. BD*(1) S 8- C 17	0.80	0.77	-0.03
75. LP (1) O 40	238. RY (2) S 8	1.87	1.85	-0.02
76. LP (2) O 40	150. BD*(1) S 8- C 10	10.52	10.75	0.23
76. LP (2) O 40	154. BD*(1) C 10- H 11	0.70	0.71	0.01
76. LP (2) O 40	237. RY (1) S 8	5.14	5.39	0.25
76. LP (2) O 40	238. RY (2) S 8	1.06	0.84	-0.22
76. LP (2) O 40	241. RY (5) S 8	0.66	0.70	0.04
76. LP (2) O 40	244. RY (8) S 8	0.56	0.57	0.01
77. LP (3) O 40	151. BD*(1) S 8- C 17	8.41	8.25	-0.16
77. LP (3) O 40	160. BD*(1) C 17- H 18	0.63	0.62	-0.01
77. LP (3) O 40	237. RY (1) S 8	1.12	0.90	-0.22
77. LP (3) O 40	238. RY (2) S 8	4.39	4.61	0.22
77. LP (3) O 40	241. RY (5) S 8	0.75	0.67	-0.08
93. BD (1) S 8- C 10	151. BD*(1) S 8- C 17	0.57	0.57	0.00
93. BD (1) S 8- C 10	152. BD*(1) S 8- O 40	0.86	0.86	0.00
93. BD (1) S 8- C 10	162. BD*(1) C 17- H 20	1.33	1.33	0.00
93. BD (1) S 8- C 10	435. RY (1) O 40	0.66	0.66	0.00
94. BD (1) S 8- C 17	150. BD*(1) S 8- C 10	0.61	0.61	0.00
94. BD (1) S 8- C 17	152. BD*(1) S 8- O 40	0.72	0.72	0.00
94. BD (1) S 8- C 17	155. BD*(1) C 10- H 12	1.25	1.25	0.00
94. BD (1) S 8- C 17	255. RY (1) C 10	0.78	0.78	0.00
94. BD (1) S 8- C 17	435. RY (1) O 40	0.66	0.67	0.01
95. BD (1) S 8- O 40	150. BD*(1) S 8- C 10	1.03	1.03	0.00
95. BD (1) S 8- O 40	151. BD*(1) S 8- C 17	1.03	1.03	0.00
95. BD (1) S 8- O 40	154. BD*(1) C 10- H 11	0.74	0.74	0.00
95. BD (1) S 8- O 40	160. BD*(1) C 17- H 18	0.75	0.75	0.00
95. BD (1) S 8- O 40	256. RY (2) C 10	0.57	0.58	0.01
95. BD (1) S 8- O 40	304. RY (2) C 17	0.51	0.52	0.01
97. BD (1) C 10- H 11	152. BD*(1) S 8- O 40	2.43	2.43	0.00
98. BD (1) C 10- H 12	151. BD*(1) S 8- C 17	1.52	1.52	0.00
98. BD (1) C 10- H 12	240. RY (4) S 8	1.02	1.02	0.00
99. BD (1) C 10- H 13	152. BD*(1) S 8- O 40	0.64	0.64	0.00
103. BD (1) C 17- H 18	152. BD*(1) S 8- O 40	2.41	2.41	0.00
104. BD (1) C 17- H 19	152. BD*(1) S 8- O 40	0.57	0.57	0.00
105. BD (1) C 17- H 20	150. BD*(1) S 8- C 10	1.48	1.48	0.00
105. BD (1) C 17- H 20	240. RY (4) S 8	0.81	0.82	0.01
from unit 5 to unit 6				
95. BD (1) S 8- O 40	470. RY (6) S 45	0.07	0.07	0.00
from unit 5 to unit 7				
None above threshold				
from unit 6 to unit 1				
78. LP (1) S 45	173. BD*(1) O 34- S 39	0.05	0.00	-0.05

from unit 6 to unit 2				
96. BD (1) O 9- S 45	220. RY (6) S 5	0.10	0.09	-0.01
from unit 6 to unit 3				
58. LP (1) O 9	138. LV (2)Bi 6	0.00	1.39	1.39
58. LP (1) O 9	146. BD*(1)Bi 6-Cl 14	12.01	11.54	-0.47
58. LP (1) O 9	224. RY (1)Bi 6	3.26	3.18	-0.08
59. LP (2) O 9	137. LV (1)Bi 6	5.93	5.96	0.03
59. LP (2) O 9	138. LV (2)Bi 6	0.10	0.00	-0.10
59. LP (2) O 9	146. BD*(1)Bi 6-Cl 14	0.83	1.12	0.29
59. LP (2) O 9	225. RY (2)Bi 6	0.16	0.10	-0.06
59. LP (2) O 9	226. RY (3)Bi 6	0.08	0.14	0.06
59. LP (2) O 9	277. RY (2)Cl 14	0.05	0.05	0.00
60. LP (3) O 9	137. LV (1)Bi 6	0.09	0.06	-0.03
60. LP (3) O 9	138. LV (2)Bi 6	6.67	0.33	-6.34
60. LP (3) O 9	146. BD*(1)Bi 6-Cl 14	29.89	33.57	3.68
60. LP (3) O 9	224. RY (1)Bi 6	0.89	0.95	0.06
60. LP (3) O 9	225. RY (2)Bi 6	0.00	0.06	0.06
60. LP (3) O 9	227. RY (4)Bi 6	0.80	0.79	-0.01
60. LP (3) O 9	279. RY (4)Cl 14	0.06	0.08	0.02
78. LP (1) S 45	138. LV (2)Bi 6	1.34	0.67	-0.67
78. LP (1) S 45	146. BD*(1)Bi 6-Cl 14	0.99	1.66	0.67
78. LP (1) S 45	224. RY (1)Bi 6	0.50	0.62	0.12
78. LP (1) S 45	225. RY (2)Bi 6	0.06	0.09	0.03
78. LP (1) S 45	226. RY (3)Bi 6	0.16	0.08	-0.08
96. BD (1) O 9- S 45	138. LV (2)Bi 6	0.62	0.14	-0.48
96. BD (1) O 9- S 45	146. BD*(1)Bi 6-Cl 14	1.11	1.55	0.44
96. BD (1) O 9- S 45	224. RY (1)Bi 6	0.27	0.30	0.03
123. BD (1) S 45- C 55	146. BD*(1)Bi 6-Cl 14	0.12	0.12	0.00
123. BD (1) S 45- C 55	225. RY (2)Bi 6	0.06	0.09	0.03
124. BD (1) S 45- C 59	146. BD*(1)Bi 6-Cl 14	0.12	0.12	0.00
124. BD (1) S 45- C 59	226. RY (3)Bi 6	0.00	0.08	0.08
from unit 6 to unit 4				
None above threshold				
from unit 6 to unit 5				
None above threshold				
within unit 6				
58. LP (1) O 9	180. BD*(1) S 45- C 55	0.58	0.54	-0.04
58. LP (1) O 9	181. BD*(1) S 45- C 59	0.52	0.00	-0.52
58. LP (1) O 9	466. RY (2) S 45	2.13	2.05	-0.08
59. LP (2) O 9	180. BD*(1) S 45- C 55	6.16	5.79	-0.37
59. LP (2) O 9	181. BD*(1) S 45- C 59	9.69	9.81	0.12
59. LP (2) O 9	191. BD*(1) C 59- H 60	0.62	0.64	0.02
59. LP (2) O 9	465. RY (1) S 45	6.46	6.48	0.02
59. LP (2) O 9	469. RY (5) S 45	2.44	2.56	0.12
60. LP (3) O 9	180. BD*(1) S 45- C 55	4.19	4.45	0.26
60. LP (3) O 9	181. BD*(1) S 45- C 59	1.61	1.45	-0.16

60. LP (3) O 9	466. RY (2) S 45	5.18	5.47	0.29
78. LP (1) S 45	188. BD*(1) C 55- H 56	1.93	1.91	-0.02
78. LP (1) S 45	192. BD*(1) C 59- H 61	1.93	1.90	-0.03
96. BD (1) O 9- S 45	180. BD*(1) S 45- C 55	1.06	1.03	-0.03
96. BD (1) O 9- S 45	181. BD*(1) S 45- C 59	1.06	1.04	-0.02
96. BD (1) O 9- S 45	189. BD*(1) C 55- H 57	0.72	0.72	0.00
96. BD (1) O 9- S 45	191. BD*(1) C 59- H 60	0.71	0.71	0.00
96. BD (1) O 9- S 45	526. RY (2) C 55	0.57	0.62	0.05
96. BD (1) O 9- S 45	547. RY (2) C 59	0.55	0.61	0.06
123. BD (1) S 45- C 55	153. BD*(1) O 9- S 45	0.79	0.78	-0.01
123. BD (1) S 45- C 55	181. BD*(1) S 45- C 59	0.60	0.62	0.02
123. BD (1) S 45- C 55	193. BD*(1) C 59- H 62	1.33	1.31	-0.02
123. BD (1) S 45- C 55	246. RY (1) O 9	0.64	0.62	-0.02
123. BD (1) S 45- C 55	546. RY (1) C 59	0.71	0.74	0.03
124. BD (1) S 45- C 59	153. BD*(1) O 9- S 45	0.81	0.80	-0.01
124. BD (1) S 45- C 59	180. BD*(1) S 45- C 55	0.60	0.62	0.02
124. BD (1) S 45- C 59	190. BD*(1) C 55- H 58	1.33	1.32	-0.01
124. BD (1) S 45- C 59	246. RY (1) O 9	0.59	0.60	0.01
124. BD (1) S 45- C 59	525. RY (1) C 55	0.71	0.73	0.02
131. BD (1) C 55- H 56	153. BD*(1) O 9- S 45	0.64	0.64	0.00
132. BD (1) C 55- H 57	153. BD*(1) O 9- S 45	2.44	2.38	-0.06
133. BD (1) C 55- H 58	181. BD*(1) S 45- C 59	1.53	1.49	-0.04
133. BD (1) C 55- H 58	468. RY (4) S 45	0.55	0.90	0.35
134. BD (1) C 59- H 60	153. BD*(1) O 9- S 45	2.43	2.36	-0.07
135. BD (1) C 59- H 61	153. BD*(1) O 9- S 45	0.67	0.67	0.00
136. BD (1) C 59- H 62	180. BD*(1) S 45- C 55	1.54	1.50	-0.04
136. BD (1) C 59- H 62	468. RY (4) S 45	0.59	0.91	0.32
from unit 6 to unit 7				
78. LP (1) S 45	170. BD*(1) C 30- H 31	0.38	0.00	-0.38
78. LP (1) S 45	172. BD*(1) C 30- H 33	0.09	0.00	-0.09
78. LP (1) S 45	384. RY (1) H 31	0.06	0.00	-0.06
from unit 7 to unit 1				
64. LP (1) S 15	141. BD*(1) C 1- H 4	0.07	0.07	0.00
64. LP (1) S 15	173. BD*(1) O 34- S 39	0.06	0.06	0.00
66. LP (2) O 16	173. BD*(1) O 34- S 39	0.13	0.09	-0.04
66. LP (2) O 16	398. RY (3) O 34	0.07	0.00	-0.07
67. LP (3) O 16	173. BD*(1) O 34- S 39	0.15	0.18	0.03
67. LP (3) O 16	398. RY (3) O 34	0.10	0.11	0.01
from unit 7 to unit 2				
None above threshold				
from unit 7 to unit 3				
64. LP (1) S 15	137. LV (1)Bi 6	0.19	0.13	-0.06
64. LP (1) S 15	138. LV (2)Bi 6	1.99	2.20	0.21
64. LP (1) S 15	146. BD*(1)Bi 6-Cl 14	0.22	0.21	-0.01
64. LP (1) S 15	224. RY (1)Bi 6	0.09	0.15	0.06
64. LP (1) S 15	225. RY (2)Bi 6	0.30	0.38	0.08

64. LP (1) S 15	226. RY (3)Bi 6	0.18	0.06	-0.12
65. LP (1) O 16	137. LV (1)Bi 6	8.08	7.05	-1.03
65. LP (1) O 16	138. LV (2)Bi 6	7.84	8.23	0.39
65. LP (1) O 16	146. BD*(1)Bi 6-Cl 14	0.22	0.35	0.13
65. LP (1) O 16	225. RY (2)Bi 6	1.93	2.15	0.22
65. LP (1) O 16	226. RY (3)Bi 6	0.19	0.00	-0.19
66. LP (2) O 16	137. LV (1)Bi 6	4.26	6.29	2.03
66. LP (2) O 16	138. LV (2)Bi 6	0.31	2.59	2.28
66. LP (2) O 16	146. BD*(1)Bi 6-Cl 14	2.04	2.43	0.39
66. LP (2) O 16	224. RY (1)Bi 6	0.07	0.07	0.00
66. LP (2) O 16	225. RY (2)Bi 6	0.09	0.08	-0.01
66. LP (2) O 16	227. RY (4)Bi 6	0.00	0.06	0.06
67. LP (3) O 16	137. LV (1)Bi 6	14.97	11.09	-3.88
67. LP (3) O 16	138. LV (2)Bi 6	35.06	36.86	1.80
67. LP (3) O 16	146. BD*(1)Bi 6-Cl 14	0.76	0.17	-0.59
67. LP (3) O 16	225. RY (2)Bi 6	0.19	0.22	0.03
67. LP (3) O 16	227. RY (4)Bi 6	0.92	0.87	-0.05
67. LP (3) O 16	276. RY (1)Cl 14	0.11	0.07	-0.04
100. BD (1) S 15- O 16	137. LV (1)Bi 6	0.44	0.32	-0.12
100. BD (1) S 15- O 16	138. LV (2)Bi 6	1.85	1.94	0.09
100. BD (1) S 15- O 16	146. BD*(1)Bi 6-Cl 14	0.13	0.10	-0.03
100. BD (1) S 15- O 16	225. RY (2)Bi 6	0.25	0.24	-0.01
101. BD (1) S 15- C 30	137. LV (1)Bi 6	0.06	0.05	-0.01
101. BD (1) S 15- C 30	138. LV (2)Bi 6	0.11	0.12	0.01
102. BD (1) S 15- C 51	137. LV (1)Bi 6	0.06	0.05	-0.01
102. BD (1) S 15- C 51	138. LV (2)Bi 6	0.15	0.16	0.01
from unit 7 to unit 4				
65. LP (1) O 16	147. BD*(1) S 7- O 25	0.10	0.10	0.00
65. LP (1) O 16	149. BD*(1) S 7- C 47	0.60	0.60	0.00
65. LP (1) O 16	168. BD*(1) C 26- H 28	1.07	1.08	0.01
66. LP (2) O 16	147. BD*(1) S 7- O 25	0.21	0.22	0.01
66. LP (2) O 16	149. BD*(1) S 7- C 47	1.41	1.36	-0.05
66. LP (2) O 16	229. RY (2) S 7	0.06	0.06	0.00
66. LP (2) O 16	230. RY (3) S 7	0.06	0.05	-0.01
66. LP (2) O 16	234. RY (7) S 7	0.06	0.05	-0.01
67. LP (3) O 16	168. BD*(1) C 26- H 28	0.29	0.28	-0.01
102. BD (1) S 15- C 51	234. RY (7) S 7	0.08	0.08	0.00
102. BD (1) S 15- C 51	367. RY (1) H 28	0.06	0.06	0.00
from unit 7 to unit 5				
None above threshold				
from unit 7 to unit 6				
65. LP (1) O 16	180. BD*(1) S 45- C 55	0.13	0.00	-0.13
66. LP (2) O 16	180. BD*(1) S 45- C 55	0.31	0.06	-0.25
66. LP (2) O 16	467. RY (3) S 45	0.05	0.05	0.00
within unit 7				
64. LP (1) S 15	171. BD*(1) C 30- H 32	1.89	1.89	0.00

64. LP (1) S 15	186. BD*(1) C 51- H 53	1.98	1.98	0.00
65. LP (1) O 16	159. BD*(1) S 15- C 51	0.76	0.82	0.06
65. LP (1) O 16	286. RY (2) S 15	1.90	1.98	0.08
66. LP (2) O 16	158. BD*(1) S 15- C 30	10.51	10.64	0.13
66. LP (2) O 16	159. BD*(1) S 15- C 51	2.73	1.65	-1.08
66. LP (2) O 16	172. BD*(1) C 30- H 33	0.71	0.73	0.02
66. LP (2) O 16	285. RY (1) S 15	6.05	5.79	-0.26
66. LP (2) O 16	289. RY (5) S 15	1.14	1.09	-0.05
67. LP (3) O 16	159. BD*(1) S 15- C 51	5.41	6.28	0.87
67. LP (3) O 16	185. BD*(1) C 51- H 52	0.00	0.50	0.50
67. LP (3) O 16	286. RY (2) S 15	4.89	4.63	-0.26
100. BD (1) S 15- O 16	158. BD*(1) S 15- C 30	1.03	1.03	0.00
100. BD (1) S 15- O 16	159. BD*(1) S 15- C 51	1.04	1.04	0.00
100. BD (1) S 15- O 16	172. BD*(1) C 30- H 33	0.76	0.75	-0.01
100. BD (1) S 15- O 16	185. BD*(1) C 51- H 52	0.77	0.77	0.00
100. BD (1) S 15- O 16	376. RY (2) C 30	0.64	0.62	-0.02
100. BD (1) S 15- O 16	505. RY (2) C 51	0.52	0.52	0.00
101. BD (1) S 15- C 30	157. BD*(1) S 15- O 16	0.82	0.83	0.01
101. BD (1) S 15- C 30	159. BD*(1) S 15- C 51	0.58	0.58	0.00
101. BD (1) S 15- C 30	187. BD*(1) C 51- H 54	1.30	1.30	0.00
101. BD (1) S 15- C 30	294. RY (1) O 16	0.66	0.68	0.02
102. BD (1) S 15- C 51	157. BD*(1) S 15- O 16	0.70	0.70	0.00
102. BD (1) S 15- C 51	158. BD*(1) S 15- C 30	0.61	0.60	-0.01
102. BD (1) S 15- C 51	170. BD*(1) C 30- H 31	1.25	1.26	0.01
102. BD (1) S 15- C 51	294. RY (1) O 16	0.66	0.65	-0.01
102. BD (1) S 15- C 51	375. RY (1) C 30	0.72	0.78	0.06
113. BD (1) C 30- H 31	159. BD*(1) S 15- C 51	1.55	1.56	0.01
113. BD (1) C 30- H 31	288. RY (4) S 15	1.01	1.01	0.00
114. BD (1) C 30- H 32	157. BD*(1) S 15- O 16	0.62	0.63	0.01
115. BD (1) C 30- H 33	157. BD*(1) S 15- O 16	2.48	2.48	0.00
128. BD (1) C 51- H 52	157. BD*(1) S 15- O 16	2.45	2.45	0.00
129. BD (1) C 51- H 53	157. BD*(1) S 15- O 16	0.58	0.57	-0.01
130. BD (1) C 51- H 54	158. BD*(1) S 15- C 30	1.52	1.53	0.01

Cartesian Coordinates (Ångstrom)

$[BiCl]^{2+}$			
Bi	0.000000000	0.000000000	0.391127000
Cl	0.000000000	0.000000000	-1.909619000

dmso			
S	-0.000070000	0.231208000	-0.440836000
C	-1.363219000	-0.815630000	0.184170000
H	-1.326831000	-1.788306000	-0.312528000
H	-2.298767000	-0.309044000	-0.059527000
H	-1.267222000	-0.928006000	1.267147000
C	1.363860000	-0.814625000	0.184192000
H	1.328503000	-1.787126000	-0.312958000
H	1.267727000	-0.927586000	1.267094000

H	2.299026000	-0.307114000	-0.059041000
O	-0.000645000	1.516174000	0.381627000

[BiCl(dmso)]$^{2+}$

S	-2.260602000	0.421132000	-0.262647000
Bi	1.007891000	-0.403807000	0.067705000
Cl	1.106619000	1.942658000	-0.360862000
C	-3.408383000	-0.770814000	-0.977671000
H	-4.359931000	-0.249774000	-1.108888000
H	-3.507033000	-1.621047000	-0.301783000
H	-2.999755000	-1.064658000	-1.944897000
C	-2.940898000	0.658825000	1.392141000
H	-3.891337000	1.184205000	1.264442000
H	-2.235034000	1.282788000	1.941302000
H	-3.080339000	-0.313257000	1.866548000
O	-1.016087000	-0.599200000	0.064243000

[BiCl(dmso)$_2$]$^{2+}$

S	-1.739882000	1.604493000	-0.268209000
Bi	-0.137120000	-1.187124000	0.415191000
Cl	-0.062710000	-1.241816000	-2.040591000
S	2.511553000	0.753864000	-0.307468000
O	1.108102000	0.532735000	0.465829000
C	3.481129000	1.536970000	0.996901000
H	3.690541000	0.769482000	1.742243000
H	2.902647000	2.359565000	1.420202000
H	4.409937000	1.893111000	0.544895000
C	-3.507132000	1.727500000	-0.603889000
H	-3.686802000	2.711982000	-1.042099000
H	-4.058751000	1.597225000	0.328033000
H	-3.746396000	0.944157000	-1.323634000
C	-1.524031000	2.804754000	1.062235000
H	-1.681083000	3.798169000	0.635965000
H	-0.495885000	2.696824000	1.409499000
H	-2.240145000	2.593717000	1.857498000
O	-1.699585000	0.204387000	0.545726000
C	2.136186000	2.163136000	-1.370287000
H	3.075092000	2.470629000	-1.836367000
H	1.710660000	2.967102000	-0.768542000
H	1.435457000	1.815354000	-2.129869000

[BiCl(dmso)$_3$]$^{2+}$

S	-0.558748000	2.246367000	0.353509000
Bi	0.115116000	-0.884851000	-0.605787000
S	-3.240731000	-0.742810000	0.332621000
S	3.151950000	0.157675000	0.529421000
C	3.805935000	1.382088000	-0.629675000
H	4.482369000	2.044243000	-0.085007000
H	2.942939000	1.936823000	-0.999055000
H	4.318907000	0.868195000	-1.444211000

Cl	-0.043653000	-1.335109000	1.841362000
C	4.627459000	-0.837337000	0.839724000
H	5.351331000	-0.211381000	1.366192000
H	5.025416000	-1.190012000	-0.113098000
H	4.320800000	-1.672229000	1.470550000
O	-2.015896000	-0.064563000	-0.415851000
C	-4.642383000	-0.215667000	-0.679312000
H	-5.557593000	-0.538672000	-0.178145000
H	-4.544210000	-0.714227000	-1.644112000
H	-4.614814000	0.869234000	-0.792645000
C	0.609148000	3.549173000	0.797984000
H	0.030078000	4.375585000	1.216485000
H	1.162409000	3.863086000	-0.088190000
H	1.274484000	3.134951000	1.556362000
O	2.311284000	-0.807398000	-0.415677000
C	-1.434085000	3.017024000	-1.025732000
H	-2.035563000	3.834676000	-0.622127000
H	-2.074354000	2.241472000	-1.444975000
H	-0.707220000	3.377143000	-1.755204000
O	0.440802000	1.223570000	-0.388093000
C	-3.487215000	0.290117000	1.797946000
H	-2.621728000	0.130848000	2.441930000
H	-4.397670000	-0.051687000	2.295037000
H	-3.570360000	1.336468000	1.499919000

$[BiCl(dmso)_4]^{2+}$ (**A**)

S	-0.082247000	2.598564000	0.714238000
Bi	-0.038087000	-0.293691000	-1.130156000
S	-3.155607000	-0.116402000	0.266213000
S	3.143178000	0.047846000	0.183441000
C	3.840847000	1.276146000	-0.948329000
H	4.654742000	1.789344000	-0.430789000
H	3.033258000	1.972127000	-1.177156000
H	4.198043000	0.769516000	-1.846704000
Cl	-0.777035000	-2.845041000	-1.087244000
S	0.435384000	-1.710785000	1.887642000
O	-0.242300000	-0.539634000	1.041264000
C	4.534094000	-1.099200000	0.315326000
H	5.362462000	-0.577275000	0.798933000
H	4.806761000	-1.437785000	-0.685678000
H	4.201025000	-1.934120000	0.932708000
O	-2.069556000	0.594264000	-0.644797000
C	-4.692790000	0.294286000	-0.591289000
H	-5.526985000	-0.016259000	0.041466000
H	-4.698583000	-0.267728000	-1.525922000
H	-4.716499000	1.368896000	-0.780107000
C	-0.977581000	-2.584256000	2.597448000
H	-1.500761000	-3.046652000	1.760068000
H	-1.616826000	-1.880682000	3.132469000
H	-0.583723000	-3.347863000	3.272207000

C	1.283776000	3.547709000	1.420355000
H	0.867164000	4.268561000	2.127016000
H	1.827402000	4.047973000	0.617192000
H	1.924860000	2.836071000	1.942808000
O	2.133423000	-0.787263000	-0.703488000
C	-0.927803000	3.880408000	-0.240837000
H	-1.350104000	4.609140000	0.454765000
H	-1.723003000	3.370274000	-0.786671000
H	-0.215456000	4.345792000	-0.924141000
O	0.650331000	1.747510000	-0.403357000
C	-3.293799000	0.970076000	1.705734000
H	-2.328204000	0.935369000	2.210641000
H	-4.077915000	0.570338000	2.352818000
H	-3.531002000	1.984381000	1.380780000
C	1.019923000	-0.785865000	3.326210000
H	1.393403000	-1.503770000	4.059675000
H	0.195388000	-0.195611000	3.728858000
H	1.828824000	-0.140338000	2.981638000

$[BiCl(dmso)_4]^{2+}$ (**B**)

C	3.610812000	1.122709000	-0.971684000
H	4.425800000	0.983969000	-1.685487000
H	3.846370000	1.901662000	-0.244448000
H	2.679639000	1.340056000	-1.494222000
Bi	0.107026000	-0.235951000	1.075827000
S	-0.213641000	-0.854154000	-2.204624000
S	-0.708256000	2.745168000	-0.399070000
C	0.420334000	4.118400000	-0.731775000
H	-0.065008000	4.795558000	-1.438137000
H	1.319572000	3.691892000	-1.177393000
H	0.653523000	4.623608000	0.207086000
Cl	0.011504000	-2.836436000	0.476318000
S	-3.189489000	-1.052372000	0.489856000
O	-1.986494000	-0.085572000	0.134115000
C	-2.054609000	3.628441000	0.423477000
H	-2.523822000	4.293896000	-0.304314000
H	-1.653730000	4.186144000	1.271421000
H	-2.764548000	2.870814000	0.755697000
C	4.910365000	-0.557007000	0.806351000
H	4.842342000	-1.433205000	1.451956000
H	5.046478000	0.351784000	1.395178000
H	5.716187000	-0.684191000	0.080132000
O	0.560821000	-0.056353000	-1.055760000
C	1.118217000	-1.778601000	-2.999296000
H	0.688860000	-2.302185000	-3.856329000
H	1.474219000	-2.494149000	-2.257356000
H	1.910706000	-1.097008000	-3.310831000
C	-3.419491000	-2.048304000	-1.003018000
H	-2.548602000	-2.700195000	-1.077364000
H	-3.495238000	-1.389314000	-1.869764000

H	-4.328885000	-2.639277000	-0.873019000
O	2.342811000	-0.065256000	1.066349000
S	3.347878000	-0.440712000	-0.097755000
O	0.008679000	1.993506000	0.791578000
C	-0.497816000	0.456158000	-3.413254000
H	-1.254693000	1.116810000	-2.990486000
H	-0.868535000	-0.011352000	-4.327853000
H	0.435590000	0.992384000	-3.590575000
C	-4.614617000	0.052883000	0.358066000
H	-5.523209000	-0.551124000	0.407787000
H	-4.549371000	0.594708000	-0.587276000
H	-4.571955000	0.736527000	1.206544000

$[BiCl(dmso)_4]^{2+}$ (**C**)

S	1.731803000	2.399049000	0.555082000
Bi	0.090809000	-0.321880000	-0.656553000
S	-2.348427000	1.872866000	0.342905000
S	3.092942000	-1.660680000	0.438356000
C	4.373723000	-1.050280000	-0.687480000
H	5.323683000	-1.016156000	-0.149477000
H	4.061356000	-0.046757000	-0.978230000
H	4.432120000	-1.708755000	-1.556120000
Cl	-0.033888000	-0.529045000	1.863683000
S	-2.770768000	-2.205402000	0.401973000
O	-2.166326000	-1.061965000	-0.494809000
C	3.641706000	-3.372765000	0.643284000
H	4.606856000	-3.363933000	1.154675000
H	3.718066000	-3.841754000	-0.339253000
H	2.894871000	-3.876841000	1.257886000
O	-0.980937000	1.667715000	-0.434067000
C	-3.584962000	1.872817000	-0.977659000
H	-4.550788000	2.142363000	-0.544052000
H	-3.609014000	0.852554000	-1.361749000
H	-3.282978000	2.582456000	-1.749695000
C	-4.126879000	-2.843512000	-0.613087000
H	-3.676852000	-3.326561000	-1.481287000
H	-4.766874000	-2.013557000	-0.917848000
H	-4.681388000	-3.574395000	-0.020431000
C	3.463565000	2.592235000	1.036051000
H	3.563860000	3.543236000	1.564061000
H	4.090926000	2.567522000	0.143566000
H	3.700588000	1.764076000	1.704965000
O	1.829512000	-1.814356000	-0.492380000
C	1.574627000	3.717774000	-0.672474000
H	1.627133000	4.677789000	-0.154483000
H	0.593204000	3.582922000	-1.127930000
H	2.371320000	3.618553000	-1.411467000
O	1.799078000	1.085322000	-0.337407000
C	-2.285741000	3.636790000	0.732228000
H	-1.485827000	3.774746000	1.460684000

H	-3.245123000	3.921446000	1.169185000
H	-2.085375000	4.198725000	-0.181552000
C	-3.725442000	-1.339196000	1.675764000
H	-4.250295000	-2.087400000	2.274141000
H	-4.429033000	-0.652037000	1.202393000
H	-3.004493000	-0.798661000	2.289815000

$[BiCl(dmso)_5]^{2+}$ (**A**)

C	2.667444000	-3.876035000	0.834709000
H	3.626821000	-4.096801000	1.307986000
H	2.527917000	-4.476610000	-0.065895000
H	1.853935000	-4.042756000	1.542190000
S	-0.886338000	3.258450000	0.419093000
Bi	0.000748000	0.000701000	-0.569607000
S	-3.380050000	0.158011000	0.399138000
S	2.828546000	1.848685000	0.415046000
C	3.216171000	3.072403000	-0.862847000
H	3.716046000	3.923515000	-0.395203000
H	2.256483000	3.372422000	-1.283305000
H	3.849653000	2.607606000	-1.620719000
Cl	0.000034000	0.003105000	1.960256000
S	-1.199169000	-3.152565000	0.424549000
O	-1.483321000	-1.842359000	-0.395866000
C	4.506955000	1.323540000	0.849394000
H	5.012813000	2.160085000	1.336750000
H	5.038874000	1.016517000	-0.052832000
H	4.409127000	0.488875000	1.544948000
C	3.910576000	-2.102005000	-0.887082000
H	3.895415000	-1.094900000	-1.303465000
H	3.657216000	-2.844919000	-1.645754000
H	4.878611000	-2.316392000	-0.429060000
O	-2.211541000	0.843110000	-0.397879000
C	-4.396635000	-0.599903000	-0.894353000
H	-5.307146000	-0.995660000	-0.439111000
H	-3.791953000	-1.405218000	-1.311079000
H	-4.626704000	0.151363000	-1.652254000
C	-0.807416000	-4.379233000	-0.848678000
H	0.142799000	-4.064056000	-1.279469000
H	-1.599330000	-4.377461000	-1.600148000
H	-0.711776000	-5.358942000	-0.375282000
O	1.293544000	-1.982591000	-0.398897000
C	0.128139000	4.694954000	0.853709000
H	-0.515904000	5.437828000	1.329505000
H	0.593083000	5.099572000	-0.047031000
H	0.885385000	4.349787000	1.558961000
S	2.638831000	-2.117234000	0.402306000
O	2.284921000	0.618894000	-0.398883000
C	-1.932128000	4.004038000	-0.858106000
H	-2.591858000	4.736853000	-0.388165000
H	-2.508380000	3.182537000	-1.283552000

H	-1.295900000	4.469729000	-1.613126000
O	0.118190000	2.364614000	-0.394738000
C	-4.439878000	1.562403000	0.830547000
H	-3.882734000	2.176915000	1.539072000
H	-5.345927000	1.175051000	1.302092000
H	-4.679528000	2.130096000	-0.070336000
C	-2.863803000	-3.706121000	0.875629000
H	-2.779089000	-4.674186000	1.374584000
H	-3.480876000	-3.780933000	-0.021437000
H	-3.269746000	-2.964050000	1.564604000

$[BiCl(dmso)_5]^{2+}$ (**B**)

S	-1.802319000	2.270578000	1.092304000
Bi	-0.165937000	-0.046751000	-0.745328000
S	2.113281000	2.225834000	0.161516000
S	-3.541288000	-1.137989000	-0.931213000
O	-0.379057000	-1.148650000	1.240529000
C	-3.580750000	-1.849819000	0.737721000
H	-4.615465000	-1.846805000	1.087467000
H	-2.954390000	-1.208497000	1.356676000
H	-3.177077000	-2.863644000	0.704973000
Cl	-0.289066000	2.180408000	-2.328055000
S	3.189772000	-1.677832000	-1.225279000
O	2.284988000	-0.781148000	-0.322385000
C	-4.353422000	-2.481223000	-1.836472000
H	-5.384804000	-2.558205000	-1.484808000
H	-3.806037000	-3.409555000	-1.663038000
H	-4.336665000	-2.213966000	-2.893866000
O	0.881373000	1.374615000	0.681219000
C	3.347495000	1.965183000	1.454476000
H	4.161373000	2.676701000	1.298707000
H	3.708973000	0.944889000	1.328694000
H	2.880037000	2.104026000	2.430815000
C	4.155861000	-2.668163000	-0.051370000
H	3.461844000	-3.354824000	0.435265000
H	4.617396000	-2.001973000	0.680742000
H	4.912085000	-3.229036000	-0.605180000
C	-3.570247000	2.619622000	1.230631000
H	-3.696102000	3.483641000	1.886580000
H	-4.076471000	1.739095000	1.629593000
H	-3.926853000	2.852178000	0.226650000
O	-2.046324000	-1.276217000	-1.397417000
C	-1.433100000	1.761151000	2.789751000
H	-1.521584000	2.638368000	3.434289000
H	-0.407274000	1.395887000	2.770591000
H	-2.126295000	0.973606000	3.088337000
S	0.607735000	-2.266021000	1.757958000
O	-1.856298000	0.899722000	0.294451000
C	1.599760000	3.920177000	0.526471000
H	0.774209000	4.137163000	-0.152344000

H	2.443052000	4.585351000	0.329067000
H	1.280297000	3.983167000	1.568151000
C	4.503494000	-0.568485000	-1.804314000
H	5.229920000	-1.154185000	-2.371909000
H	4.973080000	-0.086001000	-0.944699000
H	4.031278000	0.173109000	-2.450232000
C	-0.410373000	-3.106007000	2.994457000
H	-0.843563000	-2.358208000	3.661131000
H	0.225944000	-3.804914000	3.541780000
H	-1.190586000	-3.647585000	2.458742000
C	1.743944000	-1.374651000	2.846626000
H	2.385483000	-2.101421000	3.349544000
H	1.168177000	-0.787615000	3.564080000
H	2.328584000	-0.733783000	2.189802000

$[BiCl(dmso)_6]^{2+}$ (**A**)

C	3.237605000	-2.969547000	-1.884950000
H	3.321624000	-3.979038000	-2.293776000
H	4.006554000	-2.786808000	-1.132124000
H	3.299844000	-2.234260000	-2.688309000
S	-3.611328000	1.049496000	-0.004812000
Bi	-0.182132000	-0.003784000	0.031340000
S	-0.349485000	3.489231000	-0.624838000
S	-2.319666000	-2.804035000	-0.318560000
O	0.821090000	0.002414000	2.214323000
C	-3.258968000	-3.068782000	1.208180000
H	-4.213025000	-3.538452000	0.957930000
H	-3.415298000	-2.076504000	1.630915000
H	-2.673152000	-3.697306000	1.881837000
Cl	-0.692246000	-0.011020000	-2.577855000
S	2.712114000	1.080834000	-1.592778000
O	1.674318000	1.387252000	-0.452464000
C	-2.004176000	-4.531485000	-0.770590000
H	-2.955203000	-4.998120000	-1.037495000
H	-1.536073000	-5.048350000	0.069421000
H	-1.338162000	-4.518504000	-1.634569000
C	1.790418000	-3.957368000	0.249456000
H	0.888149000	-3.848688000	0.851025000
H	2.681580000	-3.686375000	0.819564000
H	1.869418000	-4.970857000	-0.150106000
O	-0.942624000	2.293997000	0.193724000
C	0.802658000	4.290955000	0.522982000
H	1.154084000	5.224456000	0.077520000
H	1.626949000	3.589605000	0.650478000
H	0.290791000	4.471296000	1.470411000
C	4.233204000	0.686173000	-0.694116000
H	4.038753000	-0.254970000	-0.181065000
H	4.441977000	1.482547000	0.022968000
H	5.047005000	0.575731000	-1.414108000
O	1.690061000	-1.386502000	-0.443540000

C	-5.192413000	0.160140000	-0.028151000
H	-5.986002000	0.866099000	-0.283145000
H	-5.368809000	-0.290404000	0.950495000
H	-5.114776000	-0.609440000	-0.797647000
S	1.600339000	-2.796756000	-1.128743000
O	-0.917819000	-2.308788000	0.176991000
C	-3.913465000	2.136618000	1.414489000
H	-4.694736000	2.853601000	1.151820000
H	-2.967999000	2.646958000	1.597926000
H	-4.205527000	1.529901000	2.274212000
S	2.366863000	-0.080029000	2.441343000
O	-2.605612000	-0.019806000	0.532525000
C	-1.687940000	4.712022000	-0.593576000
H	-2.510888000	4.300035000	-1.179300000
H	-1.324217000	5.633484000	-1.053923000
H	-1.998099000	4.885688000	0.438596000
C	3.137006000	2.735974000	-2.194563000
H	3.935725000	2.639864000	-2.933372000
H	3.450903000	3.360368000	-1.356034000
H	2.239688000	3.142103000	-2.663465000
C	2.551946000	-1.383178000	3.687269000
H	1.889179000	-1.172993000	4.529029000
H	3.596662000	-1.416561000	4.004884000
H	2.276078000	-2.322785000	3.206672000
C	2.734148000	1.363915000	3.473793000
H	3.771787000	1.299599000	3.809119000
H	2.045776000	1.384272000	4.320932000
H	2.597755000	2.245195000	2.845412000

$[BiCl(dmso)_6]^{2+}$ (**B**)

C	-2.066759000	4.135679000	0.280792000
H	-2.158846000	5.047197000	0.875442000
H	-2.972733000	3.949802000	-0.299292000
H	-1.196820000	4.196492000	-0.373579000
S	3.329262000	-0.364059000	1.490021000
Bi	-0.068142000	-0.174804000	0.568173000
S	1.563829000	-1.312638000	-2.344286000
S	2.033495000	2.156999000	-1.245300000
O	-0.891688000	-2.344035000	0.323776000
C	3.389611000	3.016969000	-0.404811000
H	4.070930000	3.411893000	-1.161669000
H	3.901789000	2.280277000	0.214463000
H	2.971766000	3.817924000	0.208302000
Cl	-1.353811000	-0.438217000	3.101421000
S	-1.650740000	0.895530000	-2.385000000
O	-1.128976000	-0.331193000	-1.547732000
C	1.328477000	3.557838000	-2.155752000
H	2.037778000	3.866501000	-2.926792000
H	1.127009000	4.373431000	-1.458350000
H	0.403085000	3.204129000	-2.612825000

C	-3.358326000	2.694736000	2.269109000
H	-3.320383000	1.810901000	2.907815000
H	-4.168107000	2.616316000	1.540681000
H	-3.452276000	3.601980000	2.870495000
O	1.602495000	-1.134908000	-0.782384000
C	0.715329000	-2.891644000	-2.593509000
H	0.737110000	-3.135276000	-3.657893000
H	-0.304442000	-2.728275000	-2.249865000
H	1.205619000	-3.660931000	-1.994519000
C	-3.446312000	0.856449000	-2.157239000
H	-3.614652000	1.108977000	-1.110941000
H	-3.810403000	-0.146870000	-2.387426000
H	-3.895918000	1.602782000	-2.816298000
O	-1.834561000	1.494036000	0.445243000
C	4.212317000	0.076511000	3.009426000
H	5.285599000	0.052465000	2.807227000
H	3.942742000	-0.630781000	3.796044000
H	3.903418000	1.087718000	3.278022000
S	-1.774084000	2.735758000	1.392946000
O	0.985161000	1.879255000	-0.120102000
C	3.903079000	-2.071583000	1.298123000
H	4.968955000	-2.060272000	1.059559000
H	3.325790000	-2.484128000	0.471231000
H	3.709688000	-2.619939000	2.222234000
S	-2.452802000	-2.514166000	0.428832000
O	1.845028000	-0.495536000	1.968420000
C	3.265728000	-1.801464000	-2.717679000
H	3.899400000	-0.945828000	-2.480739000
H	3.328717000	-2.034117000	-3.782967000
H	3.538590000	-2.665991000	-2.110288000
C	-1.548054000	0.261795000	-4.078144000
H	-2.027051000	0.977799000	-4.749354000
H	-2.039983000	-0.711864000	-4.120737000
H	-0.488564000	0.168473000	-4.319705000
C	-2.655065000	-3.749585000	1.734279000
H	-2.011259000	-4.604183000	1.517712000
H	-3.705921000	-4.044545000	1.777955000
H	-2.356037000	-3.254848000	2.659527000
C	-2.863228000	-3.508520000	-1.026716000
H	-3.903900000	-3.830339000	-0.947140000
H	-2.187798000	-4.364800000	-1.077290000
H	-2.736178000	-2.856466000	-1.892027000

$[BiCl(dmso)_7]^{2+}$

C	-4.828997000	1.136087000	-1.242892000
H	-5.472250000	2.014609000	-1.330514000
H	-5.210100000	0.439402000	-0.493553000
H	-4.731341000	0.642728000	-2.210872000
S	3.401429000	0.713436000	-1.000392000
Bi	-0.095618000	0.015183000	-0.275123000

S	2.161833000	-3.088408000	-0.902519000
S	0.199372000	3.475997000	-0.799658000
O	-1.027511000	0.182105000	1.997217000
C	1.388173000	4.282773000	0.303600000
H	1.730259000	5.205196000	-0.171299000
H	2.212556000	3.582790000	0.421204000
H	0.903407000	4.487319000	1.260243000
Cl	-0.383592000	0.411257000	-2.923300000
S	-2.175554000	-2.467669000	-1.550557000
O	-1.247789000	-2.108499000	-0.340889000
C	-1.086746000	4.755331000	-0.792161000
H	-0.680245000	5.653691000	-1.261936000
H	-1.392851000	4.955612000	0.236443000
H	-1.923564000	4.373779000	-1.377527000
C	-3.552314000	2.352722000	0.888253000
H	-2.584998000	2.532835000	1.357135000
H	-4.130841000	1.620784000	1.455085000
H	-4.107553000	3.285217000	0.765918000
O	1.514118000	-1.737936000	-1.310089000
C	1.285444000	-3.730680000	0.559954000
H	1.781404000	-4.658333000	0.854818000
H	0.254753000	-3.915302000	0.263715000
H	1.322901000	-2.976238000	1.347007000
C	-3.272055000	-3.735006000	-0.862547000
H	-3.914011000	-3.235206000	-0.135632000
H	-2.673370000	-4.509417000	-0.379191000
H	-3.875336000	-4.151332000	-1.672563000
O	-2.452667000	0.322699000	-0.459017000
C	3.316360000	0.500874000	-2.801200000
H	4.300816000	0.188680000	-3.158175000
H	3.007423000	1.441404000	-3.261191000
H	2.572785000	-0.277110000	-2.972765000
S	-3.170369000	1.674862000	-0.751929000
O	-0.442704000	2.342739000	0.081237000
C	4.517633000	2.144948000	-0.953040000
H	5.479283000	1.847932000	-1.377924000
H	4.646473000	2.428528000	0.092703000
H	4.074603000	2.964052000	-1.523410000
S	-2.225423000	-0.642072000	2.560346000
O	2.026290000	1.312247000	-0.583505000
C	3.728226000	-2.686897000	-0.067621000
H	4.408260000	-2.281334000	-0.817175000
H	4.136406000	-3.611066000	0.348546000
H	3.515361000	-1.950130000	0.707690000
C	-1.125915000	-3.498439000	-2.613314000
H	-1.697678000	-3.784046000	-3.498976000
H	-0.785612000	-4.379815000	-2.066243000
H	-0.281271000	-2.863258000	-2.885631000
C	-2.348251000	-0.044358000	4.269210000
H	-1.364026000	-0.112915000	4.736890000

H	-3.083436000	-0.651104000	4.802386000
H	-2.679548000	0.994032000	4.223263000
C	-1.558396000	-2.299180000	2.894587000
H	-2.315903000	-2.876305000	3.429789000
H	-0.643834000	-2.206088000	3.483475000
H	-1.354945000	-2.745037000	1.920653000
O	1.661123000	-0.769818000	1.565971000
S	1.727715000	-0.225827000	3.017846000
C	3.488544000	-0.373355000	3.448805000
C	1.661162000	1.587216000	2.883597000
H	4.084241000	0.118058000	2.676054000
H	3.722342000	-1.438386000	3.488901000
H	3.656974000	0.084370000	4.426384000
H	2.376740000	1.897382000	2.121286000
H	1.897774000	2.020793000	3.857634000
H	0.647218000	1.846022000	2.579718000

References

(1) Carmalt, C. J.; Farrugia, L. J.; Norman, N. C. Structural Studies on some Iodoantimonate and Iodobismuthate Anions. *Z. anorg. allg.* **1995**, *621* (1), 47-56. DOI: 10.1002/zaac.19956210110.

(2) Barbour, L. J.; Belfield, S. J.; Junk, P. C.; Smith, M. K. Bidentate Nitrogen Base Adducts of Bismuth(III) Nitrate. *Aust. J. Chem.* **1998**, *51* (4), 337-342. DOI: 10.1071/C97170.

(3) Frank, W.; Reiss, G. J.; Schneider, J. The nonaaquabismuth (III) cation. *Angew. Chem., Int. Ed.* **1995**, *34* (21), 2416-2417. DOI: 10.1002/anie.199524161.

(4) Näslund, J.; Persson, I.; Sandström, M. Solvation of the Bismuth(III) Ion by Water, Dimethyl Sulfoxide, N,N‘-Dimethylpropyleneurea, and N,N-Dimethylthioformamide. An EXAFS, Large-Angle X-ray Scattering, and Crystallographic Structural Study. *Inorg. Chem.* **2000**, *39* (18), 4012-4021. DOI: 10.1021/ic000022m. Shannon, R. Revised effective ionic radii and systematic studies of interatomic distances in halides and chalcogenides. *Acta Crystallogr., Sect. A* **1976**, *32* (5), 751-767. DOI: 10.1107/S0567739476001551.

(5) Balasubramaniam, S.; Kumar, S.; Andrews, A. P.; Varghese, B.; Jemmis, E. D.; Venugopal, A. A dicationic bismuth (III) lewis acid: catalytic hydrosilylation of olefins. *Eur. J. Inorg. Chem.* **2019**, (28), 3265-3269. DOI: 10.1002/ejic.201900459.

(6) Möbs, J.; Gerhard, M.; Heine, J. $(HPy)_2(Py)CuBi_3I_{12}$, a low bandgap metal halide photoconductor. *Dalton Trans.* **2020**, *49* (41), 14397-14400. DOI: 10.1039/d0dt03427d.

(7) Szklarz, P.; Jakubas, R.; Medycki, W.; Gągor, A.; Cichos, J.; Karbowiak, M.; Bator, G. $(C_3N_2H_5)_3Sb_2I_9$ and $(C_3N_2H_5)_3Bi_2I_9$: ferroelastic lead-free hybrid perovskite-like materials as potential semiconducting absorbers. *Dalton Trans.* **2022**, *51* (5), 1850-1860. DOI: 10.1039/d1dt03455c.

(8) Eckhardt, K.; Bon, V.; Getzschmann, J.; Grothe, J.; Wisser, F. M.; Kaskel, S. Crystallographic insights into (CH 3 NH 3) 3 (Bi 2 I 9): a new lead-free hybrid organic–inorganic material as a potential absorber for photovoltaics. *Chem. Commun.* **2016**, *52* (14), 3058-3060. DOI: 10.1039/c5cc10455f.

(9) (a) Szklarz, P.; Gągor, A.; Jakubas, R.; Zieliński, P.; Piecha-Bisiorek, A.; Cichos, J.; Karbowiak, M.; Bator, G.; Ciżman, A. Lead-free hybrid ferroelectric material based on formamidine:$[NH_2CHNH_2]_3Bi_2I_9$. *J. Mater. Chem. C* **2019**, *7* (10), 3003-3014. DOI: 10.1039/c8tc06458j. (b) Szklarz, P.; Jakubas, R.; Gągor, A.; Bator, G.; Cichos, J.; Karbowiak, M. $[NH_2CHNH_2]_3Sb_2I_9$: a lead-free and low-toxicity organic–inorganic hybrid ferroelectric based on antimony (iii) as a potential semiconducting absorber. *Inorg. Chem. Front.* **2020**, *7* (8), 1780-1789. DOI: 10.1039/d0qi00137f.

(10) Möbs, J.; Pan, S.; Tonner-Zech, R.; Heine, J. $[SMe_3]_2[Bi_2Ag_2I_{10}]$, a silver iodido bismuthate with an unusually small band gap. *Dalton Trans.* **2022**, *51* (36), 13771-13778. DOI: 10.1039/d2dt02305a.

(11) Möbs, J.; Stuhrmann, G.; Wippermann, S.; Heine, J. Optical Properties and Metal-Dependent Charge Transfer in Iodido Pentelates. *ChemPlusChem* **2023**, *88* (6), e202200403. DOI: 10.1002/cplu.202200403.
(12) Hao, P.; Wang, W.; Shen, J.; Fu, Y. Non-transient thermo-/photochromism of iodobismuthate hybrids directed by solvated metal cations. *Dalton Trans.* **2020**, *49* (6), 1847-1853. DOI: 10.1039/c9dt04818a.
(13) Jiang, Y.-X.; Wang, Y.-Y.; Song, L.; Wang, D.-D.; Guo, J.-Y.; Shen, H.-Y.; Wang, X.-R.; Chai, W.-X. The Optical Band-Gap Evolution in Perovskite-Like Hybrid Iodobismuthates Effected by Nuclearity and Dimension: An Experimental and DFT Calculation Study. *J. Cluster Sci.* **2019**, *30*, 1443-1454. DOI: 10.1007/s10876-019-01587-8.
(14) Kotov, V. Y.; Ilyukhin, A. B.; Baranchikov, A. E.; Ishmetova, R. I.; Rusinov, G. L.; Kozyukhin, S. A. Synthesis, crystal structure and optical properties of 1,1'-(1,n-alkanediyl)bis(3-methylimidazolium) halobismuthates. *J. Mol. Struct.* **2018**, *1151*, 186-190. DOI: 10.1016/j.molstruc.2017.09.040.
(15) Zhang, J.; Han, S.; Ji, C.; Zhang, W.; Wang, Y.; Tao, K.; Sun, Z.; Luo, J. $[(CH_3)_3NH]_3Bi_2I_9$: A Polar Lead-Free Hybrid Perovskite-Like Material as a Potential Semiconducting Absorber. *Chem. - Eur. J.* **2017**, *23* (68), 17304-17310. DOI: 10.1002/chem.201703346.
(16) Ghasemi, M.; Lyu, M.; Roknuzzaman, M.; Yun, J.-H.; Hao, M.; He, D.; Bai, Y.; Chen, P.; Bernhardt, P. V.; Ostrikov, K. K. Phenethylammonium bismuth halides: from single crystals to bulky-organic cation promoted thin-film deposition for potential optoelectronic applications. *J. Mater. Chem. A* **2019**, *7* (36), 20733-20741. DOI: 10.1039/c9ta07454f.
(17) Li, T.; Wang, Q.; Nichol, G. S.; Morrison, C. A.; Han, H.; Hu, Y.; Robertson, N. Extending lead-free hybrid photovoltaic materials to new structures: thiazolium, aminothiazolium and imidazolium iodobismuthates. *Dalton Trans.* **2018**, *47* (20), 7050-7058. DOI: 10.1039/c8dt00864g.
(18) Katti, K. V. Synthesis and Reactivity in Inorganic, Metal-Organic and Nano-Metal Chemistry. *Synth. React. Inorg., Met.-Org., Nano-Met. Chem.* **2005**, *35* (1), 1-2. DOI: 10.1081/SIM-200047489.
(19) Mishra, S.; Jeanneau, E.; Iasco, O.; Ledoux, G.; Luneau, D.; Daniele, S. Heterometallic, Hybrid, Heavy Main-Group Iodometallates Containing Lanthanide Complexes: Template Synthesis, Structures, Thermal, Optical, Luminescent and Magnetic Properties. *Eur. J. Inorg. Chem.* **2012**, (16), 2749-2758. DOI: 10.1002/ejic.201101363.
(20) Usoltsev, A. N.; Elshobaki, M.; Adonin, S. A.; Frolova, L. A.; Derzhavskaya, T.; Abramov, P. A.; Anokhin, D. V.; Korolkov, I. V.; Luchkin, S. Y.; Dremova, N. N. Polymeric iodobismuthates $\{[Bi_3I_{10}]\}$ and $\{[BiI_4]\}$ with N-heterocyclic cations: promising perovskite-like photoactive materials for electronic devices. *J. Mater. Chem. A* **2019**, *7* (11), 5957-5966. DOI: 10.1039/c8ta09204d.
(21) Wang, R.-Y.; Zhang, X.; Huo, Q.-S.; Yu, J.-H.; Xu, J.-Q. New discrete iodometallates with in situ generated triimidazole derivatives as countercations ($M^{n+}= Ag^+, Pb^{2+}, Bi^{3+}$). *RSC Adv.* **2017**, *7* (31), 19073-19080. DOI: 10.1039/c6ra27510a.

(22) Dammak, H.; Yangui, A.; Triki, S.; Abid, Y.; Feki, H. Structural characterization, vibrational, optical properties and DFT investigation of a new luminescent organic–inorganic material:$(C_6H_{14}N)_3Bi_2I_9$. *J. Lumin.* **2015**, *161*, 214-220. DOI: 10.1016/j.jlumin.2015.01.010.
(23) Zhang, W.; Liu, X.; Li, L.; Sun, Z.; Han, S.; Wu, Z.; Luo, J. Triiodide-induced band-edge reconstruction of a lead-free perovskite-derivative hybrid for strong light absorption. *Chem. Mater.* **2018**, *30* (12), 4081-4088. DOI: 10.1021/acs.chemmater.8b01200.
(24) Wang, M.; Huang, S.-W.; Li, J.-B.; Gong, A.-W.; Wu, H.-Y.; Li, H.-H.; Chen, Z.-R. Lanthanide–Bismuth Heterometallics Combined Lanthanide Metal Complexes with Bismuth Iodides: Structures and Properties. *J. Cluster Sci.* **2012**, *23*, 383-393. DOI: 0.1007/s10876-012-0440-8.
(25) Kou, B.; Zhang, W.; Ji, C.; Wu, Z.; Zhang, S.; Liu, X.; Luo, J. Tunable optical absorption in lead-free perovskite-like hybrids by iodide management. *Chem. Commun.* **2019**, *55* (94), 14174-14177. DOI: 10.1039/c9cc05365d.
(26) McCall, K. M.; Stoumpos, C. C.; Kostina, S. S.; Kanatzidis, M. G.; Wessels, B. W. Strong Electron–Phonon Coupling and Self-Trapped Excitons in the Defect Halide Perovskites $A_3M_2I_9$ (A = Cs, Rb; M = Bi, Sb). *Chem. Mater.* **2017**, *29* (9), 4129-4145. DOI: 10.1021/acs.chemmater.7b01184.
(27) Wang, D.-H.; Zhao, L.-M.; Lin, X.-Y.; Wang, Y.-K.; Zhang, W.-T.; Song, K.-Y.; Li, H.-H.; Chen, Z.-R. Iodoargentate/iodobismuthate-based materials hybridized with lanthanide-containing metalloviologens: thermochromic behaviors and photocurrent responses. *Inorg. Chem. Front.* **2018**, *5* (5), 1162-1173. DOI: 10.1039/c7qi00755h.
(28) Fisher, G. A.; Norman, N. C. The structures of the group 15 element (III) halides and halogenoanions. In *Advances in Inorganic Chemistry*, Vol. 41; Elsevier, 1994; pp 233-271.
(29) Brown, I. Bond valence as an aid to understanding the stereochemistry of O and F complexes of Sn (II), Sb (III), Te (IV), I (V) and Xe (VI). *J. Solid State Chem.* **1974**, *11* (3), 214-233. DOI: 10.1016/S0022-4596(74)80006-X.
(30) Bowmaker, G. A.; Harrowfield, J. M.; Junk, P. C.; Skelton, B. W.; White, A. H. Syntheses, Structures and Vibrational Spectra of Some Dimethyl Sulfoxide Solvates of Bismuth(III) Bromide and Iodide. *Aust. J. Chem.* **1998**, *51* (4), 285-292. DOI: 10.1071/C97035.
(31) Nørby, P.; Jørgensen, M. R. V.; Johnsen, S.; Brummerstedt Iversen, B. Bismuth Iodide Hybrid Organic-Inorganic Crystal Structures and Utilization in Formation of Textured BiI_3 Film. *Eur. J. Inorg. Chem.* **2016**, (9), 1389-1394. DOI: 10.1002/ejic.201501418.
(32) Yue, C.-Y.; Zhao, H.-F.; Jiang, H.; Guo, Y.-H.; Che, H.-X.; Li, J.-Z.; Chu, W.-X.; Yuan, Y.; Jing, Z.-H.; Lei, X.-W. Large conjugated organic cations sensitized hybrid lead halides as visible light driven photocatalysts. *Cryst. Growth Des.* **2019**, *19* (8), 4564-4570. DOI: 10.1021/acs.cgd.9b00395.
(33) (a) Goforth, A. M.; Tershansy, M. A.; Smith, M. D.; Peterson Jr, L.; Kelley, J. G.; DeBenedetti, W. J.; zur Loye, H.-C. Structural diversity and thermochromic properties of iodobismuthate materials

containing d-metal coordination cations: observation of a high symmetry $[Bi_3I_{11}]^{2-}$ anion and of isolated I^- anions. *J. Am. Chem. Soc.* **2011**, *133* (3), 603-612. DOI: 10.1021/ja108278j. (b) Louvain, N.; Mercier, N.; Boucher, F. α-to β-(dmes) BiI_5 (dmes= Dimethyl (2-ethylammonium) sulfonium Dication): Umbrella Reversal of Sulfonium in the Solid State and Short I··· I Interchain Contacts - Crystal Structures, Optical Properties, and Theoretical Investigations of 1D Iodobismuthates. *Inorg. Chem.* **2009**, *48* (3), 879-888. DOI: 10.1021/ic801900r. (c) Mitzi, D. B.; Brock, P. Structure and optical properties of several organic− inorganic hybrids containing corner-sharing chains of bismuth iodide Octahedra. *Inorg. Chem.* **2001**, *40* (9), 2096-2104. DOI: 10.1021/ic000622l. (d) Zhu, X.-H.; Mercier, N.; Frère, P.; Blanchard, P.; Roncali, J.; Allain, M.; Pasquier, C.; Riou, A. Effect of mono-versus di-ammonium cation of 2, 2 ‘-bithiophene derivatives on the structure of organic− inorganic hybrid materials based on iodo metallates. *Inorg. Chem.* **2003**, *42* (17), 5330-5339. DOI: 10.1021/ic034235y.

(34) Hooijer, R.; Weis, A.; Biewald, A.; Sirtl, M. T.; Malburg, J.; Holfeuer, R.; Thamm, S.; Amin, A. A. Y.; Righetto, M.; Hartschuh, A.; et al. Silver-Bismuth Based 2D Double Perovskites $(4FPEA)_4AgBiX_8$ (X = Cl, Br, I): Highly Oriented Thin Films with Large Domain Sizes and Ultrafast Charge-Carrier Localization. *Adv. Opt. Mater.* **2022**, *10* (14), 2200354. DOI: 10.1002/adom.202200354.
